# Binding to SMN2 pre-mRNA-protein complex elicits specificity for small molecule splicing modifiers

**DOI:** 10.1038/s41467-017-01559-4

**Published:** 2017-11-14

**Authors:** Manaswini Sivaramakrishnan, Kathleen D. McCarthy, Sébastien Campagne, Sylwia Huber, Sonja Meier, Angélique Augustin, Tobias Heckel, Hélène Meistermann, Melanie N. Hug, Pascale Birrer, Ahmed Moursy, Sarah Khawaja, Roland Schmucki, Nikos Berntenis, Nicolas Giroud, Sabrina Golling, Manuel Tzouros, Balazs Banfai, Gonzalo Duran-Pacheco, Jens Lamerz, Ying Hsiu Liu, Thomas Luebbers, Hasane Ratni, Martin Ebeling, Antoine Cléry, Sergey Paushkin, Adrian R. Krainer, Frédéric H.-T. Allain, Friedrich Metzger

**Affiliations:** 1 F. Hoffmann-La Roche Ltd., Pharma Research & Early Development, Roche Innovation Center Basel, Grenzacherstrasse 124, Basel, 4070 Switzerland; 2 Cold Spring Harbor Laboratory, P.O. Box 100, 1 Bungtown Road, Cold Spring Harbor, New York, NY 11724 USA; 3 Department of Biology, ETH Zurich, Hönggerbergring 64, Zürich, 8093 Switzerland; 4 SMA Foundation, 888 Seventh Avenue, Suite 400 New York, New York, NY 10019 USA

## Abstract

Small molecule splicing modifiers have been previously described that target the general splicing machinery and thus have low specificity for individual genes. Several potent molecules correcting the splicing deficit of the *SMN2* (survival of motor neuron 2) gene have been identified and these molecules are moving towards a potential therapy for spinal muscular atrophy (SMA). Here by using a combination of RNA splicing, transcription, and protein chemistry techniques, we show that these molecules directly bind to two distinct sites of the *SMN2* pre-mRNA, thereby stabilizing a yet unidentified ribonucleoprotein (RNP) complex that is critical to the specificity of these small molecules for *SMN2* over other genes. In addition to the therapeutic potential of these molecules for treatment of SMA, our work has wide-ranging implications in understanding how small molecules can interact with specific quaternary RNA structures.

## Abstract (web summary)

Small molecules correcting the splicing deficit of the survival of motor neuron 2 (*SMN2*) gene have been identified as having therapeutic potential. Here, the authors provide evidence that SMN2 mRNA forms a ribonucleoprotein complex that can be specifically targeted by these small molecules.

## Introduction

Spinal muscular atrophy (SMA) is a rare genetic disease caused by the mutation or deletion of the survival of motor neuron 1 (*SMN1*) gene. SMA is characterized by progressive degeneration of spinal motoneurons, leading to muscle weakness and atrophy. Humans carry two nearly identical copies of the SMN genes: *SMN1* and *SMN2*. *SMN1* expresses full-length SMN protein, whereas *SMN2* exon 7 is predominantly skipped, due to a C-to-T transition at position 6 (C6U in the transcript) of exon 7, such that the mRNA is translated into a truncated and unstable protein^1, 2^. Regulation of *SMN2* exon 7 splicing is one of the best studied alternative splicing processes, and is an attractive therapeutic target. With the rise in genomic sequencing, the number of identified mutations affecting splicing has increased to over half of all disease-causing mutations^3^. Thus, approaches targeting splicing are becoming more relevant for drug development to treat splicing related disorders.

Alternative splicing in eukaryotes evolved to increase the coding potential of the genome by allowing the expression of multiple mRNA isoforms from a single gene. Splicing is a process that requires five snRNAs and more than 100 proteins to recognize sequence-specific sites and for catalysis. Additional regulatory splicing factors influence the extent of exon inclusion and intron excision. Splicing can be controlled individually for each gene and portions thereof, including decisions on exon inclusion, exon skipping, intron retention, recruitment of alternative 5′ and 3′ splice sites (5′ss and 3′ss), and mutually exclusive exons^4^. In general, intron removal is performed by the spliceosome, whose assembly involves recognition of the 5′ss, the 3′ss and the branch point sequence by U1 snRNP, U2AF, and U2 snRNP, respectively, among other factors. Several steps in this process may fail as a result of mutations in the coding sequence of an exon.

Various mechanisms have been proposed for how the C6U transition in the *SMN2* gene leads to exon 7 skipping, including disruption of an exonic splicing enhancer (ESE, recognized by SRSF1/SF2/ASF), gain of an exonic splicing silencer (recognized by hnRNP A1), and/or strengthening of an inhibitory stem–loop structure (TSL1) at the 3′ss of exon 7^5–8^. The stem–loop structure TSL2 at the 3′ end of exon 7 is also thought to partially sequester the 5′ss of exon 7^9, 10^.

*SMN2* exon 7 comprises three ESE regions, named for their ability to promote exon 7 inclusion. The ESE2 region of *SMN2* exon 7 is particularly relevant for this study. A variety of trans-acting factors were found to interact with exon 7. Tra2-β1, for example, is a splicing activator that interacts with a GAA motif in ESE2, with the G residue at position 22 being especially critical for stimulating exon 7 inclusion^11^. Overexpression of the splicing proteins SRSF9 (SRp30c) and hnRNP G, which interact with Tra2-β1, also promotes exon 7 inclusion. HnRNP G interacts with an A tract sequence upstream of the Tra2-β1 binding site^12^. Although Tra2-β1 has been recently shown to be dispensable^13^, hnRNP G and Tra2-β1 in combination are thought to strongly enhance the efficiency of exon 7 inclusion^12, 14^. These and other observations suggest that *SMN2* alternative splicing is coordinated by multiple sets of RNA–protein (RNP) interactions that likely involve higher-order folding of the pre-mRNA.

Several approaches that therapeutically target alternative splicing of *SMN2* are currently in various stages of development. These approaches range from an antisense oligonucleotide (SPINRAZA^®^) that has already received marketing approval to small molecules, all of which promote exon 7 inclusion and increase functional SMN protein levels. NVS-SM1 is a small molecule *SMN2* splicing modifier that acts specifically at the 5′ss of exon 7 to enhance binding affinity of the U1 snRNP, stabilizing the imperfect RNA helix formed by *SMN2* pre-mRNA and the U1 snRNP complex^15^.

In 2014, we reported the identification of highly selective *SMN2* splicing modifiers^16^. In the present study, we investigate their mechanism of action and find that the SMN-C class of molecules function via a specific interaction with an mRNA–protein complex comprising a primary splice site, a downstream exonic splicing enhancer region, and trans-acting splicing proteins at these locations on the mRNA. Combining different techniques in RNA biology and protein chemistry, including in vitro splicing assays, chemical proteomics, nuclear magnetic resonance (NMR), and surface plasmon resonance (SPR), we are able to identify that these small molecules function via interaction with a tertiary RNA structure comprising the ESE2 region of exon 7, and an RNA helix (formed by the 5′ss in intron 7 and the 5′ terminus of U1 snRNA) that binds the U1 snRNP complex. This interaction of small molecules with the mRNA–protein complex is critical for the high selectivity of the small molecules for *SMN2*, which is higher than that of compounds that interact only at the 5′ss site. Such selectivity of small molecules for complex, quaternary RNP structures has implications for therapeutic development in other diseases, potentially relevant in cases of previously considered undruggable small molecule targets.

## Results

### *SMN2* splicing modifiers modulate pre-mRNA splicing

**Fig. 1:**
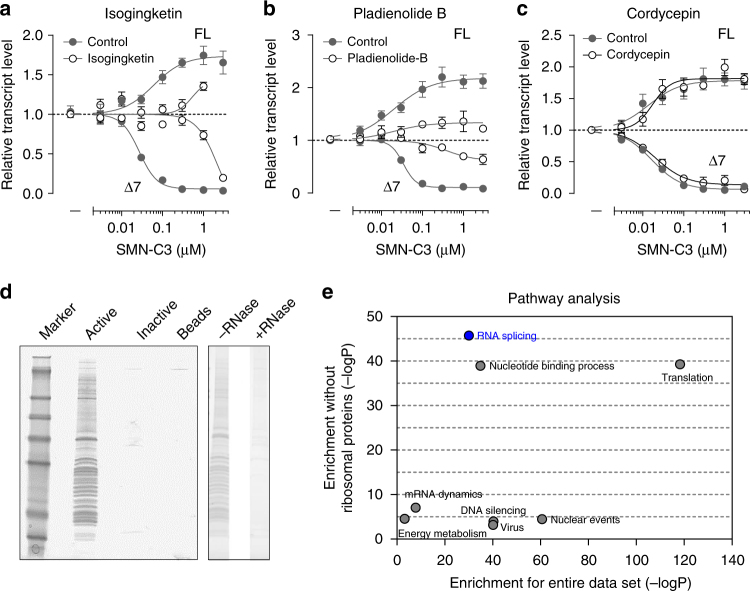
Small molecule splicing modifiers act directly on the RNA splicing process. **a**, **b**, **c** RT-PCR analysis of *SMN2* mRNA FL and ∆7 relative transcript levels in SMA type 1 patient fibroblasts. SMN-C3 compound activity after 6 h treatment was investigated in the presence or absence of inhibitors of splicing or polyadenylation, namely 50 µM isogingketin **a**, 100 nM pladienolide B **b**, or 40 µM cordycepin **c**. **d** Coomassie gel after chemical proteomics showing proteins pulled down in the presence of Active vs. Inactive compound, as well as after RNase treatment. The full gel is illustrated in Supplementary Fig. 2H. **e** Pathway enrichment analysis of Active compound (SMN-C6), suggesting highest enrichment of mRNA splicing complexes by Active, together with other categories. Removal of ribosomal proteins from the list confirmed a selective enrichment of proteins involved in mRNA splicing or nucleotide binding. Data in **a**, **b**, **c** represent means ± standard error of the mean (SEM) of at least nine independent assessments per concentration

We first hypothesized that the *SMN2* splicing modifiers might functionally interact with the splicing process in cells. We therefore investigated the activity of SMN-C3^16^ in a whole cell assay using SMA type 1 patient fibroblasts in combination with the pharmacological blockade of biological processes from DNA transcription to protein translation (Supplementary Fig. 1). Inhibitors of spliceosome assembly on pre-mRNA, with pharmacological inhibitors such as isoginkgetin^17^ or pladienolide B^18^, strongly reduced SMN-C3 activity, affecting both full-length and exon-7-lacking *SMN2* mRNA to similar degrees (Fig. 1a, b). In contrast, blocking polyadenylation with cordycepin^19^ (Fig. 1c) or inhibition of protein translation by puromycin^20^ (Supplementary Fig. 2C) did not affect SMN-C3 activity. Treatment with actinomycin D or α-amanitin, inhibitors of RNA polymerase II^21^, reduced SMN-C3 activity (Supplementary Fig. 2A, B) and also decreased baseline amounts of *SMN2* pre-mRNA (Supplementary Fig. 2D, E); this is known to generally occur concurrently with RNA polymerase II transcription^22^. These data indicate that the *SMN2* splicing modifiers act on the pre-mRNA splicing process but not on transcription or translation.

To further confirm the results with the classic pharmacological inhibitors, we used cell-free HeLa nuclear extracts and performed in vitro splicing with the structurally similar pharmacophore splicing modifiers, SMN-C3 and SMN-C5 (Supplementary Fig. 2G). SMN-C5 showed a concentration-dependent correction of *SMN2* splicing to increase exon 7 inclusion (Supplementary Fig. 2G), whereas SMN-C3 did not show a change in this experimental assay. Given the similar pharmacophore of SMN-C5 and SMN-C3 and very similar potency in cellular assays (EC_50_ 17 nM for SMN-C3 (Supplementary Fig. 2A), 15 nM for SMN-C5 (Supplementary Fig. 3A), the result was unanticipated but explicable by the particular physicochemical properties of the molecules. SMN-C5 has a much higher permeability (assessed in the PAMPA assay) compared to SMN-C3, as well as a much lower amphiphilicity, two properties that may explain the difference in potency especially at higher concentration in cell-free assays such as the in vitro splicing assay.

To further understand this effect at the molecular level, we utilized chemical proteomics to identify proteins that may bind to the *SMN2* splicing modifiers. We generated active and inactive ligands based on the structure of SMN-C5. This was initiated firstly because of the better physicochemical properties of SMN-C5, and secondly due to the chemical structure of SMN-C5, which as opposed to SMN-C3, is amenable to side group modifications required for ligand immobilization without losing biological activity (Supplementary Fig. 3). For pull-down experiments, either the Active (SMN-C6) or the Inactive (SMN-C7) derivatives of SMN-C5 were immobilized on Sepharose beads. Using cellular extracts from SMA type 1 patient fibroblasts, a strong and RNase-sensitive enrichment of proteins with the active, but not the inactive ligand, was observed (Fig. 1d). We identified 430 proteins enriched on affinity matrices with the active vs. the inactive ligand (*p*-adj < 0.05; log2 fold change > 1.5, Supplementary Table 1). Following pathway-based enrichment analyses of the chemical proteomics data, a strong enrichment of proteins involved in RNA splicing was observed (Fig. 1e). All five major spliceosomal snRNPs were represented and specifically enriched with the active vs. the inactive ligand (Supplementary Fig. 4A, B). In addition, mature mRNAs of *SMN2* and *STRN3*, the two major hits identified previously^16^, were enriched in cellular extracts incubated with the active ligand (Supplementary Fig. 4C). Taken together, these data suggest that the splicing modifiers influence alternative splicing by directly interacting with the splicing machinery, in the presence of RNA.

### *SMN2* splicing modifiers bind to the U1 snRNA:5′ss duplex

**Fig. 2:**
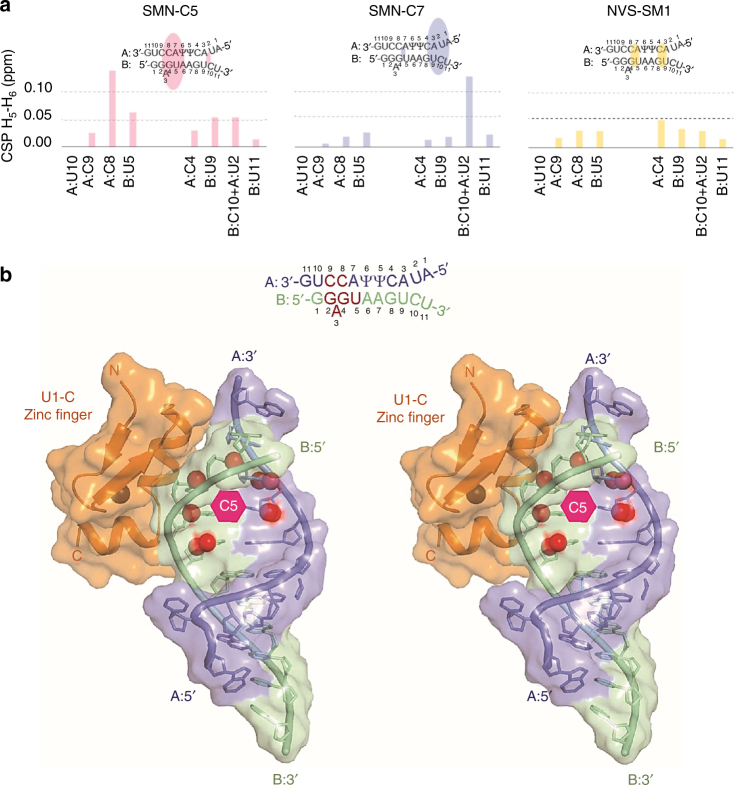
Binding of splicing modifiers to RNP complex. **a** Binding of three compounds (SMN-C5/active, SMN-C7/inactive, and NVS-SM1) to the RNA duplex formed by the 5′ss and the 5-end of U1 snRNA was analyzed by solution-state^1^H NMR. The chemical-shift perturbations (CSP) of the H5-H6 protons of the pyrimidine bases are plotted as a function of the sequence. SMN-C5, but not SMN-C7 or NVS-SM1 binding to the RNA duplex was observed preferentially upstream of the pseudouridine pair in the region where the last adenine of exon 7 is bulged out, a hotspot highlighted for its ability to lower the splicing efficiency of *SMN* exon 7. **b** Cross-eyed stereo view of a NMR-guided model of the U1-5′ss RNA duplex in which U1-C zinc finger domain was modeled at the same interface as in the structure of U1 snRNP^26, 27^. In this model, protons strongly affected upon addition of SMN-C5 are represented by red beads, all of which define the binding interface of SMN-C5, located in the major groove of the RNA helix, whereas U1-C binds the minor groove. The molecule SMN-C5 is represented by the pink hexagon. Both binding interfaces are located on opposite sites, around the invariant GU motif of the 5′ss. The sequence of the RNA is given at the top of the figure. The U1 snRNA-derived oligonucleotide, the 5′ss, and U1-C are colored in blue, green, and orange, respectively

The 5′ss in the *SMN2* gene is inherently weak at interacting with the U1 snRNA component of U1 snRNP^23^. Recently published data suggested that NVS-SM1, a different class of compound, increases the binding affinity of U1 snRNP for the 5′ss of exon 7^15, 16^. Thus, we investigated whether SMN-C5 interaction with 5′ss directly or indirectly influences the binding affinity of spliceosomal U1 snRNP. For this purpose, the binding potential to the 5′ss of three splicing modifiers, SMN-C5 (Active), SMN-C7 (Inactive), and NVS-SM1 was monitored in the presence of an *SMN2* pre-mRNA fragment and U1 snRNA using solution-state^1^H NMR. Binding of all three compounds induced changes in the resonance of the RNA duplex formed by the 5′-terminus of U1 snRNA and the 5′ss of *SMN2* pre-mRNA (Supplementary Fig. 5A, B). Whereas SMN-C7 and NVS-SM1 mainly bound downstream (further into the intron) of the consecutive pseudouridine:adenine pairs in the U1 snRNA:5′ss duplex, SMN-C5 bound preferentially upstream, in the region where the last adenine of exon 7 (which does not fit the 5′ss consensus) is protruding, a spot known for its ability to lower the splicing efficiency of SMN exon 7 (Fig. 2a, b)^7, 23–25^.

According to the chemical-shift perturbations we observed, SMN-C5 targets the major groove of the RNA duplex between the 5′ss *SMN2* fragment and U1 snRNA (Fig. 2b, Supplementary Fig. 5D) and induces the broadening of one imino signal, in line with binding in the proximity of the base pairs (Supplementary Fig. 5A). In the context of intact U1 snRNP, the U1 snRNA:5′ss duplex is stabilized by the zinc finger domain of U1-C, a subunit of U1 snRNP, which contacts both RNA strands via their ribose-phosphate backbone^26, 27^. We performed NMR titration on the same RNA duplex already bound by the zinc finger domain of U1-C. Very similar chemical-shift changes of the RNA were seen upon addition of SMN-C5, consistent with SMN-C5 binding in the absence of protein, and without affecting U1-C binding (Supplementary Fig. 5C). The binding interfaces of SMN-C5 and U1-C are located on opposite sites around the invariant GU motif of the 5′ss (Fig. 2b). Taken together, the NMR analyses revealed that SMN-C5 binds the U1:5′ss duplex at a different site than SMN-C7 or NVS-SM1, and its binding site is not affected by the presence of the U1-C zinc finger.

### ESE2 interactions determine splicing modifiers specificity

**Fig. 3:**
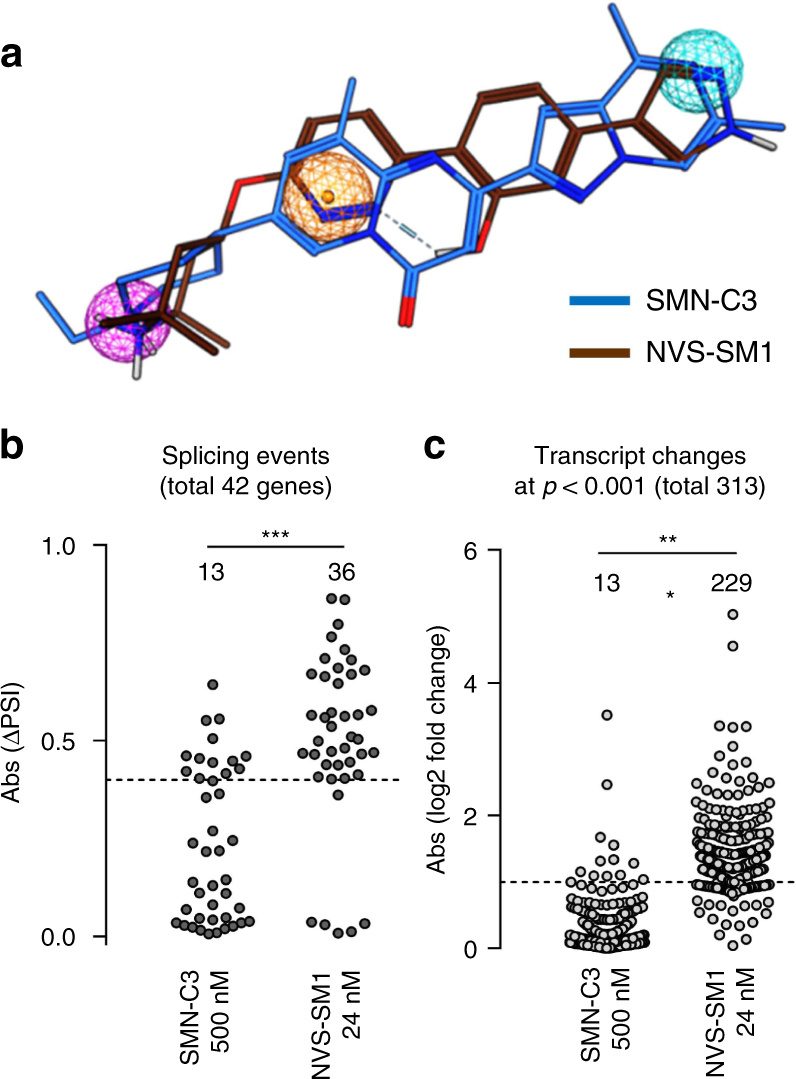
SMN-C3 and NVS-SM1 share a pharmacophore, but show different selectivity. **a** Pharmacophore model and alignment of SMN-C3 (blue) and NVS-SM1 (brown) with MOE2014.09. **b**, **c** RNA-seq analysis in type 1 SMA patient fibroblasts treated with SMN-C3 (500 nM) or NVS-SM1 (24 nM) for 24 h. **b** Transcriptome-wide splicing events for all treatment conditions with a ∆PSI of >0.4 (total of 42). **c** Transcriptome-wide gene expression changes significant at the *p* = 0.001 level in any of the treatment conditions (total of 313). Data in **b** and **c** represent mean values of 5 independent replicates. **p < 0.01, ***p < 0.001 as estimated by Student’s *t* test

Having confirmed the binding of both classes of *SMN2* splicing modifiers to the U1 snRNA:5′ss duplex—albeit at different positions—and given the high overlap of the pharmacophores of SMN-C class compounds and NVS-SM1 (Fig. 3a), we aimed to understand the differences in their selectivity for global splicing events and transcriptional changes^15, 16^. We performed transcriptome-wide sequencing of RNA from type 1 SMA patient fibroblasts treated with SMN-C3 at ~50 times the EC_50_ (500 nM, Supplementary Fig. 6A) or NVS-SM1 at ~5 times the EC_50_ (24 nM, Supplementary Fig. 6B). A database of alternative splicing events was generated, based on the human RefSeq database of transcripts (Release 66, August 2014). Splicing events were characterized by a PSI (“percent spliced in”) score, and changes compared to matched controls were determined by ∆PSI values. In total, 42 transcriptome-wide splicing event changes were observed under either treatment condition, with a ∆PSI of > 0.4 (Fig. 3b, Supplementary Table 2), with NVS-SM1 affecting 36 transcripts and SMN-C3 only 13 (Supplementary Fig. 6C). In addition, transcriptional profiling analysis demonstrated expression changes in 313 genes overall, considering both treatment conditions (Fig. 3c; Supplementary Table 3), with 229 expression changes for NVS-SM1 as opposed to 13 for SMN-C3 (Supplementary Fig. 6D). These data demonstrate an unexpected higher selectivity of SMN-C3 compared to NVS-SM1, suggesting an additional mode of action than NVS-SM1’s previously described U1 snRNA:5′ss interaction alone^15^.

**Fig. 4:**
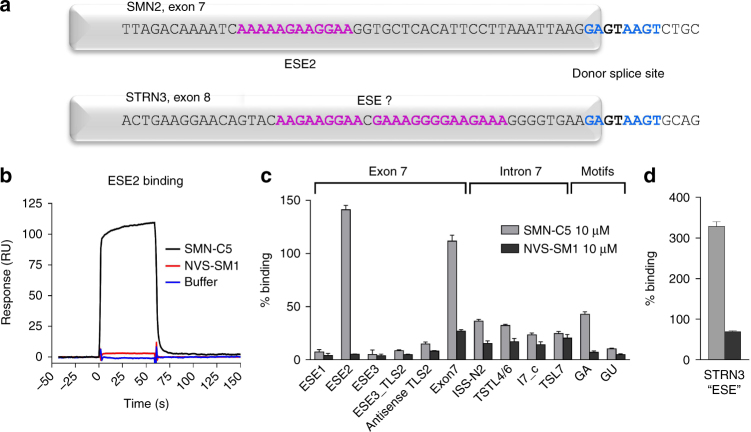
SMN-C5 binds to the SMN2 ESE2 and the STRN3 “ESE-like” structure. **a** Schematic illustration of the ESE2 region in the *SMN2* exon 7, and the putative ESE in the *STRN3* exon 8 (indicated in pink). Note that the 5′ss of these two exons are identical (in blue). **b** Direct binding analysis on a SPR system (Biacore 2000) to investigate the interaction between SMN-C5 or NVS-SM1 (10 µM each) and buffer control, to the immobilized ESE2 RNA in the *SMN2* gene. Representative sensorgrams from triplicate experiments are depicted. **c** Quantitative assessment of binding of SMN-C5 and NVS-SM1 (10 µM each) to distinct RNAs in the *SMN2* gene (ESE1, ESE2, ESE3, ESE3_TLS2, antisense_TLS2, Exon 7, ISS-N2, I7_TSTL4/6, I7_c, I7_TSL7, “GA” motif, and “GU” motif) immobilized on the SPR sensor. RNAs were captured via biotin on the streptavidin-coated sensor at surface densities in the range of 900–1200 RUs (response units). The SPR signals monitored for small molecules are normalized to the molecular mass. 100% binding corresponds to the amplitude of SPR signal monitored for binding of a small molecule to the RNA strand by full occupancy of one binding site (1:1 binding stoichiometry). **d** Binding to the *STRN3* ESE-like region. Data in **c** and **d** represent means ± SEM of five replicates

This finding led us to investigate sequence homologies between putative target genes of the SMN-C class compounds. As previously shown, only one gene besides *SMN2*, namely *STRN3*, exhibited a strong magnitude of splicing change elicited by SMN-C3 in SMA type 1 patient fibroblasts. Apart from this, the other significant changes were strong splicing changes in a pseudogene (*PDXDC2P*) or the downregulation of the entire *PDXDC1* gene that did not affect its splicing (see Fig. 2b in ref. ^16^). We therefore searched for common sequence motifs around the alternatively spliced exon 8 in *STRN3*, and identified an identical weak 5′ss and a purine-rich region similar to the ESE2 motif in *SMN2* (Fig. 4a). We then tested the interaction of the splicing modifiers SMN-C5 (active), SMN-C7 (inactive), and NVS-SM1 with this ESE motif, utilizing real-time label-free SPR technology. We monitored the binding of these molecules to surface-immobilized *SMN2* exon 7 and *STRN3* exon 8 RNA fragments. SMN-C5 strongly bound to ESE2, whereas NVS-SM1 showed no binding at all (Fig. 4b). We extended our analysis to other distinct RNA cis elements within exon 7 and intron 7 of *SMN2*, and observed strong binding only to the full exon 7 (which includes ESE2) but little to no binding to any other individual element (Fig. 4c). An analysis of SMN-C5 binding to the putative ESE in the *STRN3* gene revealed even stronger binding than to *SMN2* ESE2 (Fig. 4d). NVS-SM1 did not show detectable binding to any element analyzed (Fig. 4c, d). SMN-C7 (Inactive), also bound to *SMN2* ESE2 and the putative ESE in the *STRN3* gene although less potently (Supplementary Fig. 7A, B), suggesting that binding to ESE2 is likely a specific property of the SMN-C class compounds.

Finally, we investigated the binding of the splicing modifiers to ESE2 using solution NMR spectroscopy. Chemical-shift perturbations of the RNA resonances were the largest upon addition of SMN-C5, and the weakest for NVS-SM1 (Supplementary Fig. 7C), confirming the direct binding of the SMN-C compounds to the ESE2 RNA. Moreover, only the addition of SMN-C5 induced the formation of broad imino signals in the proton 1D spectrum of the complex ESE2:SMN-C5 (Supplementary Fig. 7D), indicating that SMN-C5 may induce a conformational shift by binding to ESE2. In conclusion, both SPR and NMR analyses confirmed that SMN-C class compounds bind to ESE2, in contrast to NVS-SM1.

### Requirement of ESE2 and the 5′ss for SMN-C5 activity

**Fig. 5:**
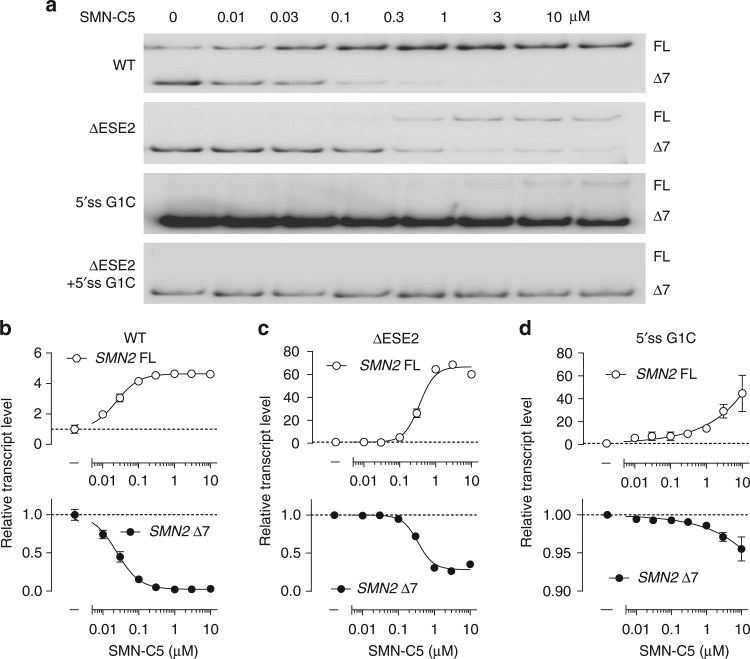
Requirement of both 5′ss and ESE2 mutation/deletion for full abolishment of SMN-C5 activity. **a** Cell-based splicing assay of *SMN2* minigene constructs in HEK293 cells treated with increasing concentrations of SMN-C5 (0.01–10 µM) in wild-type constructs and in constructs harboring a 5′ss mutation (G1C), a deletion of the ESE2 region (ΔESE2) or both 5′ss G1C and ΔESE2. In the double-mutant construct (ΔESE2 + 5′ss G1C), the SMN-C5 effect was completely abolished. Full blots shown in Supplementary Fig. 8. **b**, **c**, **d** Quantitative assessment of the data by RT-qPCR. **b** Wild-type (WT), **c** ESE2 deletion, **c** 5′ss G1C point mutation construct. Double mutation of 5′ss and ESE2 did not yield quantifiable results. Data represent means ± SEM of three independent transfections

Having shown binding of SMN-C5 to single-stranded ESE2 RNA and to the U1 snRNA:5′ss RNA duplex separately, we further investigated if both the regions are important for SMN-C5 activity. Therefore, we assessed SMN-C5 splicing activity via cell-based splicing assays using *SMN2* minigene constructs with ESE2 deletion and/or 5′ss mutation. SMN-C5 promoted exon 7 inclusion in the construct with a deletion of the ESE2 region (ΔESE2, Fig. 5a, c), although less efficiently (at higher compound concentrations) than in cells expressing the wild-type construct (Fig. 5b). For the construct carrying a 5′ss mutation (G1C), there was very little but detectable promotion of exon 7 inclusion with SMN-C5 (Fig. 5d), suggesting that the 5′ss is the major site of action. However, only in the double-mutant construct (ΔESE2 + 5′ss G1C), SMN-C5 was completely ineffective (Fig. 5a, bottom, no quantification possible). It should be noted, however, that splicing of the double mutant is severely affected regardless of treatment, consistent with the severity of the mutations created at both the 5′ss and an important exonic splicing enhancer region. In aggregate, these data suggest that both the 5′ss and ESE2 are important sites of SMN-C5 activity, to promote exon 7 inclusion in *SMN2* transcripts.

Our investigations identified a small molecule that enhances 5′ss recognition by targeting the major groove of the RNA complex. We therefore sought to define protein components of the target complex with ESE2, and used the ESE2 sequence as bait to capture proteins from nuclear extracts of SMA type 1 patient fibroblasts, in the presence of either Active (SMN-C6) or Inactive (SMN-C7) ligands. SMN-C6 but not SMN-C7 induced the binding of 10 proteins with the ESE2 RNA (*p*-adj < 0.05, unique peptides ≥ 2). Seven of these proteins are RNA-binding proteins and two are part of U1 snRNP, namely the U1-A and U1-70K proteins (based on String functional protein associate analysis, Supplementary Fig. 9). An interaction between the ESE2 sequence and U1 snRNP has not been, to the best of our knowledge, previously described.

**Fig. 6:**
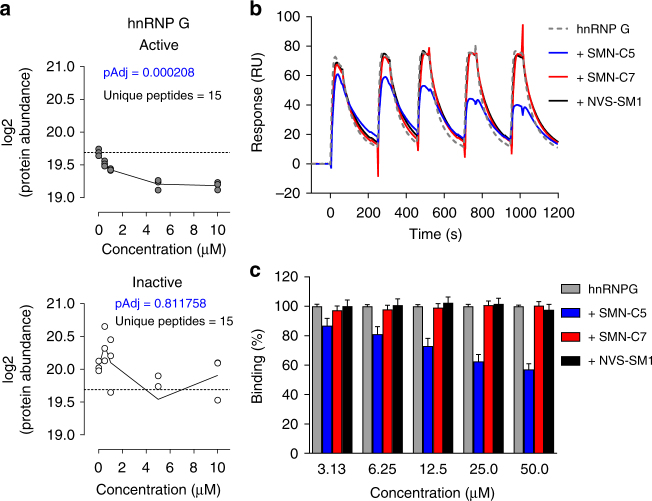
Displacement of hnRNP G from *SMN2* exon 7 ESE2 by small molecule splicing modifiers. **a** Protein abundance (log2) of hnRNP G after affinity enrichment with the *SMN2* ESE2 RNA sequence as bait, in the presence of increasing concentrations of Active (SMN-C6) or Inactive (SMN-C7) ligand. **b** SPR-binding curves monitored for hnRNP G RRM over ESE2 surface at a single hnRNP G concentration of 1.5 µM (green) and competition of hnRNP G-ESE 2 interaction by SMN-C5 (blue), SMN-C7 (red), or NVS-SM1 (black). ESE2 was immobilized on a streptavidin surface (CAP sensor) to a density of ~25 RU. Small molecules were titrated from 3.13 to 50 µM at constant hnRNP G concentration (1.5 µM). **c** Quantitative assessment of normalized SPR signals of competition experiments in **b**. SPR signals were measured in the middle of the association phase and normalized according to immobilized levels of ESE2. 100% binding corresponds to the binding of hnRNP G to ESE2 at 1.5 μM. Data in **c** represent means ± SEM of five independent experiments illustrated in **b**

We also identified RBMX (also known as hnRNP G) protein as one of the 10 proteins interacting with the SMN-C6–ESE2 complex. The hnRNP G protein binds in the ESE2 region of *SMN2* exon 7 and is known as a positive regulator of *SMN2* exon 7 inclusion^12^. However, the mode of action of hnRNP G in *SMN2* exon 7 splicing regulation is still not fully understood^11, 12^. When tested in competitive binding assays, hnRNP G was displaced from ESE2 by an excess of active SMN-C6 (Fig. 6a), whereas Tra2-β1 and SRSF9 were not (Supplementary Fig. 10A, B), suggesting a direct interaction of SMN-C6 at the RNA-interaction site of hnRNP G. To confirm this unexpected displacement of hnRNP G at the ESE2 site by splicing modifier binding, we performed SPR-binding analyses of hnRNP G RRM to ESE2 RNA immobilized on a Biacore sensor chip. Titration experiments confirmed the binding of hnRNP G to ESE2 RNA (Supplementary Fig. 10C, D). When incubated in the presence of micromolar concentrations of either SMN-C5, SMN-C7, or NVS-SM1, we observed that only SMN-C5 but not SMN-C7 or NVS-SM1 partially competed with the binding of hnRNP G RRM to ESE2 (Fig. 6b, c), suggesting that binding of hnRNP G to ESE2 seems to be partially inhibited in the presence of the Active compound. Furthermore, we observed NMR chemical-shift perturbations towards the free-form state of the hnRNP G protein when hnRNP G’s RRM bound to RNA was titrated with SMN-C5 also at high concentrations (Supplementary Fig. 11B). In contrast, we observed no difference in NMR spectra with the Tra2-β1–RNA complex (Supplementary Fig. 11A), indicating that SMN-C5 does not affect the interaction of Tra2-β1 with the ESE2 RNA. The absence of chemical-shift perturbation with SMN-C7 (Supplementary Fig. 11C) indicates that the effect on hnRNP G is specific to the biological activity of the splicing modifier, suggesting that the SMN-C class compounds may bind at the ESE2 region in *SMN2* and displace hnRNP G from its ESE2-binding site. The partial displacement of hnRNP G from the ESE2 region along with the pull-down of hnRNP G in chemical proteomics experiments in the presence of SMN-C5 suggests a direct interaction of the compound at the RRM-binding site of hnRNP G; whether or not the partial displacement observed in the in vitro systems translates into the more complete in vivo context is beyond the scope of this study. There are several key hypotheses that could explain the increasing selectivity of SMN-C5, relative to other splicing modifiers, influenced by its interaction at the ESE2 region, which we will address in the discussion section.

## Discussion

In Naryshkin et al. 2014^16^, we introduced a compound class of orally bioavailable splicing modifiers (SMN-C class) that promote *SMN2* exon 7 inclusion, and hypothesized that they are involved in specific protein–RNA and/or RNA–RNA interactions. Using cell-based in vitro splicing assays, SPR assays, solution-state NMR, and chemical proteomics, we have now delineated that the SMN-C class splicing modifiers stabilize an RNP complex that is apparently unique to the transcripts of the *SMN2* and *STRN3* genes.

We have analyzed the modifier’s effects on various cis-acting elements and the corresponding trans-acting splicing factors involved in *SMN2* alternative splicing regulation. One way that cis-acting sequences are known to influence exon 7 splicing is by binding splicing activators that help to recruit spliceosomal snRNPs^28^. As the 5′ss of exon 7 in *SMN2* is a weak splice site known for its suboptimal U1 snRNP binding, this may be a limiting step in exon 7 inclusion efficiency^8, 23, 29^. In this context, NVS-SM1, an *SMN2* splicing modulator with a similar pharmacophore as the SMN-C class of compounds (Fig. 3a), was proposed to act by increasing the interaction of U1 snRNP with the 5′ss^15^. Our analyses of solution-state NMR confirmed that both NVS-SM1 and the SMN-C class of splicing modifiers bind to the 5′ss of *SMN2* exon 7, in agreement with the proposed model. However, the precise site of action of SMN-C5 appears to be a few nucleotides upstream in the 5′ss, compared to NVS-SM1. In addition, we observed direct binding of SMN-C5 to the major groove of the U1 snRNA:5′ss RNA duplex, close to the bulged B:A_3_ (Fig. 2b), a region already known for its role in the low splicing efficiency of exon 7^29^. Thus, the two structurally related compound classes share the mechanism of enhancing U1 snRNP affinity to the 5′ss, thereby making exon 7 inclusion more efficient. The inactive SMN-C7 bound to a slightly more downstream intronic region, demonstrating that the interaction with the proximal region of the 5′ss seems to be important for the compound′s biological activity.

By transcriptome-wide RNA-sequencing, we demonstrated higher selectivity of SMN-C3 than NVS-SM1 on both transcript splicing and expression levels. As the weak *SMN2* exon 7 5′ss is prevalent in many other genes^15^, inducing the U1 snRNA:5′ss interaction alone would be insufficient to explain the high selectivity, suggesting an additional mechanism of action for the SMN-C class compounds. As previously reported, the SMN-C class of compounds modifies splicing of a single additional gene, *STRN3*, to a similar extent as *SMN2* ^16^. Upon examination of common sequence motifs, we identified a purine-rich putative ESE region similar to ESE2 in *SMN2*, and confirmed the binding of the SMN-C class of splicing modifiers to this region in *STRN3*, as well as in *SMN2*. In addition, chemical proteomics experiments demonstrated that the ESE2 sequence pulled down the U1 snRNP proteins U1-A and U1-70K, as well as hnRNP G, in the presence of the active SMN-C6. These results support the hypothesis that the compounds can directly bind and potentially alter *SMN2* RNA structures, thereby influencing their interactions with key trans-acting splicing factors.

Moreover, the data strongly suggest the existence of a quaternary RNP complex comprising the ESE RNA sequence, the 5′ss, at least one SR protein, and U1 snRNP in the presence of the splicing modifier. It was therefore important to understand how the *SMN2* splicing modifiers affect binding of previously identified trans-acting splicing factors to the *SMN2* pre-mRNA. Tra2-β1, a well characterized splicing activator, showed no change in ESE2 binding in the presence of active SMN-C class compound, whereas hnRNP G was partially displaced by SMN-C5. The protein hnRNP G binds to part of the ESE2 region through interactions with Tra2-β1, and acts additively with Tra2-β1 to promote exon 7 inclusion^5, 12, 14, 30, 31^. As shown previously, exon 7 inclusion increases from ~20 to ~65% after overexpression of hnRNP G alone^12, 14^, and hnRNP G and Tra2-β1 synergistically enhance exon 7 inclusion to up to 80% when overexpressed simultaneously^14, 32^.

**Fig. 7:**
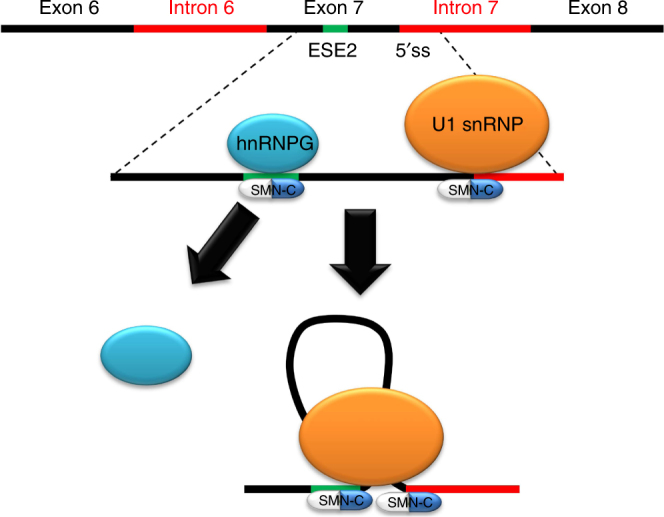
Working model of the proposed molecular interactions of the SMN-C type *SMN2* splicing modifiers. SMN-C type compounds bind to two sites at the *SMN2* exon 7, namely ESE2 and 5′ss when U1 snRNA is bound. Whereas interaction with the 5′ss drives promotion of exon 7 inclusion in the splicing process, binding to ESE2 results in hnRNP G dislocation and allows the U1 snRNP complex to bind to ESE2. Both binding processes in combination provide high selectivity to the *SMN2* and *STRN3* pre-mRNAs over other genes

The experimental displacement of hnRNP G in the presence of an SMN-C class compound, as shown in the competitive binding assays, highlights the interaction of SMN-C6 with hnRNP G and ESE2—by directly altering the RNA-binding site or altering the RNA structure to which hnRNP G normally binds. The mode of action of hnRNP G in SMN2 exon 7 splicing regulation is still not fully understood, and it is therefore difficult to speculate on the mode of action of the active molecule in this context. However, it is plausible that hnRNP G may bind the ESE2 sequence around the inhibitory stem loop (TSL1), thereby disrupting this structure and improving the weak 3′ss region to further enhance exon inclusion. The active molecule could then play the role of hnRNP G with a higher efficiency by stabilizing the formation of an alternative RNA base-pairing that further reduces the inhibitory effects of the TSL1. Another attractive hypothesis would be that the molecule simply reorganizes the positioning of hnRNP G on the exon 7 and reinforces its activity as a splicing activator. In addition, binding of the active compound to the hnRNP G-binding site can result in interaction with other splicing enhancers, which we demonstrated by the chemical proteomics and pull-down studies, showing that Active SMN-C6 binding to ESE2 recruit proteins involved in the larger spliceosomal complex. Finally, we hypothesize that possibly more than one small molecule interacts simultaneously with the ESE and the 5′ splice site duplex (Fig. 7). Rigorously testing these putative mechanisms warrants further investigation.

In summary, our data show that in addition to the U1 snRNP:5′ss interaction in *SMN2* exon 7, U1 snRNP can also interact directly or indirectly with the ESE2 motif, and these interactions can be modulated by small molecules in a coordinated fashion thus providing specificity towards regulation of SMN2 alternative splicing (Fig. 7). Our data suggest a potential formation of an RNA tertiary structure with the 5′ss, explaining the high selectivity of the SMN-C class of compounds, with splicing of only two pre-mRNAs being affected at physiological concentrations and further substantiated by the similarity of *STRN3* and *SMN2* (Fig. 4a).

Our report suggests that a splicing deficit of an individual gene can be selectively targeted through specific small molecule interactions with RNA:protein complexes. As further findings highlight mis-splicing to be the cause of many disease-causing mutations^3, 33^, the work presented here has the potential to have widespread implications in the research and development of such RNA-targeting therapies.

## Methods

### Selection of chemical class of splicing modifiers

Both compounds SMN-C3 and SMN-C5 belong to the same chemical class. SMN-C5 was prepared on the same basis as C3 and shares the exact same pharmacophore. The two compounds SMN-C3 and SMN-C5 have a perfect overlay of every critical atom being exactly in the same position, with exactly the same fully planar spacer. These similarities provide the similar selectivity and specificity. The active ligand out of SMN-C5 needed for the chemical proteomics pull-down experiments required the generation of SMN-C6L, which was modified at the end where biological activity was minimally affected.

### Preparation of chemical proteomics ligands

The SMN splicing modifiers SMN-C6 and SMN-C6L and their negative controls SMN-C7 and SMN-C7L were synthesized from the published corresponding N-H piperazine derivate, 2-(8-fluoro-2-methylimidazo[1,2-a]pyridin-6-yl)-7-(piperazin-1-yl)-4H-pyrido[1,2-a]pyrimidin-4-one^34^ (CAS: 1449592-34-0) by alkylation with Boc-protected aminopropylbromide and subsequent deprotection yielded SMN-C6. This compound in turn was acylated with pentanoyl chloride to yield SMN-C6L. SMN-C7 was prepared from the same starting material, the NH-piperazine, through coupling of 3-(tert-butoxycarbonylamino)propanoic acid with HBTU and subsequent deprotection and SMN-C7L through coupling with 3-pentanamidopropanoic acid. The synthesis details are provided as follows:

For preparation of 7-(4-(3-aminopropyl)piperazin-1-yl)-2-(8-fluoro-2-methylimidazo[1,2-a]pyridin-6-yl)-4H-pyrido[1,2-a]pyrimidin-4-one trihydrochloride (SMN-C6), in a first step, a suspension of 2-(8-fluoro-2-methylimidazo[1,2-a]pyridin-6-yl)-7-(piperazin-1-yl)-4H-pyrido[1,2-a]pyrimidin-4-one (CAS: 1449592-34-0, 119 mg, 314 µmol), tert-butyl 3-bromopropylcarbamate (85.8 mg, 346 µmol) and potassium carbonate (52.2 mg, 377 µmol) in dimethylacetamide (3.2 ml) was stirred over night at 60°C under stirring. The reaction was diluted with water, extracted twice with dichloromethane. The combined organic layer was washed once with saturated aqueous sodium chloride solution, dried over sodium sulfate, filtered and the solvent was evaporated under reduced pressure. The residue was purified by column chromatography on silica gel (CH2Cl2:MeOH 9:1) to yield the title compound as a yellow solid (95 mg, 56.4 %). MS ISN (m/e): 580.5 (100) [(M + HCOOH-H)-] to yield tert-butyl 3-(4-(2-(8-fluoro-2-methylimidazo[1,2-a]pyridin-6-yl)-4-oxo-4H-pyrido[1,2-a]pyrimidin-7-yl)piperazin-1-yl)propylcarbamate. In a second step, to a solution of tert-butyl 3-(4-(2-(8-fluoro-2-methylimidazo[1,2-a]pyridin-6-yl)-4-oxo-4H-pyrido[1,2-a]pyrimidin-7-yl)piperazin-1-yl)propylcarbamate (95 mg, 177 µmol) in dichloromethane (1.77 ml) was added hydrochloric acid 4 M in dioxane (222 µl, 887 µmol). The reaction was stirred at room temperature overnight. The reaction was diluted with diethyl ether and the precipitate was filtered off and dried to yield the title compound as a yellow powder (83 mg, 85.9 %). MS ISP (m/e): 436.3 (5) [(M + H) + ], 218.7 (100) [(M/2 + H) + ] to yield 7-(4-(3-aminopropyl)piperazin-1-yl)-2-(8-fluoro-2-methylimidazo[1,2-a]pyridin-6-yl)-4H-pyrido[1,2-a]pyrimidin-4-one trihydrochloride (SMN-C6).

A suspension of 7-(4-(3-aminopropyl)piperazin-1-yl)-2-(8-fluoro-2-methylimidazo[1,2-a]pyridin-6-yl)-4H-pyrido[1,2-a]pyrimidin-4-one (10 mg, 23.0 µmol) and triethylamine (6.97 mg, 9.55 µl, 68.9 µmol) in dimethylacetamide (0.3 ml) was added pentanoyl chloride (3.32 mg, 3.27 µl, 27.6 µmol) and stirred at room temperature for 1 h. The light yellow suspension was poured into 5 ml of water and extracted twice with dichloromethane. The organic layer was washed with saturated aqueous sodium chloride solution, dried over sodium sulfate, filtered and concentrated in vacuo to yield the title compound as a light yellow oil (4 mg, 33.5 %). MS ISP (m/e): 260.8 [(M/2 + H) + ] to further yield N-(3-(4-(2-(8-fluoro-2-methylimidazo[1,2-a]pyridin-6-yl)-4-oxo-4H-pyrido[1,2-a]pyrimidin-7-yl)piperazin-1-yl)propyl)pentanamide (SMN-C6L).

To prepare 7-(4-(3-aminopropanoyl)piperazin-1-yl)-2-(8-fluoro-2-methylimidazo[1,2-a]pyridin-6-yl)-4H-pyrido[1,2-a]pyrimidin-4-one dihydrochloride (SMN-C7), in a first step tert-butyl 3-(4-(2-(8-fluoro-2-methylimidazo[1,2-a]pyridin-6-yl)-4-oxo-4H-pyrido[1,2-a]pyrimidin-7-yl)piperazin-1-yl)-3-oxopropylcarbamate was generated. To a suspension of 2-(8-fluoro-2-methylimidazo[1,2-a]pyridin-6-yl)-7-(piperazin-1-yl)-4H-pyrido[1,2-a]pyrimidin-4-one (CAS: 1449592-34-0, 130.6 mg, 345 µmol) und 3-(tert-butoxycarbonylamino)propanoic acid (71.8 mg, 380 µmol) in DMA (1.72 ml) was added under stirring and an athmosphere of nitrogen triethylamine (105 mg, 144 µl, 1.04 mmol) and HBTU (200 mg, 518 µmol). The reaction was stirred over night at room temperature. Water was added and the reaction was extracted three times with methylene chloride. The combined organic layer was washed once with water, dried over sodium sulfate, filtered and the solvent was evaporated under reduced pressure. The residue was purified by column chromatography on silica gel (CH2Cl2 to CH2Cl2:MeOH 9:1) to yield the title compound as a yellow solid (152 mg, 80.1 %). MS ISP (m/e): 550.4 (100) [(M + H) + ], 225.7 (37) [(M/2 + H) + ]. In a second step, to a solution of tert-butyl 3-(4-(2-(8-fluoro-2-methylimidazo[1,2-a]pyridin-6-yl)-4-oxo-4H-pyrido[1,2-a]pyrimidin-7-yl)piperazin-1-yl)-3-oxopropylcarbamate (152 mg, 277 µmol) in dichloromethane (2.8 ml) was added under stirring a solution of 4 M hydrogen chloride solution in dioxane (346 µl, 1.38 mmol). The reaction was stirred over night at room temperature, diluted with diethyl ether and the precipitate was filtered off, washed with diethyl ether and dried in high vacuum at 45 °C to obtain the title compound as a yellow powder (141.4 mg, 97.9 %). MS ISP (m/e): 450.2 (5) [(M + H) + ], 225.7 (100) [(M/2 + H) + ] to yield to prepare 7-(4-(3-aminopropanoyl)piperazin-1-yl)-2-(8-fluoro-2-methylimidazo[1,2-a]pyridin-6-yl)-4H-pyrido[1,2-a]pyrimidin-4-one dihydrochloride (SMN-C7). Further, a yellow solution of 2-(8-fluoro-2-methylimidazo[1,2-a]pyridin-6-yl)-7-(piperazin-1-yl)-4H-pyrido[1,2-a]pyrimidin-4-one (10 mg, 26.4 µmol), 3-pentanamidopropanoic acid (4.58 mg, 26.4 µmol), triethylamine (13.4 mg, 18.3 µl, 132 µmol), and 2-(1H-benzo[d][1–3]triazol-1-yl)-1,1,3,3-tetramethylisouronium hexafluorophosphate(V) (13.0 mg, 34.4 µmol) in dimethylacetamide (0.3 ml) was stirred at 60 °C for 5 h. The reaction mixture was evaporated, and the resulting solid was purified by flash chromatography (silica gel, 0 to 10% MeOH in dichloromethane) to yield the title compound as a light yellow solid (7 mg, 49.6 %). MS ISP (m/e): 534.5 (100) [(M + H) + ] to yield N-(3-(4-(2-(8-fluoro-2-methylimidazo[1,2-a]pyridin-6-yl)-4-oxo-4H-pyrido[1,2-a]pyrimidin-7-yl)piperazin-1-yl)-3-oxopropyl)pentanamide (SMN-C7L).

### Chemical proteomics of splicing inducers

Coupling of the compound derivatives to the NHS-activated beads (GE Healthcare) was performed by incubation of 1 µmol of compound with 1 ml of bead solution. After binding overnight at room temperature in DMSO and 1.5% triethylamine, any remaining reactive groups were blocked with 33% (final volume) ethanolamine for 24 h at room temperature. Subsequent washing steps were performed with DMSO. Binding efficiency was estimated by comparing the optical densities of the compound solutions before and after coupling at the maximal absorbance wavelength 292 nm^35^.

Heavy and light SILAC labeled SMA type 1 fibroblasts were lysed in 50 mM HEPES pH 7.5 buffer containing 150 mM NaCl, 1 mM CaCl2, 1 mM MgCl2, 0.5% NP-40, protease inhibitors (Roche), sonicated using a Branson Digital Sonifier on ice. Samples were then cleared by centrifugation at 12,000 × *g* for 10 min at 4 °C. Heavy or light supernatants were loaded in quintuples onto Sepharose® beads immobilized with the active or inactive derivative for 1 h at 4 °C under rotation. Beads were washed three times with lysis buffer, treated with SDS-PAGE sample buffer for 5 min at 85 °C, and identical volume of both elutions (active and inactive beads) were combined. For the experiment using RNAse, an additional step was added before elution, using RNAse A (Roche) at 1.5 U μl^−1^ for 20 min at room temperature. Eluates were separated on 4–20% Tris-Glycine SDS-PAGE. After protein fixation in 40% ethanol and 10% acetic acid, gels were stained overnight with Coomassie Brilliant blue. Each gel lane was cut into seven slices between the 20 and 150 kDa region, in-gel digested as described above, and stored at −20 °C prior to analysis. Samples were re-constituted in 2% acetonitrile/5% formic acid and analyzed by liquid chromatography–mass spectrometry (LC–MS) using an Easy-nLC coupled to an LTQ Orbitrap Velos Pro (Thermo Fisher Scientific) equipped with a Digital PicoView 550 source (New Objective, Woburn, MA) and an active background ion reduction device (ABIRD, ESI Source Solutions, Woburn, MA). Peptides were loaded onto an Aqua C18 (Phenomenex, Torrance, CA) trapping column (packed in-house, 10 mm length × 100 μm inner diameter, 5 μm particle size) and separated on a Reprosil-Pur C18-AQ (Dr. Maisch GmbH, Ammerbuch, Germany) analytical column (200 mm length × 75 μm inner diameter, 3 μm particle size, 120 Å) using the following gradient at 250 nl per min: 0–2% B in 2 min, 2–35% B in 46 min, 35–80% in 2 min, and 80% B for 10 min (buffer A: 0.6% acetic acid; buffer B: 0.6% acetic acid/acetonitrile). The eluent was electrosprayed via uncoated SilicaTip emitters by applying 1.7-2-0 kV spray voltage via a liquid junction. The mass spectrometer was operated in the data dependent mode to automatically switch between MS and MS/MS. Survey full scan MS spectra were acquired from *m*/*z* 350 to 1700 in the Orbitrap with a resolution of *R* = 60,000 (at *m*/*z* 400) after accumulation to a target value of 1 × 106 in the linear ion trap for a maximum time of 500 ms. The detected ions were recalibrated on-the-fly using the ambient air polysiloxane at *m*/*z* 445.120024 as lock-mass *(51)*. The 10 most intense ions with a charge state > 1 + and a threshold above 1000 were selected for collision induced dissociation (CID) in the linear ion trap. Ions were accumulated to a target value of 10,000 using the predictive ion trap fill time mode, isolated with a width of 2 amu, CID performed at a normalized collision energy of 35% with an activation Q of 0.25 for 10 ms and wideband activation mode enabled. Fragmented ions with a *m*/*z* width of ±10 ppm width were placed on an exclusion list for 30 s and a maximal size of 500. Raw files were processed using Mascot Distiller 2.5.1.0 (Matrix Science) and peak lists were searched against the human UniProt/SwissProt database (December 2013 release, 20,274 sequences) using Mascot server. Trypsin/P was used as an enzyme, allowing for a maximum of one miscleavage, 20 ppm and 0.5 Da as precursor and fragment mass tolerances, respectively. Carbamidomethylated cysteine was selected as fixed, while oxidized methionine, was set as variable modifications. SILAC variable modifications Lys8 and Arg10 (heavy) were set in the SILAC quantification method. Individual peptide to spectrum matches were filtered using maximum expect *p*-value corresponding to a false discovery rate of ≤1% as determined by enabling the decoy database search. SILAC peptide quantitation and ratio generation was performed with Mascot Distiller using the following settings: precursor ion protocol, Simpson’s rule as integration method, minimal Rho correlation of 0.7, and an XIC threshold of 0.1. Ratios median was calculated for protein with a minimum of two quantified peptides. Peptide level protein quantitation reports were generated in html format for further processing. HTML reports were loaded into R 3.1.0 for statistical analysis, and only protein hits with at least two peptides, a Mascot Score > 31, found in at least 7 out of 10 samples and were used in subsequent analysis. The SDS band where a given protein was identified with highest abundance was kept, and protein entries in the other bands were discarded. Ratios of reversed peptides ratios were reversed, and all ratios were log2-transformed. One-sided *t* tests were performed, with the null hypothesis that the mean of the log-transformed ratios of a given protein equals 0. The corresponding alternative hypothesis states that the original, not-transformed ratios H:L (after H:L reversion, where appropriate) are significantly greater than 1:1. The *p*-values of the one-sided *t*-test were adjusted for multiple testing following Benjamini and Hochberg^36^. Proteins were considered as significantly and relevantly enriched if both the adjusted *p*-value ≤ 5 % and the ratio between groups > 1:1.5 were found.

From pull-down experiments with active and inactive compound, proteins were selected that showed at least 1.5 fold enrichment for the active compound, with an adjusted *p*-value of 0.05 or less. This produced a list of 430 proteins. Using the GO_SLIM collection of gene ontology annotations (http://ceur-ws.org/Vol-1546/paper_44.pdf), these proteins were found to be enriched for 8 categories based on Fisher’s exact test and using a *p*-value cutoff of 0.001. Each of them is characterized by the negative logarithm of the enrichment *p*-value on the *x* axis of Supplementary Fig. 4A; the full is given in Supplementary Table 3. The category “translation” scored best, but is not related to the compound’s mode of action. Reasoning that proteins involved in the translational machinery would be associated through their interactions with RNA, we produced a second list of 339 proteins by manually removing all ribosomal proteins from the original list. Repeating the enrichment analysis, we obtained drastically smaller or at best very similar enrichment *p*-values (*y* axis in Supplementary Fig. 4A), with the exception of the “RNA splicing” category, which scored substantially better. We conclude that this category is the strongest hit in our search that is independent of the (inevitable) presence of ribosomal proteins in our data set.

### RNA pulldowns with proteomics

100 pmol of 5 biotinylated ESE2 sequence (5′-AAAAAGAAGGAAGG-3′) was bound to 50 μl of streptavidin magnetic beads according to the manufacturer’s instructions (PierceTM Magnetic RNA–protein pull-down kit; Thermo Scientific; Rockford, USA). SMA type 1 human fibroblasts pellets were resuspended in 10 mM HEPES pH7.8, 1 mM MgCl2, 10 mM KCl, protease inhibitors (cOmplete™ Protease Inhibitor Cocktail EDTA free Roche) and homogenized with a potter. Lysates were centrifuged at 500 × *g* for 10 min at 4 °C. Pelleted nuclei were resuspended in 50 mM HEPES pH 7.3 buffer containing 150 mM NaCl, 1 mM CaCl2, 1 mM MgCl2, 0.5% NP-40, protease inhibitors, RNase inhibitors (Protector, Roche), and sonicated using a Q125 sonicator (Qsonica). Samples were then cleared by centrifugation at 300 × *g* for 10 min at 4 °C and the supernatant was incubated for 1 h 30 min at 4°C with bead-immobilized ESE2 sequence as bait. For competition experiments, lysates were pre-incubated for 1 h 30 min with increasing amounts of SMN-C6 or SMN-C7 (0; 0.5; 1; 5 and 10 μM final concentration) in triplicates. After washing 3 times in lysis buffer, proteins were eluted in reducing SDS-PAGE sample buffer for 5 min at 85°C. Eluates were separated on 4–20% Tris-Glycine SDS-PAGE and stained with Coomassie Brilliant blue. Gel lanes were cut in five bands spanning from 20 to 120 kDa. For protein in-gel digestion, an adapted protocol from Shevchenko et al.^37^ was used. Proteins were reduced with 50 mM dithiothreitol for 45 min at 56 °C, alkylated with 55 mM iodoacetamide for 1 h in the dark, and digested with trypsin (Promega) overnight at room temperature. Peptides were extracted twice with 1:2 (v/v) acetonitrile/25 mM ammonium bicarbonate, and 1:2 (v/v) acetonitrile/5% formic acid, respectively, for 15 min at 37 °C. Dried samples were re-constituted in 2% acetonitrile/5% formic acid and run in quintuples by LC–MS, using an EASY-nLC 1000 ultrahigh pressure liquid chromatography) coupled to an Orbitrap Fusion Mass spectrometer (Thermo Fisher Scientific, Bremen, Germany). Samples were concentrated on an Acclaim PepMap C18 trapping column (100 μm × 20 mm, 5 μm particle size). Peptides were separated on an Acclaim PepMap C18 EASY-spray column (75 μm × 500 mm, 2 μm particle size) using the following gradient at 300 nl per min: 7–50% B in 45 min, 50–80% B in 2 min, 80% B for 13 min, corresponding to a total time of 60 min (buffer A: 0.1% formic acid; buffer B: 0.1% formic acid/acetonitrile). Data were on-the-fly recalibrated using ambient air hexacyclodimethylsiloxane at *m*/*z* 445.12002^38^. The ten most intense precursor ions, with charge states between 2 and 6, a minimum intensity of 5E3, were mono-isotopically selected for higher-energy collisional dissociation, using a quadrupole isolation of *m*/*z* 0.7, automatic gain control target of 1E4, maximum IT of 35 ms, collision energy of 30%, and ion trap readout with rapid scan rate. Only a single charge state per precursor was selected for MS2. Interrogated precursor ions were dynamically excluded for 75 s using a ±10 ppm mass tolerance. Raw files were processed using Progenesis QI 2.1 (Nonlinear Dynamics; Newcastle, UK) and Mascot Server 2.5.1 (Matrix Science, London, UK) together with the UniProt/SwissProt human protein database (08.2015 release, 20,204 entries without splice variants) using trypsin/P as an enzyme, a maximum of two missed cleavage sites, 10 ppm, and 0.5 Da as the precursor, and fragment ion tolerances, respectively. Carbamidomethylated cysteines (+57.02146 Da) were set as static while oxidized methionines (+15.99492 Da) were set as dynamic modifications. For statistical analysis of the raw data and in order to detect proteins displaced with increasing free SMN-C6 and SMN-C7 concentration, the concepts of contrast tests^39, 40^ combined with a moderated linear model^41, 42^ were applied^35^. Monotonic contrasts were used to compare the protein abundance values above and below each concentration point, and the maximum of the resulting series of *t*-statistic values was determined for each protein quantitation group. To obtain the *p*-values, the concentration labels were permuted 1000 times based on the step-down minP algorithm^43, 44^ modified for one-sided tests, and adjusted for multiple testing^36^. Proteins with adjusted *p*-values below 5% were considered as specific binders. Computations were performed in R (version 3.3.2; http://www.r-project.org/).

### PCR after compound pull-downs

After incubation with cell lysate and washing of Sepharose beads immobilized with the active or inactive SMN-C5 analogs, RNA was released from the beads by adding QIAzol reagent and isolated using the miRNeasy Mini kit combined with DNase treatment on a solid support (Qiagen Inc., USA). Equal volumes of both elutions (active and inactive beads) were used for cDNA synthesis in separate reactions at 50 °C using the Transcriptor first strand cDNA synthesis kit with random hexamer and oligo-dT primer (Roche). Real-time quantitative PCR was performed on a Roche LightCycler LC480 instrument using a fixed volume of cDNA as input in a 20 µl mixture containing LightCycler 480 Probes Master (Roche), 0.4 µM forward and reverse primers, and 0.2 µM FAM labeled hydrolysis probe. The temperature cycle consisted of pre-incubation at 95 °C for 10 min and 50 cycles of amplification (95 °C for 10 s, 60 °C for 30 s, 72 °C for 2 s). Fold enrichment of active above inactive derivative was calculated with the 2-∆∆Ct method. The primer and probes used for RT-qPCR are described in Supplementary Table 4.

### RT-qPCR analysis of SMN2 full length and Δ7 mRNAs

SMA type 1 patient cells were plated at 7000 cells per well in 200 μl Dulbecco’s modified Eagle’s medium (DMEM) with GlutaMAX and 10% fetal bovine serum (FBS) (Life Technologies, Inc.) in 96-well plates. After the cells were adhering, the treatment started with the SMN-C3 at different concentrations (0.5% DMSO) and with different inhibitors (Supplementary Table 4) in triplicates for 6 h in a cell culture incubator (37°C, 5% CO2, 100% relative humidity). After removal of the supernatant, cells were lysed in Cells-To-Ct lysis buffer (Life Technologies, Inc.) according to the manufacturer’s recommendations.

The mRNA levels of *SMN2* FL, *SMN2* Δ7, *STRN3* FL, *STRN3* Δ8/9, and *GAPDH* were quantified using Taqman-based RT-qPCR and *SMN2*-specific primers and probes in Supplementary Table 5 (purchased from Life Technologies, Inc. or Microsynth. Inc.). The *SMN2* forward and reverse primers were each used at a final concentration of 0.4 μM. The *SMN2* probe was used at a final concentration of 0.15 μM. *GAPDH* primers were used at final concentrations of 0.1 μM and the probe at 0.075 μM. RT-qPCR was carried out at the following temperatures for indicated times: Step 1: 48 °C (15 min); Step 2: 95 °C (10 min); Step 3: 95 °C (15 sec); Step 4: 60 °C (1 min); Steps 3 and 4 were repeated for 40 cycles. The Ct values for each mRNA were converted to mRNA abundance using actual PCR efficiencies. *SMN2* FL and Δ7 mRNAs were normalized to *GAPDH* and DMSO controls and plotted as fold change compared to DMSO treatment.

### Bioinformatic analysis of RNA-sequencing data

To estimate gene expression levels, paired-end RNASeq reads were mapped onto the human genome (hg19) by using the short read aligner GSNAP^45, 46^ and RefSeq transcript annotations. Mapped reads for all transcript variants of a gene (counts) were combined into a single value, normalized and denoted as rpkms (number of mapped reads per kilobase transcript per million sequenced reads)^47^. Differentially expressed genes were determined by using the DESeq^48^ package in R/Bioconductor with number of mapped reads per gene as input. For the splice-site analysis (see below), paired-end RNASeq reads were mapped onto RefSeq human transcripts using the alignment software GSNAP with default parameters and the option “sam-multiple-primaries” in order to account for reads that map multiple times to different splice variants. The number of reads spanning splice sites was determined by applying the samtools mpileup software^49^ with default parameters for each transcript. Reads were excluded from the analysis if their start or end coordinate mapped exactly onto the splice-site.

### RNAseq alternative splicing analysis

The sequencing reads generated do not allow unambiguous identification of full-length transcript variants. Therefore, we generated a database of local alternative splicing events based on the human RefSeq database of transcripts (Release 66, August 2014). Such local splicing events we classified into the following categories:

CASSETTE—inclusion or skipping of a single exon

INTERNAL3—use of an alternative 3′ acceptor splice site, leading to a length polymorphism

INTERNAL5—use of an alternative 5′ donor splice site, leading to a length polymorphism

LCASSETTE—joint inclusion or skipping of two exons

MUT_EXCLU—mutually exclusive usage of one out of two exons

XCASSETTE—joint inclusion or skipping of three or more exons. For each local splicing event, we distinguish between two alternatives, e.g., including or skipping the exon in the case of a CASSETTE event, and we collect all the sequencing reads that are unique for either alternative; for the CASSETTE case, these are reads covering the different splice junctions or mapping fully to the alternative exon. We normalize the obtained read counts for either alternative by the length of the unique sequence stretch (which is typically longer for the exon inclusion alternative) to obtain normalized counts c1 and c2 for the two alternatives, respectively.

Splicing events are then characterized by a PSI (“percent spliced in”) score PSI = *c*1/(*c*1 + *c*2), which ranges from 0 to 1. Changes were determined by ∆PSI values (relative to matched controls). 57,634 local splicing events were represented by normalized read densities of at least 0.003 for at least one variant. For all of these, we calculated ∆PSI values. In total, 42 transcriptome-wide splicing events were observed with a ∆PSI of >0.4 for at least one of the treatment conditions.

### SPR direct binding assay

All SPR experiments were performed on the Biacore® 3000 and 2000 (GE Healthcare, Uppsala, Sweden) instruments at 25 °C. Running buffer composed of 10 mM HEPES, 150 mM NaCl 0.05% P20 (w/v), 35 mM EDTA, 0.1% (v/v) DMSO, pH 7.4 and flow rate of 30 µl min^−1^ were used by direct binding assays with small molecule compounds. Running buffer composed of 20 mM NaH_2_PO_4_, 150 mM NaCl, pH 5.5 and flow rate of 50 µl min^−1^ were used in binding assay with hnRNP G and ESE2, and further competition assay with small molecule compounds. Running buffers were prepared freshly, filtered with Express™Plus steritop filters with 0.22 µm cut off (Millipore, Billerica, MA, USA) and degassed prior the SPR analysis. 5′- biotinylated RNA Sequences were obtained from Microsynth AG (Balgach) and were dissolved in RNase-free water to concentrations of 100 µM. The RNA aliquots were stored at −20 °C and used only once. Biotinylated RNAs were captured via biotin on streptavidin pre-coated SA sensors (GE Healthcare BR-1000-32) or CAP sensor (GE Healthcare, 28-9202-33). SA streptavidin sensor was conditioned first with 3 consecutive 1 min injections of high salt solution in sodium hydroxide (50 mM NaOH, 1 M NaCl). Next, RNAs were diluted in running buffer 1000 times from stock solution (100 µM) and applied over streptavidin sensor surface to achieve immobilization levels of RNA of about 1000 RU. Finally, free biotin solution (10 µM in running buffer) was injected once (1 × 1 min) over sensor surface to block remaining binding sites in streptavidin. CAP sensor was used to monitor hnRNP G RRM peptide - ESE2 interactions and in competition assays. First, hybridization of DNA-streptavidin conjugate on the pre-coated complementary ssDNA was performed according standard protocol. Next, biotinylated ESE2 was immobilized to relatively low level of ~20 RUs. Finally, saturation of free binding sites in streptavidin was performed with a biotin solution similarly as on SA sensor. After titration of hnRNP G over ESE2 surface the entire DNA-streptavidin-ESE2-hnRNP G complex was washed away from the sensor using regeneration solution of 6 M guanidine hydrochloride in 0.25 M NaOH and further the new hybridization of DNA-streptavidin conjugate on the CAP sensor, immobilization of biotinylated ESE2 and finally hnRNP G titration experiment were performed.

For binding experiments of small molecule ligands to various RNA regions, SMN2 splicing modifier compounds were obtained from Roche (Basel, Switzerland) as powders, dissolved in 100% DMSO to a stock concentration of 5 or 10 mM. For the measurements, the samples were heated 5 min to 65 °C and finally cooled to the room temperature. Binding experiments were performed under the same conditions as immobilization of RNAs. Binding of small molecule ligands to various RNA regions (Supplementary Table 6) was analyzed at a single concentration (10 µM) in five replicates.

To assess binding of hnRNP G RRM peptide to ESE2 and competition by small molecules, hnRNP G RRM was diluted in the running buffer (described above) and titrated (0.094–1.5 µM, dilution factor of 2) over ESE2 surface in quadruplicates. For the competition experiments hnRNP G RRM at a single concentration (1.5 µM) was pre-incubated with a concentration range of small molecule (3.13–50 µM, dilution factor of 2) and applied over the ESE2 surface. Five consecutive injections of hnRNP G at 1.5 µM were performed as a control experiment.

SPR data analysis was performed using BiaEvaluation Software (version 4.1) and GraphPadPrism (version 6.04). All monitored resonance signals were single referenced, i.e., signals monitored on the binding active channel were subtracted with signals from a reference channel (sensor surface not modified with any RNA, but saturated with free biotin). To compare binding of different molecules on various RNA regions, the resonance signals were normalized to molecular mass and RNA surface densities, and expressed as % binding.

### Cell culture and transfection

HEK293 cells (ATCC) were cultured in DMEM (4.5 g per l glucose, l-glutamine and sodium pyruvate, Corning 10-013-CV) + 10% FBS. For transfection with plasmid DNA, the cells were plated in 24-well plates the day prior to transfection at 5 × 10^5^ cells per well. Transfection of 0.25 µg plasmid DNA per well was carried out using Lipofectamine 2000 (Life Technologies, 11668-019) according to the manufacturer’s instructions. 24 h post-transfection, cells were treated with 0–10 µM of splicing modifier for 24 h. Cells were lysed using 300 µl TRIzol reagent (Life Technologies, 15596018) per well.

### RNA extraction

To each 300 µl TRIzol sample, 80 µl chloroform were added and mixed vigorously. The samples were centrifuged 15 min to separate the phases. The upper aqueous phase was transferred to a new tube and mixed with one volume of isopropanol. After incubation for 10 min at room temperature, the RNA was pelleted by centrifugation for 15 min at 4 °C. The pellet was washed with 75% ethanol and air-dried for 5–10 min at room temperature. After resuspension in 20 µl RNase-free water, 2.2 µl 10× RQI DNase buffer (Promega, M198A) and 1 µl RQI DNase (Promega, M610A) were added and the samples were incubated at 37 °C for > 30 min. After addition of 2.4 µl RQI stop solution (Promega, M199A) the samples were incubated for 2 min at room temperature, spun down for 2 min, and transferred to a fresh tube. Samples were stored at −20 °C short term.

### cDNA synthesis

A total of 400 ng RNA were mixed with 1 µl oligo-dT primer (18-mer, 0.5 µg µl^−1^) and diluted with RNase-free water 5 µl total volume. The samples were incubated at 75 °C for 5 min and put on ice. In a separate tube, 6.6 µl nuclease-free water were mixed with 4 µl ImProm-II 5× reaction buffer (Promega, M289C), 2.4 µl MgCl_2_ (25 mM), 1 µl dNTP mix (10 mM), and 1 µl ImProm-II reverse transcriptase (Promega, M314C). This solution was added to the RNA/primer sample and the reaction mix was incubated 5 min at room temperature, 1 h at 42 °C, and 15 min at 70 °C. The samples were centrifuged for 2 min and transferred to a fresh tube. Samples were stored at −20 °C.

### Radioactive PCR and gel analysis

Three µl of DNA loading dye were added to each reaction, then 3 µl were loaded on a 6% acrylamide gel (2 ml TBE 10×, 4 ml Bis/Tris Acrylamide solution 30%, 14 ml water, 180 µl APS 10%, 18 µl TEMED). Gel electrophoresis was carried out in 1× TBE buffer at 400 V for 1.5 h. The gel was dried for 25 min at 88 °C under vacuum, and analyzed by autoradiography.

### In vitro transcription

To prepare the template for the in vitro transcription, the pCI-neo plasmid containing a T7 promoter upstream of the gene of interest was linearized using SalI. After gel purification, the in vitro transcription reaction mix was prepared as follows: 9.4 µl of this mix were combined with 2.1 µl of the linearized DNA template and 1 µl T7 RNA polymerase (1.36 mg ml^−1^) in in vitro transcription buffer (Supplementary Table 7). The reactions were incubated for 90 min at 37 °C. After incubation, 10 µl of loading dye (100% formamide + bromophenol blue and Xylene Cyanole FF (Kodak) + EDTA (20 µl 0.5 M EDTA pH 8.0/1 ml formamide)) were added to each 12.5 µl reaction. The entire reaction was loaded onto a 9% Urea recovery gel (7.2 ml Urea stock 25% + 2 ml buffer + 10.8 ml system diluent + 250 µl APS 10% + 10 µl TEMED). Electrophoresis was carried out in 1× TBE at 500 V for 50 min followed by 650 V for 1 h and 50 min. The bands were excised and the RNA was extracted by incubating the gel piece in 300 µl of elution buffer (0.3 M NaAcetate, 0.2% SDS) overnight at 4 °C.

300 µl Tris-saturated phenol were added to the liquid. The samples were centrifuged for 10 min at 4 °C. The top aqueous layer was transferred to a fresh tube and 1 ml of 100% EtOH were added to precipitate the RNA. After incubation at −80 °C for > 30 min the samples were centrifuged for 30 min at 4 °C. The pellet was air-dried for 5 min at room temperature and resuspended in 10 µl nuclease-free H_2_O. The RNA was quantified by scintillation measurement using a Beckman LS 6000SC scintillator. The RNA was stored at −20 °C until further use.

### In vitro splicing assay

Ten fmol of ^32^P-labeled, m7G-capped T7 transcripts were spliced in a 20-µl reaction as described^50^. Splicing reactions contained 1.56 mM MgCl_2_, 32 mM HEPES, 0.5 mM ATP and 20 mM creatine phosphate, 1.3% PVA, 40% HeLa nuclear extract, and 1× buffer D (20 mM HEPES-KOH pH 8, 25% glycerol, 1.5 mM MgCl_2_, 0.2 mM PMSF, 0.5 mM DTT, 0.6 M KCl). After incubation for 3 h at 30 °C, reactions were quenched with 10 volumes of splicing stop solution (100 mM Tris pH 7.5, 10 mM EDTA, 1% SDS, 150 mM NaCl, 300 mM NaAc). The RNA was extracted using phenol, ethanol precipitated, and analyzed by denaturing PAGE and autoradiography.

### RNA-sequencing

PNN 1–46 cells (Coriell) derived from a type 1 SMA patient were treated with SMN-C3 at 500 nM and with NVS-SM1 at 24 nM for 24 h. RNA was extracted with the RNeasy Mini Kit (Qiagen Inc., USA). Template DNA molecules suitable for sequencing were prepared from 400 ng of total RNA using the TruSeq Stranded mRNA Library Preparation Kit (Illumina Inc., San Diego, CA) according to manufacturer’s instructions. After 14 cycles of PCR amplification, the size distribution of the barcoded DNA libraries was estimated by electrophoresis on Agilent High Sensitivity Bioanalyzer microfluidic chips. Minimum sizes of amplified libraries were determined as > 200 nucleotides and average sizes of ~300 nucleotides. Libraries were quantified using the KAPA Library Quantification Kit (Kapa Biosystems, Boston, MA). Libraries were pooled at equimolar concentrations, spiked with 1% PhiX control library, and diluted to 20 pM prior to loading onto the flow cell of an Illumina HiSeq 2500 instrument (Illumina Inc., San Diego, CA) for both clustering and sequencing. Libraries were extended and bridge amplified to create single sequence clusters using the HiSeq PE Cluster Kit v4 cBot HS (Illumina Inc., San Diego, CA). The flow cell carrying amplified clusters was then sequenced in high output run mode with 51 cycles for read 1, 7 cycles for the barcode index and 51 cycles for read 2 using the HiSeq SBS Kit v4 chemistry (Illumina Inc., San Diego, CA). 2 × 50-bp paired-end reads were generated with ~53 million read pairs per sample. Real-time image analysis and base calling was performed on the HiSeq 2500 with the HiSeq Control Software v2.2.37. CASAVA software version 1.8.2 was used for demultiplexing and production of FASTQ sequence files.

The RNA-sequencing data from this study has been deposited at the NCBI Gene Expression Omnibus (http://www.ncbi.nlm.nih.gov/geo) under the accession number GSE86001.

### NMR spectroscopy

NMR spectroscopy measurements were performed on a Bruker AV-III 500 MHz (Bruker) and AV-III HD 900 MHz (Bruker) both equipped with cryo-probes. RNA oligonucleotides were purchased (Microsynth AG), resuspended in water and precipitated with butanol to remove traces of small molecules resulting from the chemical synthesis. In order to form the duplex between the 5′-end of U1 (5′-AUACψψACCUG-3′) and the 5′-splice site of *SMN2* (5′-GGAGUAAGUCU-3′), both oligonucleotides were mixed in equimolar amounts in the NMR buffer (MES 10 mM pH 5.5, 50 mM NaCl, 10% D20) at final concentration of 400 µM, annealed to 90 °C and cooled down to room temperature. The RNA duplex resonances were assigned by using 2D ^1^H–^1^H homonuclear and natural abundance 2D ^1^H–^13^C HSQC experiments recorded at 283 K. After assignment of the RNA resonances, distance constraints were derived from the analysis of the NOESY spectra and used to model the RNA duplex using a simulated annealing procedure. The chemical shifts and the coordinates of the atomic models have been deposited into the BMRB and PDB under the following accession numbers 34171 and 5OR0, respectively. The oligonucleotide corresponding to ESE2 (5′-AAAAAGAAGGAAGG-3′) was also dissolved in the NMR buffer at the same concentration. The three molecules, SMN-C5, SMN-C7, and NVS-SM1 were resuspended in the NMR buffer at a final concentration of 4 mM. To monitor the interaction between the RNA duplex and the three molecules, NMR titrations were performed at 298 K by adding the small molecule into the RNA sample. The RNA resonances were monitored by recording 1D ^1^H and ^2^D ^1^H–^1^H TOCSY spectra for the following RNA:ligand molar ratios 1:0, 1:0.5, 1:1, 1:1.5, 1:2, 1:3, 1:4, and 1:5. In addition, natural abundance 2D ^1^H–^13^C HSQC was recorded for the free form of the RNA and after addition of one molar equivalent of SMN-C5. To monitor the interaction between ESE2 and the three small molecules, NMR titrations were performed at 310 K in order to prevent oligomerization of the RNA. RNA resonances were monitored by recording 1D^1^H spectra for the following RNA:ligand molar ratios 1:0, 1:0.5, 1:1, 1:1.5, 1:2, 1:3, 1:4, and 1:5. The U1-C zinc finger (1–61) was expressed in fusion with a GST tag and purified by affinity chromatography. After cleavage of the GST tag, U1-C zinc finger was further purified by size exclusion chromatography in HEPES 10 mM pH 7.2, 100 mM NaCl, 10 mM ZnSO_4_, and DTT 5 mM. U1-C was then complexed to the RNA duplex U1-5′ss and further titrated by SMN-C5. NMR titrations were performed at 298 K by adding the small molecule into the protein–RNA sample. Protein–RNA complexes of Tra2-β1 and hnRNP G RRMs bound to the RNA molecules 5′-AAGAAC-3′ and 5′-GACAAA-3′ (Microsynth AG) were formed at a ratio of 1:1 by adding the RNA into the protein sample in their NMR buffer (50 mM l-Glu, 50 mM l-Arg, 0.05% β-mercaptoethanol, 20 mM NaHPO4, pH 5.5), respectively. 2D^1^H–^15^N HSQC spectra were then recorded in the absence and in the presence of molecules at a protein–RNA complex:molecule ratio of 1:5. All NMR spectra were recorded at 313 K.

### Data availability

All relevant data are available from the authors upon request. The RNAseq data are published and available at the NCBI Gene Expression Omnibus (GEO) with accession number GSE86001. The chemical shifts and the coordinates of the atomic models from the NMR data are published and available in BMRB with BMRB entry number 34171 and PDB with PDB ID 5OR0.

## Electronic supplementary material

Supplementary Information
