# Supplementary material for: Binding to SMN2 pre-mRNA-protein complex elicits specificity for small molecule splicing modifiers
Source: Nat Commun. 2017 Nov 14;8:1476. doi: 10.1038/s41467-017-01559-4 (PMC5684323; doi:10.1038/s41467-017-01559-4)
Supplement: Supplementary file 1 — Supplementary Information [file 41467_2017_1559_MOESM1_ESM.pdf]

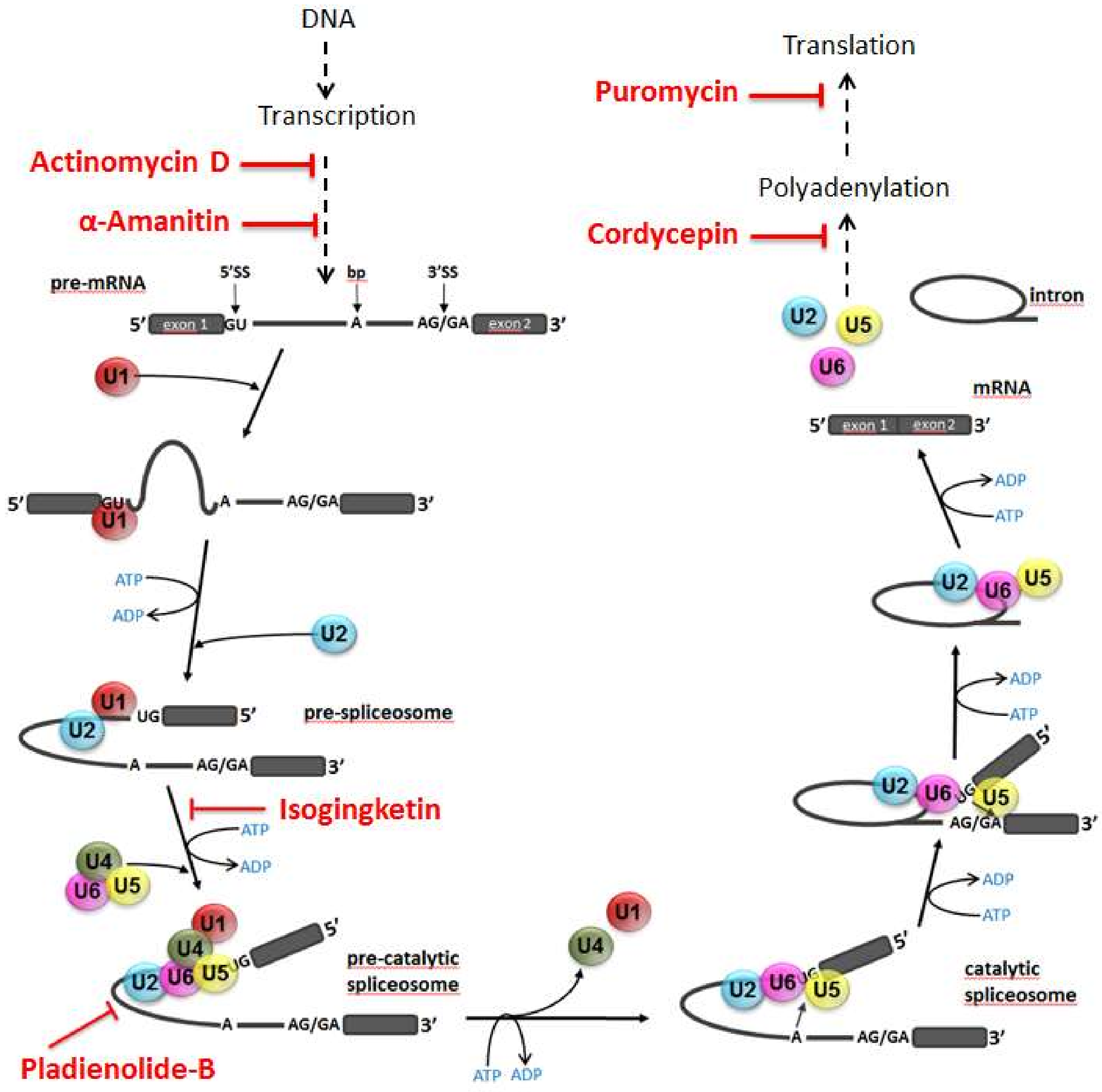


Supplementary Figure 1. Schematic diagram of the transcriptional process, including assembly process of the spliceosome, and inhibitor compounds that were used to block the respective steps (data in Fig. 1B, 1C, 1D and Supplementary Figure 2).

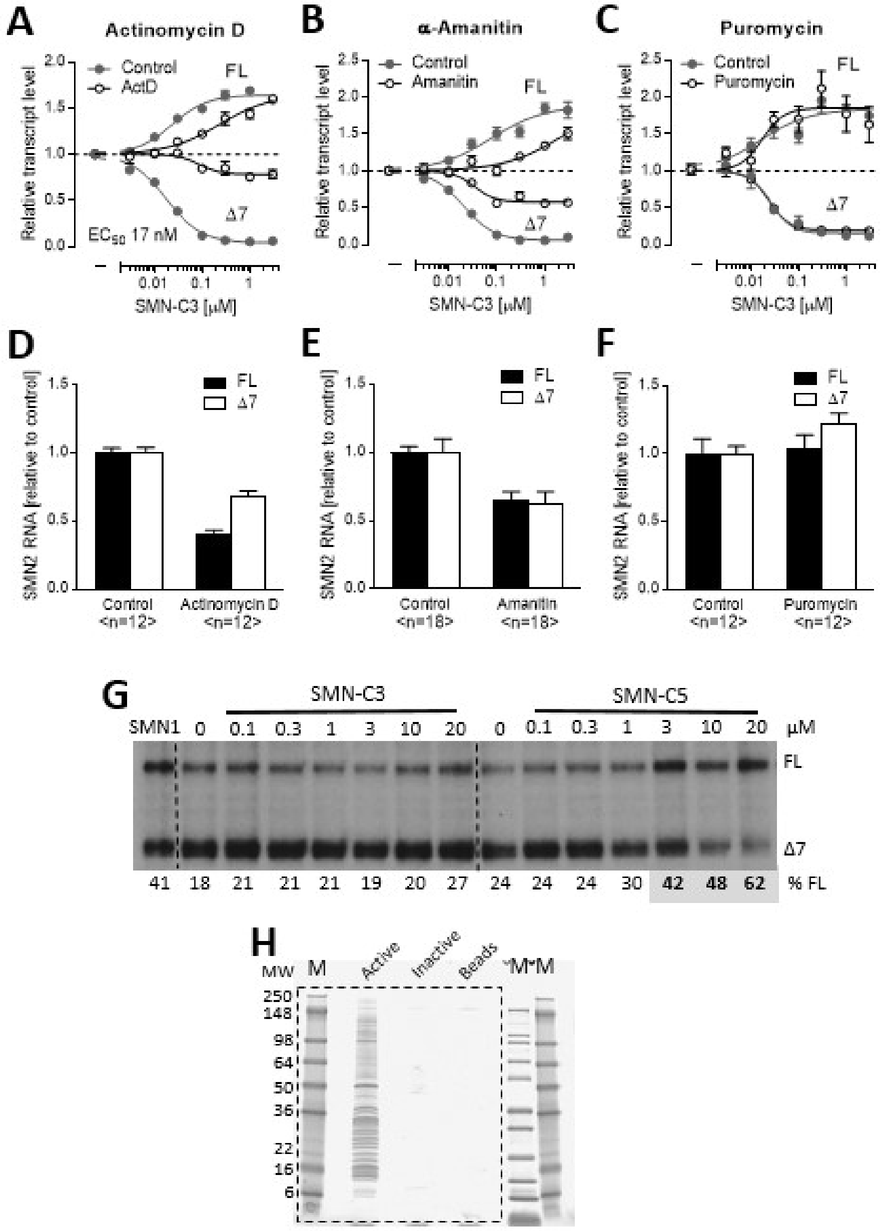


**Supplementary Figure 2.** Compound effect on transcription, translation and RNA splicing

RT-PCR analysis of *SMN2* mRNA FL and Δ7 relative transcripts levels in SMA Type I patient fibroblasts treated for 6 hours with inhibitors acting at various steps of transcription or translation (**A**) actinomycin D (1 μg/ml), (**B**) α-amanitin (50 μg/ml), and (**C**) puromycin (10 μg/ml), and simultaneously treated with the *SMN2* splicing modifier SMN-C3. (**D**, **E**, **F**) Baseline amounts of SMN2 FL or Δ7 mRNA after 6 hours treatment with (**D**) actinomycin D (1 μg/ml), (**E**) α-amanitin (50 μg/ml), and (**F**) puromycin (10 μg/ml). (**G**) In vitro splicing assay of SMN2-minigene transcript with cell-free nuclear extract, in the presence of SMN-C3 and SMN-C5 (0.1-20 μM). Splicing of SMN1 minigene transcript served as a control in lane 1. (**H**) Full commassie gel of Fig. 1D wish the dashed line illustrating the region shown in the main Figure. "M" indicated Marker. Data in (**A-F**) represent means ± SEM of 12-18 assessments per concentration in up to 6 independent experiments, data in (**G**) are representative of three independent experiments.

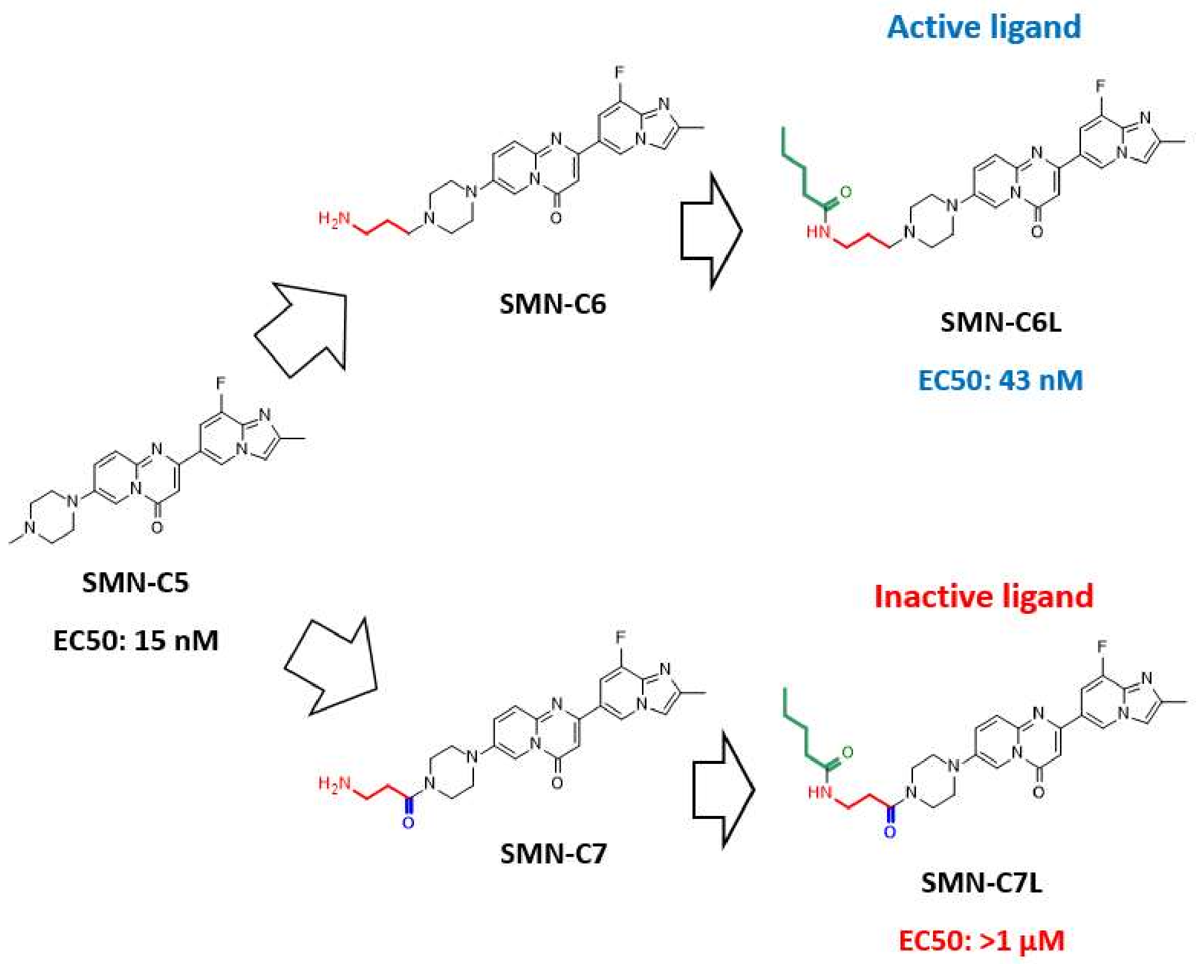


**Supplementary Figure 3.** Schematic representation of ligand generation starting with SMN-C5, a highly potent *SMN2* splicing modifier

By minimal modification, two ligands were created, SMN-C6 and SMN-C7. SMN-C6L and SMN-C7L were synthesized to mimic the immobilization of the later and were tested to verify potency in modifying *SMN2* splicing in a cellular assay using Type I patient fibroblasts (SMN-C6L, Active, EC50 43 nM; SMN-C7L, Inactive, EC50 >1 µM)

A

| Gene names | sn RNP | Active/Inactive (log 2 Fold change) |
|---|---|---|
| SNRPD1 | Sm protein | 3.92 |
| SNRPD2 | Sm protein | 3.13 |
| SNRPD3 | Sm protein | 3.96 |
| SNRPE | Sm protein | 3.92 |
| SNRPF | Sm protein | 3.90 |
| SNRPA | U1 | 3.70 |
| SNRNP70 | U1 | 3.70 |
| SRSF1 ASF SF2 | U1/U2 | 3.84 |
| U2AF2 U2AF65 | U2 | 2.80 |
| SF3B1 | U2 | 3.20 |
| SF3B3 | U2 | 2.29 |
| PTBP1 PTB | U2 | 3.92 |
| PRPF8 | U5 | 2.07 |
| EFTUD2 | U5 | 2.91 |
| DHX15 | U2; U5; U6 | 3.90 |
| NHP2L1 | U4/U6-U5 | 4.89 |
| SART3 | U6 | 2.63 |
| SRPK2 | U4/U6-U5 | 3.96 |
| NONO | U4/U6-U6/U1A | 2.98 |
| SFPQ PSF | U4/U6-U7/U1A | 3.49 |

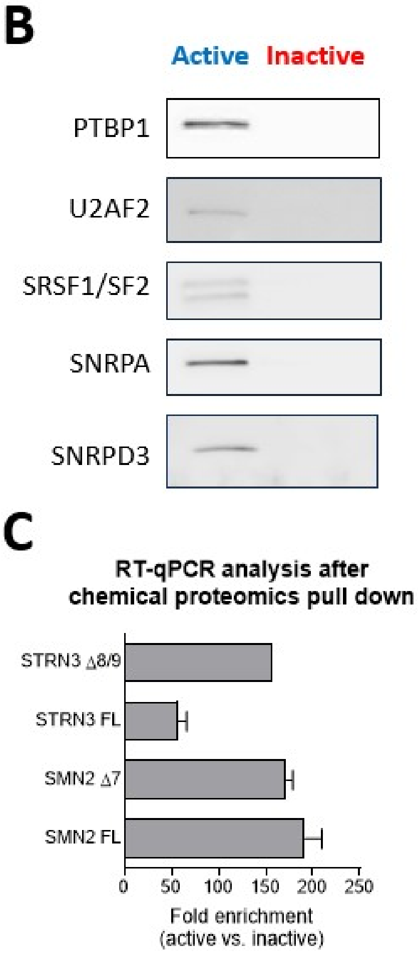


**Supplementary Figure 4.** Chemical proteomics using either splicing modifiers as ligands immobilized on sepharose beads

Whole protein extracts from differentially labeled SMA type 1 patient fibroblasts were loaded either on Active (SMN-C6) or Inactive (SMN-C7) affinity matrices. Elution fractions from both affinity chromatographies were combined, separated by one-dimensional gel electrophoresis, in gel digested and peptides extracted followed by LC-MS/MS analysis and quantification (**A**) Representation of some members of the snRNP family and levels of enrichment in the chemical proteomics pull-down with Active vs. Inactive compound (indicated by log 2-fold change). (**B**) Western blot analysis of the pull-down to Active vs. Inactive compound with antibodies against PTBP1, U2AF2, SRSF1/SF2, SNRPA and SNRPD3. (**C**) Fold enrichment (Active over Inactive compound) of *SMN2* and *STRN3* mRNA variants in eluates of the affinity matrices on immobilized active vs. inactive compound. Data in (**C**) represent means ±SEM of three independent samples.

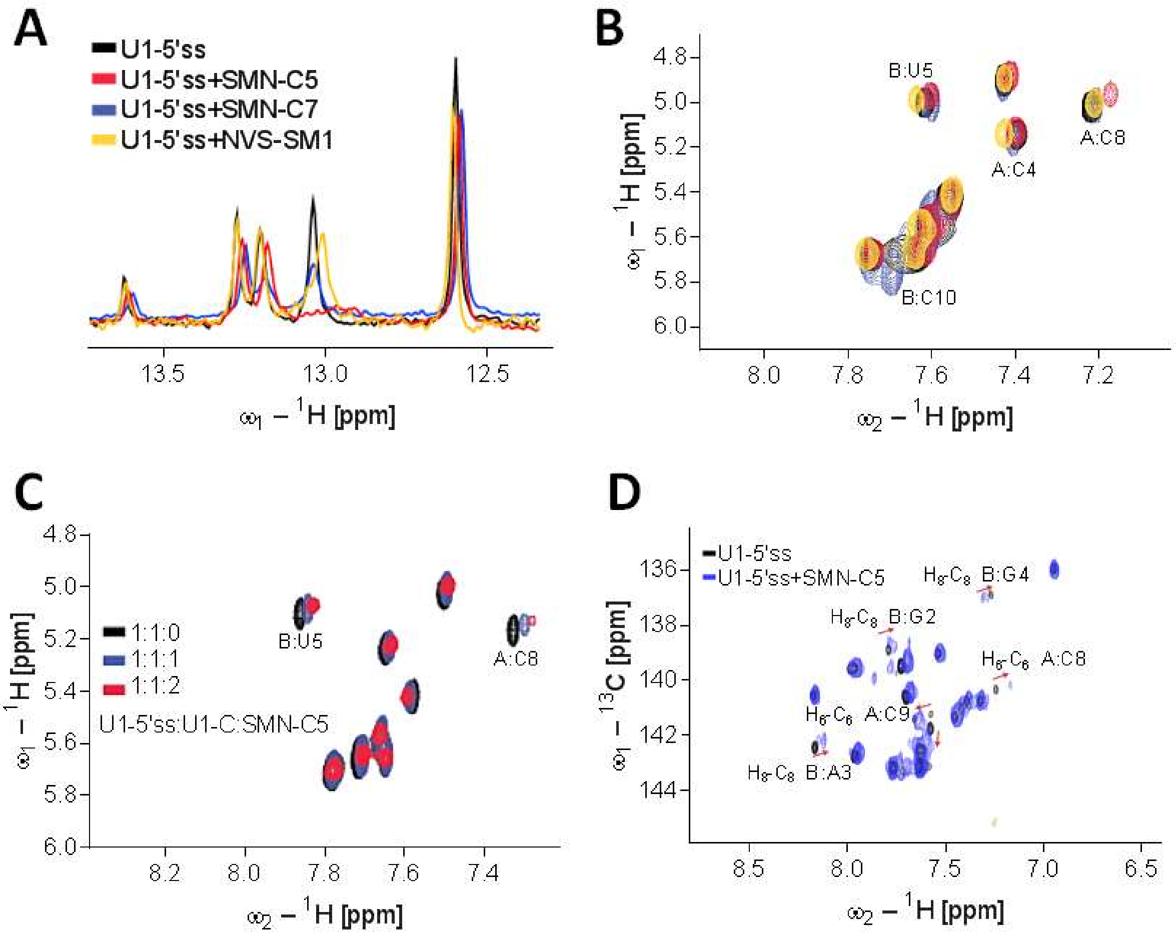


**Supplementary Figure 5.** NMR spectra of the binding of splicing modifiers to U1-5'ss RNA duplex

(**A**) Overlay of the 1D $^{1}$H NMR spectra showing the signal of the imino protons of the RNA duplex (U1-5'ss) upon addition of the three different compounds (SMN-C5/active, SMN-C7/inactive, and NVS-SM1). (**B**) Overlay of the 2D $^{1}$H-$^{1}$H TOCSY spectra showing the signal of the pyrimidine bases of the RNA duplex (U1-5'ss) upon addition of the three different compounds (SMN-C5/active, SMN-C7/inactive, and NVS-SM1). (**C**) NMR titration performed in presence of the zinc finger domain of U1-C supports SMN-C5 binding in the same location without affecting the binding of U1-C, both binding interfaces are located on opposite sites around the invariant GU motif of the 5'ss. (**D**) Overlay of the 2D $^{13}$C-$^{1}$H HSQC of the RNA duplex recorded before and after addition of SMN-C5. Protons that experienced large chemical shift changes are located in the major groove of the RNA duplex.

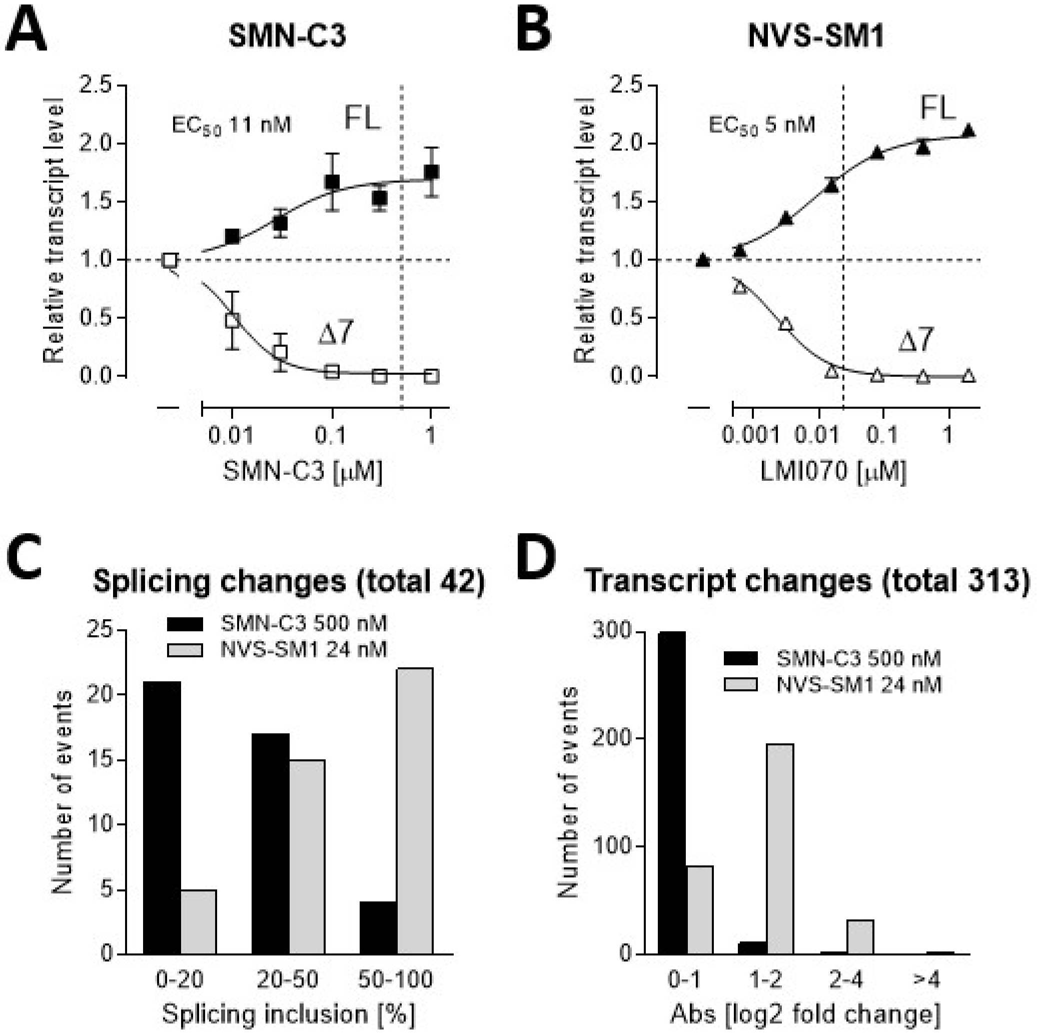


**Supplementary Figure 6.** Comparison of splicing correction, total number of splicing and transcript changes

RT-PCR analysis of *SMN2* full-length (FL) and exon 7 lacking (Δ7) mRNA in type 1 SMA patient fibroblasts treated with SMN-C3 (**A**) at 500 nM (~50x $EC_{50}$), and NVS-SM1 (**B**) at 24 nM (~5x $EC_{50}$, respectively), indicated by the dashed lines. (C) Histogram of transcriptome-wide splicing events (total 42) for all treatment conditions with a ΔPSI of >0.4 (SMN-C3 500 nM; NVS-SM1 24 nM), with binning at the 20% and 50% splicing correction level. (**D**) Histogram of transcriptional profiling changes (total 437) at $p<0.001$ in any of the treatment conditions, with binning at the 1, 2 and 4 fold change level. For SMN-C3, 95% of the log fold changes were <1.

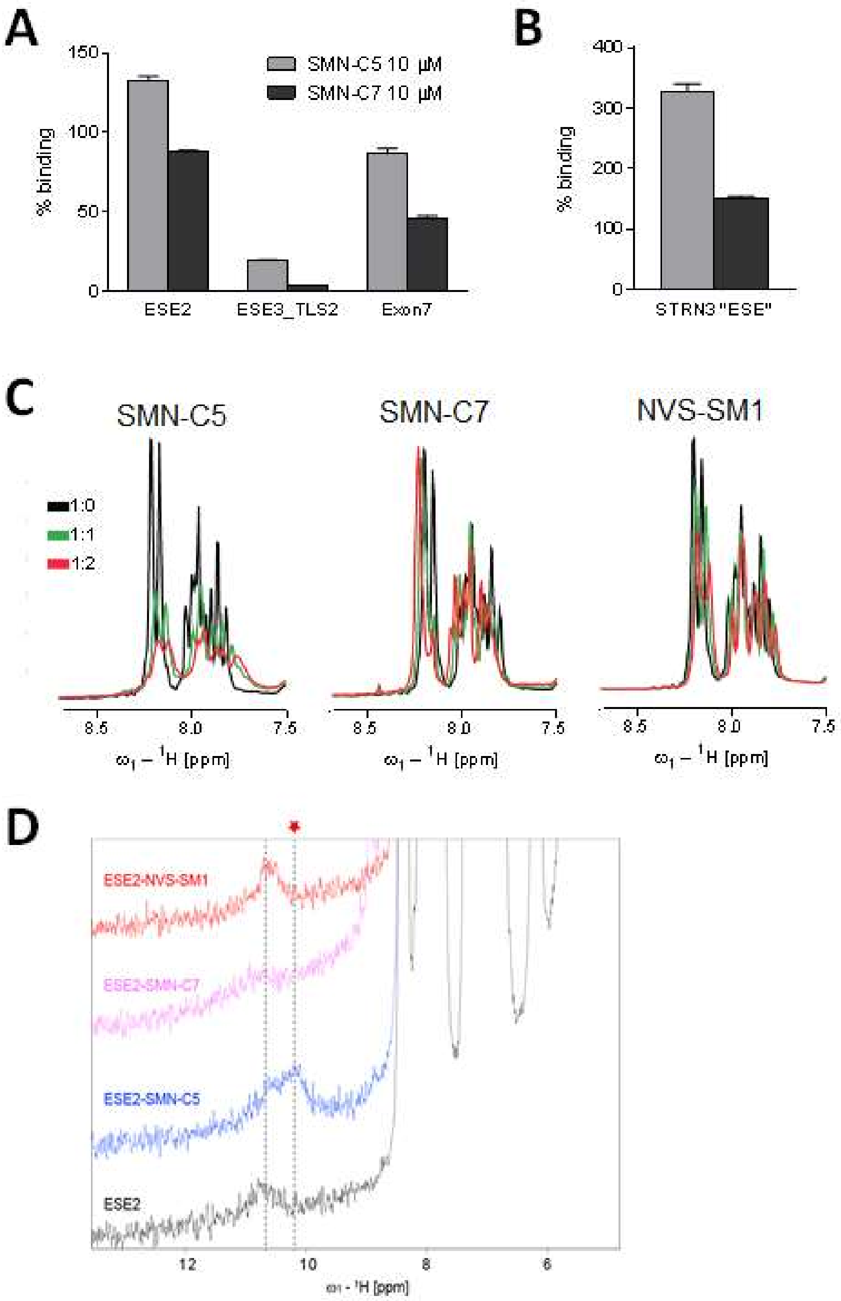


**Supplementary Figure 7.** Binding of the splicing modifiers to RNA measured by SPR and solution NMR

SPR (Biacore) binding at a single concentration 10µM with the active compound, SMN-C5, and the inactive compound SMN-C7 to ESE2 in *SMN2* exon 7 (**A**) or to the putative ESE in *STRN3* exon 8 (**B**). (**C**) Binding of SMN-C5, SMN-C7 and NVS-SM1 to *SMN2* ESE2 RNA by means of solution NMR. **(D)** Addition of SMN-C5 but not SMN-C7 or NVS-SM1 induced the formation of new broad imino signals (asterisk) in the proton 1D spectrum of the complex ESE2-SMN-C5, Data in (**A**) and (**B**) represent means ± SEM of three replicates.

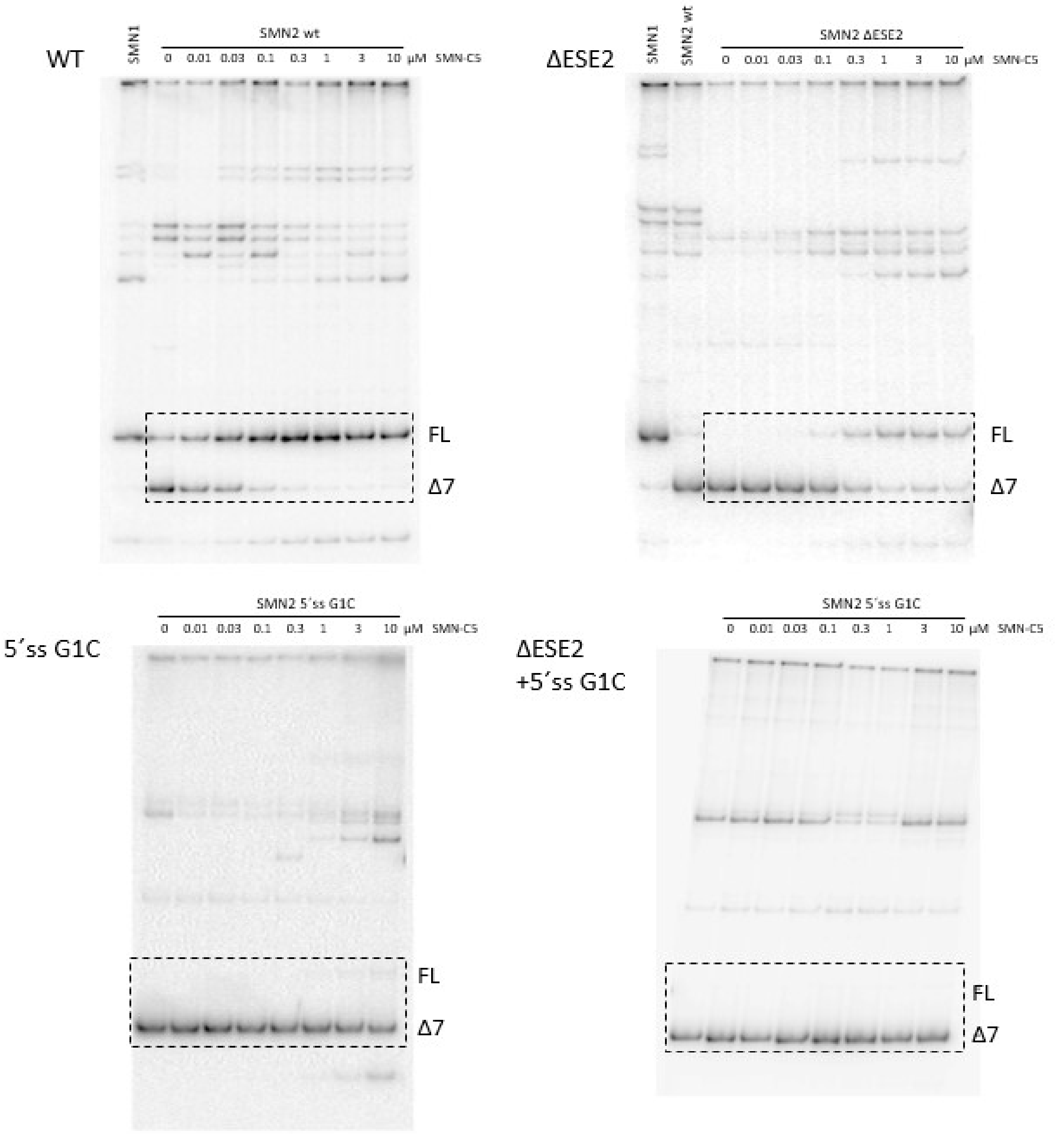


**Supplementary Figure 8.** Full blot pictures from Fig. 5A

Shown are full scans of the blots illustrated in Fig. 5A (Upper left: SMN2 wt; Upper right: SMN2 ΔESE2; Lower left: SMN2 5´ss G1C; Lower right: SMN2 ΔESE2+5´ss G1C). Dashed areas indicate the region shown in the main Figure.

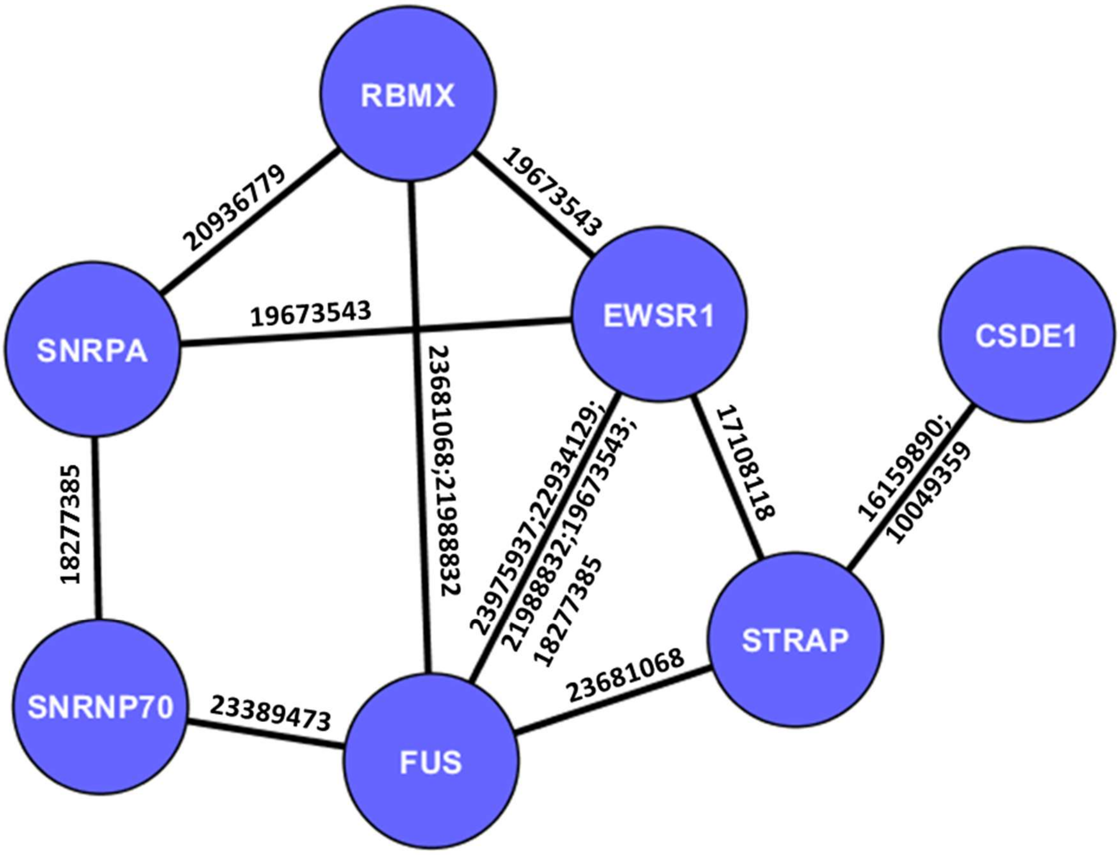


**Supplementary Figure 9.** Chemoproteomics and Protein pull down analysis with ESE2 construct

In nuclear extracts of SMA type 1 patient fibroblasts, pull downs were performed using ESE2 as bait. Out of 821 proteins identified, SMN-C6 specifically induced the binding of 10 proteins to ESE2 (pVal<0.05; unique peptides >1) which were not observed in the presence of the inactive compound SMN-C7. Based on String functional protein association analysis (string.db.org; high confidence 0.7), 7 of these proteins are described to be part of a complex: the U1 snRNP proteins A and 70; hnRNP G (also known as RBMX), FUS, EWSR1, STRAP, CSDE1. Numbers at the edges of the diagram indicate PubMed identifiers supporting the respective interactions.

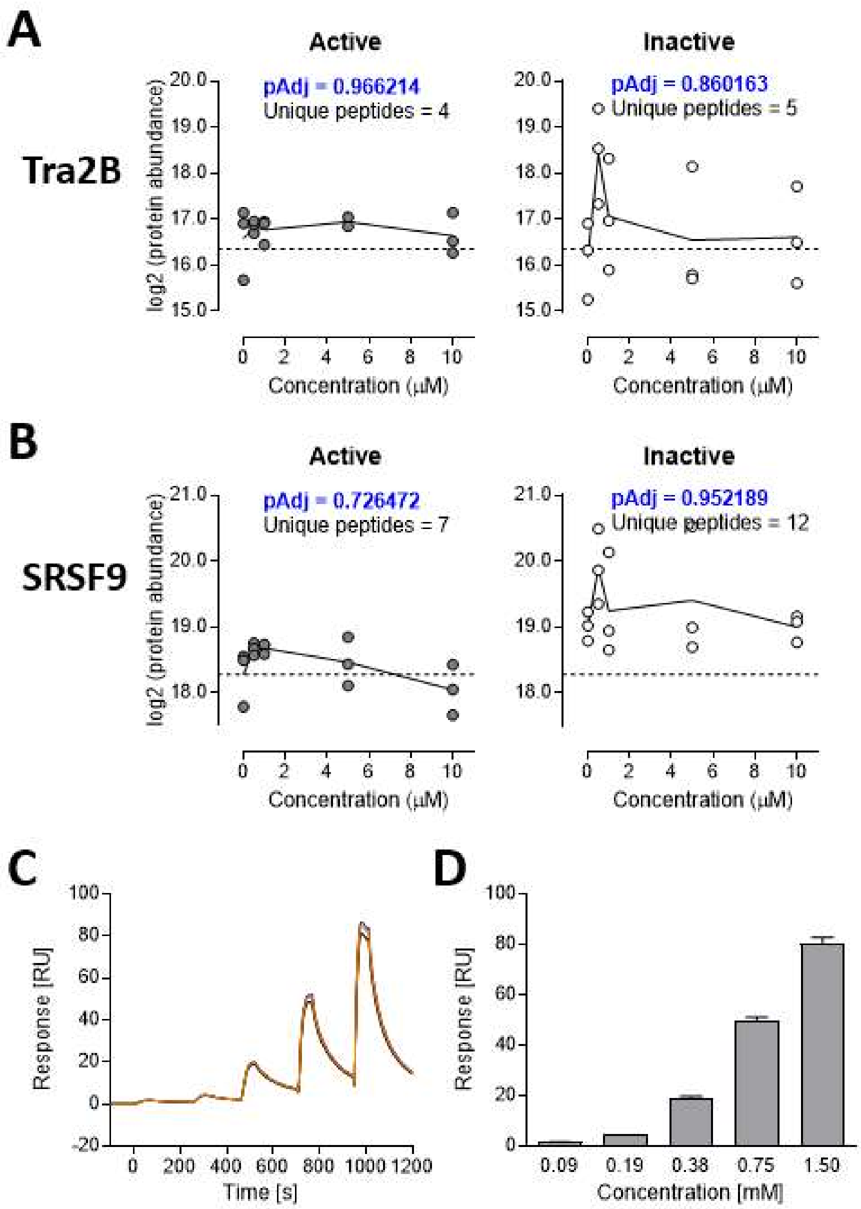


**Supplementary Figure 10.** Affinity Pull down enrichment of Tra2B and SRSF9 and Surface Plasmon Resonance of hnRNPG binding

(**A**) Protein abundance (log2) of Tra2B after affinity enrichment with the SMN2 ESE2 RNA sequence as bait, in the presence of Active (SMN-C6) or Inactive (SMN-C7) ligand. (**B**) Protein abundance (log2) of SRSF9 after affinity enrichment with the SMN2 ESE2 RNA sequence as bait, in the presence of increasing concentrations of Active (SMN-C6) or Inactive (SMN-C7) ligand. (**C**) SPR titration of hnRNPG (in quadruplicate) at 0.094-1.5 µM over immobilized ESE2 surface. Biotinylated ESE2 was immobilized on streptavidin surface on CAP sensor to densities of ~20 RU. Saturation of SPR signal for hnRNP G could not be achieved due to non-specific binding of hnRNP G onto sensor surface at concentrations higher than 1.5 µM. (**D**) Quantitative assessment of SPR signals monitored in (**C**). SPR signal amplitude was measured in the middle of the association phase. Data in (**D**) represent mean ± SEM of four independent experiments.

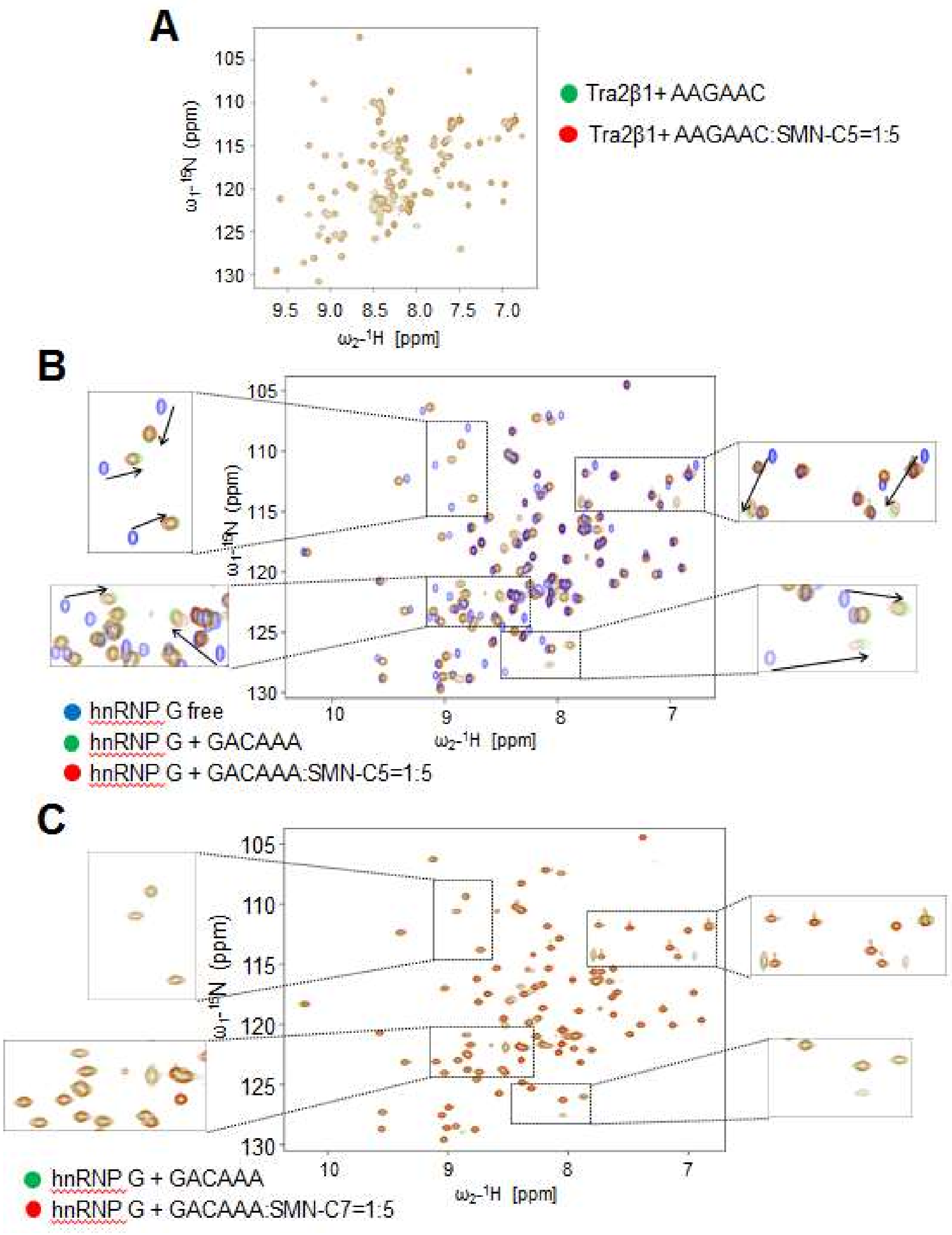


**Supplementary Figure 11.** Solution NMR binding of SMN-C5 to several protein-RNA complexes involved in *SMN2* splicing

(**A**) No chemical shift perturbations were observed when Tra2-β1 RRM in complex with RNA was titrated with SMN-C5 up to five fold in excess, indicating that SMN-C5 does not affect the interaction between Tra2-β1 and RNA. (**B**) In contrast, chemical shift perturbations were observed when hnRNP G RRM bound to RNA was titrated with SMN-C5. These perturbations were in the direction of the free form protein indicating that the compound destabilizes the interaction of hnRNP G RRM with the RNA. (**C**) The absence of chemical shift perturbation with the inactive compound SMN-C7 indicated that the effect of SMN-C5 observed on hnRNP G is specific.

## Supplementary Tables:

| Accession | Entry | Gene names | fold change active/inactive | adjusted p-value |
|---|---|---|---|---|
| ABCF1_HUMAN | Q8NE71 | ABCF1 ABC50 | 8,0 | 1,900E-08 |
| ABCF2_HUMAN | Q9UG63 | ABCF2 HUSSY-18 | 21,0 | 8,930E-09 |
| ACOT9_HUMAN | Q9Y305 | ACOT9 CGI-16 | 13,8 | 1,020E-08 |
| AGO2_HUMAN | Q9UKV8 | AGO2 EIF2C2 | 12,4 | 1,727E-04 |
| ANKZ1_HUMAN | Q9H8Y5 | ANKZF1 ZNF744 | 5,8 | 3,260E-05 |
| ANR17_HUMAN | O75179 | ANKRD17 GTAR KIAA0697 | 11,9 | 1,720E-06 |
| ANXA1_HUMAN | P04083 | ANXA1 ANX1 LPC1 | 3,5 | 1,485E-02 |
| ANXA2_HUMAN | P07355 | ANXA2 ANX2 ANX2L4 CAL1H LPC2D | 5,1 | 1,020E-06 |
| AP2A1_HUMAN | O95782 | AP2A1 ADTAA CLAPA1 | 4,2 | 5,190E-05 |
| AP2M1_HUMAN | Q96CW1 | AP2M1 CLAPM1 KIAA0109 | 12,4 | 3,820E-05 |
| ASCC3_HUMAN | Q8N3C0 | ASCC3 HELIC1 | 5,8 | 2,707E-04 |
| ASPH_HUMAN | Q12797 | ASPH | 12,1 | 5,920E-10 |
| ATD3A_HUMAN | Q9NVI7 | ATAD3A | 5,2 | 1,010E-05 |
| BOP1_HUMAN | Q14137 | BOP1 KIAA0124 | 8,1 | 5,150E-06 |
| BTF3_HUMAN | P20290 | BTF3 NACB OK/SW-cl.8 | 14,6 | 1,260E-08 |
| C1QBP_HUMAN | Q07021 | C1QBP GC1QBP HABP1 SF2P32 | 6,1 | 1,300E-07 |
| CA123_HUMAN | Q9NWV4 | C1orf123 | 20,1 | 1,840E-08 |
| CAPR1_HUMAN | Q14444 | CAPRIN1 GPIAP1 GPIP137 M11S1 RNG105 | 12,8 | 8,900E-07 |
| CC124_HUMAN | Q96CT7 | CCDC124 | 24,4 | 1,150E-10 |
| CCD80_HUMAN | Q76M96 | CCDC80 DRO1 URB HBE245 | 3,9 | 3,934E-03 |
| CDC5L_HUMAN | Q99459 | CDC5L KIAA0432 PCDC5RP | 9,4 | 1,640E-09 |
| CG050_HUMAN | Q8WUJ3 | CEMIP KIAA1199 | 21,9 | 6,650E-10 |
| CH033_HUMAN | Q9BRJ6 | C7orf50 FP15621 | 12,1 | 2,530E-05 |
| CHD3_HUMAN | Q9H7E9 | C8orf33 | 12,1 | 1,200E-06 |
| CHD4_HUMAN | Q12873 | CHD3 | 9,9 | 1,590E-05 |
| CHTOP_HUMAN | Q14839 | CHD4 | 7,7 | 2,300E-07 |
| CIRBP_HUMAN | Q9Y3Y2 | CHTOP C1orf77 FOP HT031 PP7704 | 15,2 | 1,650E-08 |
| CKAP4_HUMAN | Q14011 | CIRBP A18HNRNP CIRP | 15,8 | 2,180E-10 |
| CN166_HUMAN | Q07065 | CKAP4 | 4,9 | 3,540E-06 |
| CO1A1_HUMAN | Q9Y224 | C14orf166 CGI-99 | 8,4 | 2,090E-05 |
| CO1A2_HUMAN | P02452 | COL1A1 | 4,5 | 9,290E-06 |
| CO6A3_HUMAN | P08123 | COL1A2 | 3,2 | 7,410E-05 |
| COCA1_HUMAN | P12111 | COL6A3 | 3,2 | 4,630E-06 |
| COPA_HUMAN | Q99715 | COL12A1 COL12A1L | 5,2 | 1,730E-06 |
| CPNE1_HUMAN | P53621 | COPA | 14,4 | 1,790E-09 |
| CPNE3_HUMAN | Q99829 | CPNE1 CPN1 | 14,1 | 6,170E-10 |
| CPNE7_HUMAN | O75131 | CPNE3 CPN3 KIAA0636 | 14,5 | 5,790E-06 |
| CSDE1_HUMAN | Q9UBL6 | CPNE7 | 13,3 | 2,090E-08 |
| CTCF_HUMAN | O75534 | CSDE1 D1S155E KIAA0885 NRU UNR | 15,3 | 6,250E-07 |
| CYR61_HUMAN | P49711 | CTCF | 11,7 | 2,010E-06 |
| DDX1_HUMAN | O00622 | CYR61 CCN1 GIG1 IGFBP10 | 6,3 | 3,020E-09 |
| DDX17_HUMAN | Q92841 | DDX17 | 13,8 | 3,100E-10 |

| | | | | |
|---|---|---|---|---|
| DDX18_HUMAN | Q9NVP1 | DDX18 | 8,9 | 3,690E-08 |
| DDX21_HUMAN | Q92499 | DDX1 | 11,8 | 1,590E-08 |
| DDX24_HUMAN | Q9NR30 | DDX21 | 14,7 | 2,820E-11 |
| DDX27_HUMAN | Q9GZR7 | DDX24 | 10,8 | 1,450E-07 |
| DDX3X_HUMAN | Q96GQ7 | DDX27 RHLP HSPC259 PP3241 | 5,8 | 3,860E-10 |
| DDX5_HUMAN | O00571 | DDX3X DBX DDX3 | 14,8 | 6,440E-11 |
| DDX50_HUMAN | Q9BQ39 | DDX50 | 8,6 | 7,480E-08 |
| DDX54_HUMAN | Q8TDD1 | DDX54 | 10,2 | 1,480E-07 |
| DDX55_HUMAN | Q8NHQ9 | DDX55 KIAA1595 | 7,3 | 6,480E-07 |
| DDX6_HUMAN | P17844 | DDX5 G17P1 HELR HLR1 | 14,1 | 5,290E-10 |
| DECR_HUMAN | P26196 | DDX6 HLR2 RCK | 16,2 | 3,365E-04 |
| DHX15_HUMAN | Q16698 | DECR1 DECR | 6,7 | 4,600E-05 |
| DHX29_HUMAN | O43143 | DHX15 DBP1 DDX15 | 14,9 | 9,540E-11 |
| DHX30_HUMAN | Q7Z478 | DHX29 DDX29 | 13,9 | 1,830E-08 |
| DHX57_HUMAN | Q7L2E3 | DHX30 DDX30 KIAA0890 | 10,4 | 5,360E-09 |
| DHX9_HUMAN | Q6P158 | DHX57 | 7,4 | 3,040E-09 |
| DJC10_HUMAN | Q08211 | DHX9 DDX9 LKP NDH2 | 5,7 | 8,130E-06 |
| DJC21_HUMAN | Q8IXB1 | DNAJC10 ERDJ5 UNQ495/PRO1012 | 14,7 | 4,170E-06 |
| DKC1_HUMAN | Q5F1R6 | DNAJC21 DNAJA5 | 11,1 | 2,707E-04 |
| DSRAD_HUMAN | O60832 | DKC1 NOLA4 | 9,9 | 6,770E-09 |
| DX39A_HUMAN | P55265 | ADAR ADAR1 DSRAD G1P1 IFI4 | 7,4 | 1,450E-07 |
| EBP2_HUMAN | O00148 | DDX39A DDX39 | 34,4 | 1,640E-08 |
| EF2_HUMAN | Q99848 | EBNA1BP2 EBP2 | 5,5 | 7,010E-05 |
| EFTU_HUMAN | P13639 | EEF2 EF2 | 21,9 | 4,990E-08 |
| EIF3A_HUMAN | P49411 | TUFM | 6,9 | 2,300E-10 |
| EIF3B_HUMAN | Q14152 | EIF3A EIF3S10 KIAA0139 | 4,3 | 2,839E-03 |
| EIF3C_HUMAN | P55884 | EIF3B EIF3S9 | 7,0 | 1,160E-07 |
| EIF3D_HUMAN | Q99613 | EIF3C EIF3S8 | 8,6 | 2,430E-10 |
| EIF3E_HUMAN | O15371 | EIF3D EIF3S7 | 4,4 | 5,892E-04 |
| EIF3F_HUMAN | P60228 | EIF3E EIF3S6 INT6 | 6,2 | 1,103E-03 |
| EIF3I_HUMAN | O00303 | EIF3F EIF3S5 | 7,7 | 5,950E-10 |
| EIF3L_HUMAN | Q13347 | EIF3I EIF3S2 TRIP1 | 6,2 | 1,010E-05 |
| ELAV1_HUMAN | Q9Y262 | EIF3L EIF3EIP EIF3S6IP HSPC021 HSPC025 MSTP005 | 16,9 | 1,120E-09 |
| ERF1_HUMAN | Q15717 | ELAVL1 HUR | 11,1 | 3,180E-06 |
| EXOS1_HUMAN | P62495 | ETF1 ERF1 RF1 SUP45L1 | 11,7 | 1,485E-02 |
| EXOSX_HUMAN | Q9Y3B2 | EXOSC1 CSL4 CGI-108 | 15,3 | 1,450E-07 |
| F120A_HUMAN | Q01780 | EXOSC10 PMSCL PMSCL2 RRP6 | 14,8 | 8,440E-10 |
| FA98A_HUMAN | Q9NZB2 | FAM120A C9orf10 KIAA0183 OSSA | 5,6 | 3,070E-08 |
| FBN1_HUMAN | Q8NCA5 | FAM98A | 6,6 | 3,100E-08 |
| FBRL_HUMAN | P35555 | FBN1 FBN | 16,0 | 6,010E-09 |
| FINC_HUMAN | P22087 | FBL FIB1 FLRN | 6,0 | 2,780E-08 |
| FLII_HUMAN | P02751 | FN1 FN | 3,4 | 1,380E-05 |
| FMR1_HUMAN | Q13045 | FLII FLIL | 12,2 | 8,670E-05 |
| FUS_HUMAN | Q06787 | FMR1 | 3,3 | 1,170E-06 |
| FXR1_HUMAN | P35637 | FUS TLS | 19,5 | 1,080E-09 |
| FXR2_HUMAN | P51114 | FXR1 | 7,2 | 9,380E-07 |

| | | | | |
|---|---|---|---|---|
| G3BP1_HUMAN | P51116 | FXR2 FMR1L2 | 6,2 | 5,790E-06 |
| G3BP2_HUMAN | Q13283 | G3BP1 G3BP | 14,1 | 4,500E-06 |
| GAR1_HUMAN | Q9UN86 | G3BP2 KIAA0660 | 10,1 | 1,680E-06 |
| GBLP_HUMAN | Q9NY12 | GAR1 NOLA1 | 12,0 | 6,790E-06 |
| GELS_HUMAN | P63244 | GNB2L1 HLC7 PIG21 | 2,9 | 1,607E-04 |
| GNL1_HUMAN | P06396 | GSN | 5,1 | 7,840E-06 |
| GNL3_HUMAN | P36915 | GNL1 HSR1 | 18,2 | 5,810E-10 |
| GREM1_HUMAN | Q9BVP2 | GNL3 E2IG3 NS | 13,8 | 1,670E-07 |
| GRP78_HUMAN | O60565 | GREM1 CKTSF1B1 DAND2 DRM PIG2 | 4,0 | 1,010E-05 |
| GRSF1_HUMAN | P11021 | HSPA5 GRP78 | 12,5 | 1,160E-08 |
| GRWD1_HUMAN | Q12849 | GRSF1 | 14,1 | 5,640E-10 |
| GSCR2_HUMAN | Q9BQ67 | GRWD1 GRWD KIAA1942 WDR28 | 8,4 | 1,781E-03 |
| H13_HUMAN | Q9NZM5 | GLTSCR2 | 22,1 | 2,830E-08 |
| H15_HUMAN | P16402 | HIST1H1D H1F3 | 30,2 | 6,300E-08 |
| H1X_HUMAN | P16401 | HIST1H1B H1F5 | 22,0 | 4,670E-08 |
| H2A1A_HUMAN | Q92522 | H1FX | 29,0 | 5,950E-10 |
| H2A1B_HUMAN | Q96QV6 | HIST1H2AA H2AFR | 36,2 | 2,300E-10 |
| H2A1D_HUMAN | P04908 | HIST1H2AB H2AFM; HIST1H2AE H2AFA | 29,9 | 1,440E-10 |
| H2AY_HUMAN | P20671 | HIST1H2AD H2AFG | 57,4 | 1,984E-03 |
| H31_HUMAN | O75367 | H2AFY MACROH2A1 | 27,1 | 2,300E-07 |
| H4_HUMAN | P68431 | HIST1H3A H3FA | 32,7 | 3,990E-10 |
| HCD2_HUMAN | P62805 | HIST1H4A H4/A H4FA | 11,7 | 2,181E-03 |
| HERC2_HUMAN | Q99714 | HSD17B10 ERAB HADH2 MRPP2 SCHAD XH98G2 | 6,8 | 2,348E-03 |
| HNRDL_HUMAN | O95714 | HERC2 | 13,5 | 7,850E-10 |
| HNRH1_HUMAN | O14979 | HNRNPDL HNRPDL JKTBP | 8,8 | 1,260E-08 |
| HNRH2_HUMAN | P31943 | HNRNPH1 HNRPH HNRPH1 | 11,4 | 3,010E-09 |
| HNRH3_HUMAN | P55795 | HNRNPH2 FTP3 HNRPH2 | 13,1 | 2,180E-09 |
| HNRL1_HUMAN | P31942 | HNRNPH3 HNRPH3 | 12,8 | 1,780E-09 |
| HNRL2_HUMAN | Q9BUJ2 | HNRNPUL1 E1BAP5 HNRPUL1 | 11,5 | 1,960E-11 |
| HNRPC_HUMAN | Q1KMD3 | HNRNPUL2 HNRPUL2 | 12,2 | 9,940E-10 |
| HNRPD_HUMAN | P07910 | HNRNPC HNRPC | 12,1 | 1,490E-09 |
| HNRPF_HUMAN | Q14103 | HNRNPD AUF1 HNRPD | 8,5 | 2,568E-03 |
| HNRPK_HUMAN | P52597 | HNRNPF HNRPF | 15,7 | 7,650E-11 |
| HNRPL_HUMAN | P61978 | HNRNPK HNRPK | 13,9 | 5,460E-11 |
| HNRPM_HUMAN | P14866 | HNRNPL HNRPL P/OKcl.14 | 15,7 | 7,500E-12 |
| HNRPQ_HUMAN | P52272 | HNRNPM HNRPM NAGR1 | 19,6 | 2,820E-11 |
| HNRPR_HUMAN | O60506 | SYNCRIP HNRPQ NSAP1 | 21,1 | 8,970E-11 |
| HNRPU_HUMAN | O43390 | HNRNPR HNRPR | 5,4 | 3,150E-07 |
| HP1B3_HUMAN | Q00839 | HNRNPU HNRPU SAFA U21.1 | 12,6 | 5,930E-07 |
| HSP7C_HUMAN | Q5SSJ5 | HP1BP3 | 3,0 | 9,450E-08 |
| IF2B1_HUMAN | P11142 | HSPA8 HSC70 HSP73 HSPA10 | 14,7 | 2,510E-08 |
| IF2B2_HUMAN | Q9NZI8 | IGF2BP1 CRDBP VICKZ1 ZBP1 | 22,6 | 6,440E-11 |
| IF2B3_HUMAN | Q9Y6M1 | IGF2BP2 IMP2 VICKZ2 | 22,3 | 4,570E-10 |
| IF2G_HUMAN | O00425 | IGF2BP3 IMP3 KOC1 VICKZ3 | 5,8 | 2,810E-06 |
| IF2P_HUMAN | P41091 | EIF2S3 EIF2G | 7,2 | 2,110E-05 |
| IF4A1_HUMAN | O60841 | EIF5B IF2 KIAA0741 | 7,1 | 7,890E-08 |

| | | | | |
|---|---|---|---|---|
| IF5A1_HUMAN | P60842 | EIF4A1 DDX2A EIF4A | 18,9 | 1,500E-08 |
| IF6_HUMAN | P63241 | EIF5A | 24,7 | 9,820E-09 |
| IFIT5_HUMAN | P56537 | EIF6 EIF3A ITGB4BP OK/SW-cl.27 | 12,7 | 2,490E-07 |
| IFRD1_HUMAN | Q13325 | IFIT5 ISG58 RI58 | 13,0 | 8,150E-09 |
| ILF2_HUMAN | O00458 | IFRD1 | 20,2 | 1,310E-10 |
| ILF3_HUMAN | Q12905 | ILF2 NF45 PRO3063 | 17,2 | 2,990E-11 |
| IMA1_HUMAN | Q12906 | ILF3 DRBF MPHOSPH4 NF90 | 16,5 | 1,790E-07 |
| IQGA1_HUMAN | P52292 | KPNA2 RCH1 SRP1 | 7,6 | 3,040E-07 |
| K0020_HUMAN | P46940 | IQGAP1 KIAA0051 | 12,0 | 3,070E-08 |
| K1199_HUMAN | Q15397 | KIAA0020 XTP5 | 10,7 | 2,830E-10 |
| KRI1_HUMAN | Q8N9T8 | KRI1 | 3,6 | 5,330E-05 |
| LA_HUMAN | P83111 | LACTB MRPL56 UNQ843/PRO1781 | 27,9 | 1,870E-07 |
| LACTB_HUMAN | Q92615 | LARP4B KIAA0217 LARP5 | 7,7 | 3,330E-07 |
| LAR4B_HUMAN | Q6PKG0 | LARP1 KIAA0731 LARP | 7,6 | 6,098E-03 |
| LARP1_HUMAN | Q71RC2 | LARP4 PP13296 | 13,6 | 9,970E-08 |
| LARP4_HUMAN | Q9Y4W2 | LAS1L MSTP060 | 28,0 | 1,250E-06 |
| LAS1L_HUMAN | P05455 | SSB | 3,7 | 2,919E-04 |
| LOXL2_HUMAN | Q9Y4K0 | LOXL2 | 4,6 | 5,412E-04 |
| LRC59_HUMAN | Q96AG4 | LRRC59 PRO1855 | 12,8 | 3,300E-09 |
| LRC8A_HUMAN | Q8IWT6 | LRRC8A KIAA1437 LRRC8 SWELL1 UNQ221/PRO247 | 4,1 | 1,074E-03 |
| LSG1_HUMAN | Q9H089 | LSG1 | 9,3 | 8,250E-08 |
| LYAR_HUMAN | Q9NX58 | LYAR PNAS-5 | 9,1 | 4,960E-05 |
| LYRIC_HUMAN | Q86UE4 | MTDH AEG1 LYRIC | 11,7 | 2,730E-08 |
| MA2C1_HUMAN | Q9NTJ4 | MAN2C1 MANA MANA1 | 8,5 | 2,180E-10 |
| MA7D1_HUMAN | Q3KQU3 | MAP7D1 KIAA1187 PARCC1 RPRC1 PP2464 | 8,5 | 3,610E-05 |
| MAP1A_HUMAN | P78559 | MAP1A MAP1L | 12,5 | 1,210E-07 |
| MATR3_HUMAN | P43243 | MATR3 KIAA0723 | 10,2 | 4,520E-11 |
| MBB1A_HUMAN | Q9BQG0 | MYBBP1A P160 | 15,9 | 5,340E-10 |
| MICA1_HUMAN | Q8TDZ2 | MICAL1 MICAL NICAL | 5,6 | 5,506E-04 |
| MK67I_HUMAN | Q9BYG3 | NIFK MKI67IP NOPP34 | 10,5 | 1,410E-07 |
| MOV10_HUMAN | Q9HCE1 | MOV10 KIAA1631 | 13,1 | 1,610E-06 |
| MRCKA_HUMAN | Q5VT25 | CDC42BPA KIAA0451 | 9,7 | 3,470E-06 |
| MRT4_HUMAN | Q9UKD2 | MRTO4 C1orf33 MRT4 | 20,0 | 1,840E-08 |
| MYO1B_HUMAN | O43795 | MYO1B | 4,0 | 2,090E-05 |
| MYO1C_HUMAN | O00159 | MYO1C | 6,8 | 6,440E-11 |
| MYO1D_HUMAN | O94832 | MYO1D KIAA0727 | 12,6 | 3,150E-11 |
| MYO5A_HUMAN | Q9Y4I1 | MYO5A MYH12 | 9,0 | 5,450E-06 |
| NACA_HUMAN | Q13765 | NACA HSD48 | 14,6 | 8,900E-09 |
| NAT10_HUMAN | Q9H0A0 | NAT10 ALP KIAA1709 | 17,2 | 1,190E-09 |
| NCBP1_HUMAN | Q09161 | NCBP1 CBP80 NCBP | 10,1 | 9,670E-05 |
| NEMF_HUMAN | O60524 | NEMF SDCCAG1 | 15,2 | 3,180E-07 |
| NH2L1_HUMAN | P55769 | NHP2L1 | 29,6 | 7,930E-09 |
| NHP2_HUMAN | Q9NX24 | NHP2 NOLA2 HSPC286 | 14,4 | 9,450E-08 |
| NMD3_HUMAN | Q96D46 | NMD3 CGI-07 | 10,9 | 2,805E-04 |
| NOA1_HUMAN | Q8NC60 | NOA1 C4orf14 | 8,9 | 6,621E-04 |
| NOG1_HUMAN | Q9BZE4 | GTPBP4 CRFG NOG1 | 18,2 | 3,950E-07 |

| | | | | |
|---|---|---|---|---|
| NOG2_HUMAN | Q13823 | GNL2 NGP1 | 11,6 | 1,110E-06 |
| NOL6_HUMAN | Q9H6R4 | NOL6 | 10,0 | 5,250E-06 |
| NONO_HUMAN | Q15233 | NONO NRB54 | 7,9 | 1,220E-07 |
| NOP10_HUMAN | Q9NPE3 | NOP10 NOLA3 | 26,3 | 1,880E-06 |
| NOP16_HUMAN | Q9Y3C1 | NOP16 CGI-117 HSPC111 | 26,0 | 2,730E-08 |
| NOP2_HUMAN | P46087 | NOP2 NOL1 | 15,3 | 2,880E-11 |
| NOP56_HUMAN | O00567 | NOP56 NOL5A | 18,2 | 1,160E-08 |
| NOP58_HUMAN | Q9Y2X3 | NOP58 NOL5 NOP5 HSPC120 | 7,5 | 4,230E-07 |
| NP1L1_HUMAN | P55209 | NAP1L1 NRP | 11,7 | 2,050E-08 |
| NP1L4_HUMAN | Q99733 | NAP1L4 NAP2 | 11,1 | 7,250E-05 |
| NPM_HUMAN | P06748 | NPM1 NPM | 22,6 | 3,410E-10 |
| NSUN2_HUMAN | Q08J23 | NSUN2 SAKI TRM4 | 11,9 | 4,570E-10 |
| NUCL_HUMAN | P19338 | NCL | 12,7 | 3,840E-06 |
| NXF1_HUMAN | Q9UBU9 | NXF1 TAP | 5,1 | 2,684E-03 |
| OLA1_HUMAN | Q9NTK5 | OLA1 GTPBP9 PRO2455 PTD004 | 14,3 | 9,950E-06 |
| P4HA1_HUMAN | P13674 | P4HA1 P4HA | 4,5 | 2,180E-08 |
| P4HA2_HUMAN | O15460 | P4HA2 UNQ290/PRO330 | 6,0 | 4,750E-10 |
| PA2G4_HUMAN | Q9UQ80 | PA2G4 EBP1 | 13,8 | 2,350E-06 |
| PAB4L_HUMAN | P0CB38 | PABPC4L | 27,0 | 4,800E-10 |
| PABP1_HUMAN | P11940 | PABPC1 PAB1 PABP1 PABPC2 | 17,6 | 1,340E-07 |
| PABP2_HUMAN | Q86U42 | PABPN1 PAB2 PABP2 | 12,1 | 2,350E-06 |
| PABP3_HUMAN | Q9H361 | PABPC3 PABP3 PABPL3 | 32,4 | 1,880E-09 |
| PABP4_HUMAN | Q13310 | PABPC4 APP1 PABP4 | 22,0 | 2,180E-10 |
| PAIRB_HUMAN | Q8NC51 | SERBP1 PAIRBP1 CGI-55 | 19,8 | 1,060E-06 |
| PCBP1_HUMAN | Q15365 | PCBP1 | 8,2 | 1,640E-08 |
| PCBP2_HUMAN | Q15366 | PCBP2 | 14,2 | 2,740E-09 |
| PDCD4_HUMAN | Q53EL6 | PDCD4 H731 | 15,3 | 6,770E-09 |
| PDIA1_HUMAN | P07237 | P4HB ERBA2L PDI PDIA1 PO4DB | 4,9 | 3,560E-08 |
| PESC_HUMAN | O00541 | PES1 | 7,3 | 5,730E-08 |
| PLEC_HUMAN | Q15149 | PLEC PLEC1 | 17,2 | 1,490E-06 |
| PLOD1_HUMAN | Q02809 | PLOD1 LLH PLOD | 8,9 | 1,840E-08 |
| PLOD3_HUMAN | O60568 | PLOD3 | 4,4 | 2,510E-06 |
| PLPL6_HUMAN | Q8IY17 | PNPLA6 NTE | 5,5 | 3,900E-05 |
| POP1_HUMAN | Q99575 | POP1 KIAA0061 | 6,5 | 1,030E-05 |
| PRC2C_HUMAN | Q9Y520 | PRRC2C BAT2D1 BAT2L2 KIAA1096 XTP2 | 7,7 | 2,510E-06 |
| PRDBP_HUMAN | Q969G5 | PRKCDBP SRBC | 12,3 | 1,370E-06 |
| PRKRA_HUMAN | O75569 | PRKRA PACT RAX HSD-14 HSD14 | 15,9 | 1,500E-07 |
| PRP19_HUMAN | Q9UMS4 | PRPF19 NMP200 PRP19 SNEV | 10,6 | 2,750E-07 |
| PRP4_HUMAN | O43172 | PRPF4 PRP4 | 13,3 | 3,910E-07 |
| PRP8_HUMAN | Q6P2Q9 | PRPF8 PRPC8 | 4,2 | 1,578E-04 |
| PTBP1_HUMAN | P26599 | PTBP1 PTB | 15,1 | 4,520E-11 |
| PTRF_HUMAN | Q6NZI2 | PTRF FKSG13 | 18,0 | 1,140E-08 |
| PURA_HUMAN | Q00577 | PURA PUR1 | 34,2 | 5,920E-10 |
| PURB_HUMAN | Q96QR8 | PURB | 26,7 | 5,340E-10 |
| PWP1_HUMAN | Q13610 | PWP1 | 12,0 | 5,490E-09 |
| RALY_HUMAN | Q9UKM9 | RALY HNRPCL2 P542 | 13,6 | 4,560E-07 |

| | | | | |
|---|---|---|---|---|
| RBM28_HUMAN | Q9NW13 | RBM28 | 9,0 | 4,340E-05 |
| RBM3_HUMAN | P42696 | RBM34 KIAA0117 | 11,8 | 2,250E-09 |
| RBM34_HUMAN | Q14498 | RBM39 HCC1 RNPC2 | 11,0 | 5,550E-06 |
| RBM39_HUMAN | P98179 | RBM3 RNPL | 12,7 | 3,890E-09 |
| RBM4_HUMAN | Q9BQ04 | RBM4B RBM30 | 16,1 | 4,120E-07 |
| RBM4B_HUMAN | Q9BWF3 | RBM4 RBM4A | 46,9 | 4,720E-06 |
| RBM8A_HUMAN | Q9Y5S9 | RBM8A RBM8 HSPC114 MDS014 | 14,5 | 6,620E-08 |
| RBMS1_HUMAN | P29558 | RBMS1 C2orf12 MSSP MSSP1 SCR2 | 13,5 | 5,690E-08 |
| RBMS2_HUMAN | Q15434 | RBMS2 SCR3 | 11,2 | 1,173E-04 |
| RBMS3_HUMAN | Q6XE24 | RBMS3 | 11,9 | 7,840E-06 |
| RBMX_HUMAN | P38159 | RBMX HNRPG RBMXP1 | 8,1 | 6,770E-09 |
| RBP56_HUMAN | Q92804 | TAF15 RBP56 TAF2N | 3,5 | 1,270E-07 |
| RED1_HUMAN | P78563 | ADARB1 ADAR2 DRADA2 RED1 | 7,3 | 2,110E-07 |
| RENT1_HUMAN | Q92900 | UPF1 KIAA0221 RENT1 | 7,6 | 1,120E-06 |
| RL10_HUMAN | P62906 | RPL10A NEDD6 | 42,3 | 5,560E-08 |
| RL10A_HUMAN | P27635 | RPL10 DXS648E QM | 31,7 | 8,700E-08 |
| RL11_HUMAN | P62913 | RPL11 | 23,2 | 5,270E-07 |
| RL12_HUMAN | P30050 | RPL12 | 48,4 | 2,550E-11 |
| RL13_HUMAN | P40429 | RPL13A | 32,1 | 3,570E-08 |
| RL13A_HUMAN | P26373 | RPL13 BBC1 OK/SW-cl.46 | 27,5 | 4,310E-08 |
| RL14_HUMAN | P50914 | RPL14 | 34,2 | 1,290E-08 |
| RL15_HUMAN | P61313 | RPL15 EC45 TCBAP0781 | 25,1 | 8,490E-09 |
| RL17_HUMAN | P18621 | RPL17 | 33,2 | 1,260E-08 |
| RL18_HUMAN | Q02543 | RPL18A | 34,9 | 4,180E-08 |
| RL18A_HUMAN | Q07020 | RPL18 | 38,4 | 1,020E-08 |
| RL19_HUMAN | P84098 | RPL19 | 32,2 | 8,200E-09 |
| RL1D1_HUMAN | O76021 | RSL1D1 CATX11 CSIG PBK1 L12 | 38,6 | 4,150E-09 |
| RL21_HUMAN | P46778 | RPL21 | 36,9 | 5,360E-09 |
| RL22_HUMAN | Q6P5R6 | RPL22L1 | 42,7 | 9,970E-13 |
| RL22L_HUMAN | P35268 | RPL22 | 48,9 | 8,970E-11 |
| RL23_HUMAN | P62750 | RPL23A | 39,4 | 2,820E-11 |
| RL23A_HUMAN | P62829 | RPL23 | 31,5 | 1,200E-07 |
| RL24_HUMAN | P83731 | RPL24 | 17,1 | 3,410E-10 |
| RL26_HUMAN | Q9UNX3 | RPL26L1 RPL26P1 | 31,5 | 8,240E-08 |
| RL26L_HUMAN | P61254 | RPL26 | 40,6 | 8,100E-11 |
| RL27_HUMAN | P46776 | RPL27A | 56,1 | 8,920E-11 |
| RL27A_HUMAN | P61353 | RPL27 | 65,0 | 1,900E-11 |
| RL28_HUMAN | P46779 | RPL28 | 61,9 | 2,690E-11 |
| RL29_HUMAN | P47914 | RPL29 | 27,8 | 7,840E-08 |
| RL3_HUMAN | P62888 | RPL30 | 37,5 | 1,740E-09 |
| RL30_HUMAN | P62899 | RPL31 | 71,8 | 1,250E-10 |
| RL31_HUMAN | P62910 | RPL32 PP9932 | 40,8 | 6,440E-11 |
| RL32_HUMAN | P49207 | RPL34 | 32,3 | 1,290E-11 |
| RL34_HUMAN | P18077 | RPL35A GIG33 | 49,0 | 1,690E-10 |
| RL35_HUMAN | P42766 | RPL35 | 44,6 | 1,570E-09 |
| RL35A_HUMAN | P83881 | RPL36A RPL44 GIG15 MIG6 | 52,4 | 2,040E-11 |

| | | | | |
|---|---|---|---|---|
| RL36_HUMAN | Q9Y3U8 | RPL36 | 43,2 | 4,520E-11 |
| RL36A_HUMAN | P61513 | RPL37A | 47,8 | 1,840E-10 |
| RL37A_HUMAN | P63173 | RPL38 | 53,2 | 2,900E-10 |
| RL38_HUMAN | P39023 | RPL3 OK/SW-cl.32 | 127,7 | 1,230E-10 |
| RL4_HUMAN | P62987 | UBA52 UBCEP2 | 38,8 | 5,830E-08 |
| RL40_HUMAN | P36578 | RPL4 RPL1 | 18,5 | 2,151E-03 |
| RL5_HUMAN | P46777 | RPL5 MSTP030 | 42,8 | 1,440E-09 |
| RL6_HUMAN | Q02878 | RPL6 TXREB1 | 41,2 | 1,330E-09 |
| RL7_HUMAN | P62424 | RPL7A SURF-3 SURF3 | 34,5 | 1,780E-08 |
| RL7A_HUMAN | Q6DKI1 | RPL7L1 | 29,6 | 1,670E-05 |
| RL7L_HUMAN | P18124 | RPL7 | 49,3 | 3,530E-06 |
| RL8_HUMAN | P62917 | RPL8 | 19,7 | 1,980E-05 |
| RL9_HUMAN | P32969 | RPL9 OK/SW-cl.103; RPL9P7; RPL9P8; RPL9P9 | 38,1 | 2,290E-08 |
| RLA0_HUMAN | P05388 | RPLP0 | 10,8 | 1,440E-05 |
| RLA1_HUMAN | P05386 | RPLP1 RRP1 | 30,7 | 2,090E-09 |
| RLA2_HUMAN | P05387 | RPLP2 D11S2243E RPP2 | 57,1 | 5,510E-13 |
| RM01_HUMAN | Q9BYD6 | MRPL1 BM-022 | 7,1 | 4,604E-04 |
| RM23_HUMAN | Q16540 | MRPL23 L23MRP RPL23L | 12,6 | 2,750E-07 |
| RN213_HUMAN | Q63HN8 | RNF213 ALO17 C17orf27 KIAA1554 KIAA1618 MYSTR | 7,0 | 1,385E-04 |
| RO60_HUMAN | P10155 | TROVE2 RO60 SSA2 | 11,4 | 2,820E-05 |
| ROA0_HUMAN | Q13151 | HNRNPA0 HNRPA0 | 11,6 | 1,560E-07 |
| ROA1_HUMAN | P09651 | HNRNPA1 HNRPA1 | 18,2 | 2,710E-10 |
| ROA2_HUMAN | P22626 | HNRNPA2B1 HNRPA2B1 | 14,0 | 4,130E-10 |
| ROA3_HUMAN | P51991 | HNRNPA3 HNRPA3 | 10,4 | 5,760E-08 |
| ROAA_HUMAN | Q99729 | HNRNPAB ABBP1 HNRPAB | 17,4 | 5,920E-10 |
| RPF2_HUMAN | Q9H7B2 | RPF2 BXDC1 | 19,0 | 6,320E-09 |
| RPN1_HUMAN | P04843 | RPN1 | 7,5 | 2,280E-07 |
| RPN2_HUMAN | P04844 | RPN2 | 5,2 | 1,450E-07 |
| RRBP1_HUMAN | Q9P2E9 | RRBP1 KIAA1398 | 3,4 | 5,790E-06 |
| RRP1_HUMAN | Q5JTH9 | RRP12 KIAA0690 | 23,5 | 2,165E-04 |
| RRP12_HUMAN | Q14684 | RRP1B KIAA0179 | 7,6 | 4,490E-08 |
| RRP1B_HUMAN | P56182 | RRP1 D21S2056E NNP1 NOP52 RRP1A | 12,4 | 2,650E-06 |
| RRP5_HUMAN | Q14690 | PDCD11 KIAA0185 | 4,3 | 3,554E-04 |
| RRP8_HUMAN | O43159 | RRP8 KIAA0409 NML hucep-1 | 13,5 | 1,210E-07 |
| RRS1_HUMAN | Q15050 | RRS1 KIAA0112 RRR | 11,7 | 1,260E-07 |
| RS10_HUMAN | P46783 | RPS10 | 46,1 | 6,920E-13 |
| RS11_HUMAN | P62280 | RPS11 | 48,3 | 5,950E-10 |
| RS12_HUMAN | P25398 | RPS12 | 43,3 | 5,510E-13 |
| RS13_HUMAN | P62277 | RPS13 | 59,8 | 8,370E-13 |
| RS14_HUMAN | P62263 | RPS14 PRO2640 | 55,7 | 5,510E-13 |
| RS15_HUMAN | P62244 | RPS15A OK/SW-cl.82 | 21,4 | 1,250E-10 |
| RS15A_HUMAN | P62841 | RPS15 RIG | 40,2 | 8,920E-11 |
| RS16_HUMAN | P62249 | RPS16 | 55,9 | 3,150E-11 |
| RS17L_HUMAN | P0CW22 | RPS17L | 39,3 | 3,630E-11 |
| RS18_HUMAN | P62269 | RPS18 D6S218E | 55,7 | 6,440E-11 |
| RS19_HUMAN | P39019 | RPS19 | 63,6 | 3,140E-11 |

| | | | | |
|---|---|---|---|---|
| RS2_HUMAN | P60866 | RPS20 | 8,8 | 2,310E-06 |
| RS20_HUMAN | P63220 | RPS21 | 55,0 | 1,980E-10 |
| RS21_HUMAN | P62266 | RPS23 | 48,3 | 1,220E-10 |
| RS23_HUMAN | P62847 | RPS24 | 37,5 | 4,020E-10 |
| RS24_HUMAN | P62851 | RPS25 | 39,6 | 2,540E-10 |
| RS25_HUMAN | P62854 | RPS26 | 47,9 | 6,440E-11 |
| RS26_HUMAN | P62979 | RPS27A UBA80 UBCEP1 | 48,3 | 4,520E-11 |
| RS27_HUMAN | Q71UM5 | RPS27L | 35,0 | 2,350E-08 |
| RS27A_HUMAN | P42677 | RPS27 MPS1 | 52,6 | 2,820E-11 |
| RS27L_HUMAN | P62857 | RPS28 | 32,8 | 1,090E-08 |
| RS28_HUMAN | P62273 | RPS29 | 56,6 | 4,510E-10 |
| RS29_HUMAN | P15880 | RPS2 RPS4 | 43,4 | 5,420E-11 |
| RS3_HUMAN | P62861 | FAU | 9,0 | 4,850E-05 |
| RS30_HUMAN | P61247 | RPS3A FTE1 MFTL | 39,7 | 4,580E-09 |
| RS3A_HUMAN | P23396 | RPS3 OK/SW-cl.26 | 5,8 | 7,770E-06 |
| RS4X_HUMAN | P62701 | RPS4X CCG2 RPS4 SCAR | 30,3 | 1,470E-07 |
| RS5_HUMAN | P46782 | RPS5 | 31,6 | 2,460E-08 |
| RS6_HUMAN | P62753 | RPS6 OK/SW-cl.2 | 35,5 | 2,600E-10 |
| RS7_HUMAN | P62081 | RPS7 | 34,1 | 3,070E-08 |
| RS8_HUMAN | P62241 | RPS8 OK/SW-cl.83 | 29,6 | 1,920E-08 |
| RS9_HUMAN | P46781 | RPS9 | 36,9 | 3,080E-08 |
| RSSA_HUMAN | P08865 | RPSA LAMBR LAMR1 | 17,5 | 9,220E-06 |
| RT05_HUMAN | P82675 | MRPS5 | 5,4 | 1,125E-02 |
| RT18B_HUMAN | Q9Y676 | MRPS18B C6orf14 HSPC183 PTD017 | 17,0 | 8,940E-05 |
| RT22_HUMAN | P82650 | MRPS22 C3orf5 RPMS22 GK002 | 4,0 | 6,482E-04 |
| RT23_HUMAN | Q9Y3D9 | MRPS23 CGI-138 HSPC329 | 7,5 | 1,122E-03 |
| RT27_HUMAN | Q92552 | MRPS27 KIAA0264 | 21,1 | 1,117E-02 |
| RT28_HUMAN | Q9Y2Q9 | MRPS28 MRPS35 HSPC007 | 14,6 | 1,563E-03 |
| RT34_HUMAN | P82930 | MRPS34 | 9,5 | 1,104E-04 |
| RTCB_HUMAN | Q9Y3I0 | RTCB C22orf28 HSPC117 | 6,0 | 2,820E-11 |
| RU17_HUMAN | P08621 | SNRNP70 RNPU1Z RPU1 SNRP70 U1AP1 | 13,0 | 4,390E-08 |
| RUVB1_HUMAN | Q9Y265 | RUVBL1 INO80H NMP238 TIP49 TIP49A | 7,5 | 1,190E-06 |
| RUVB2_HUMAN | Q9Y230 | RUVBL2 INO80J TIP48 TIP49B CGI-46 | 6,0 | 1,340E-06 |
| RUXE_HUMAN | P62304 | SNRPE | 15,1 | 6,430E-09 |
| RUXF_HUMAN | P62306 | SNRPF PBSCF | 15,0 | 1,250E-08 |
| S61A1_HUMAN | P61619 | SEC61A1 SEC61A | 18,9 | 2,290E-08 |
| SAFB1_HUMAN | Q15424 | SAFB HAP HET SAFB1 | 3,9 | 1,850E-06 |
| SART3_HUMAN | Q15020 | SART3 KIAA0156 TIP110 | 6,2 | 5,215E-04 |
| SC61B_HUMAN | P60468 | SEC61B | 36,4 | 8,060E-09 |
| SDA1_HUMAN | Q9NVU7 | SDAD1 NUC130 | 16,2 | 5,250E-06 |
| SF3B1_HUMAN | O75533 | SF3B1 SAP155 | 9,2 | 2,986E-04 |
| SF3B3_HUMAN | Q15393 | SF3B3 KIAA0017 SAP130 | 4,9 | 3,280E-06 |
| SFPQ_HUMAN | P23246 | SFPQ PSF | 11,2 | 5,950E-10 |
| SK2L2_HUMAN | P42285 | SKIV2L2 KIAA0052 Mtr4 | 14,4 | 7,920E-08 |
| SKIV2_HUMAN | Q15477 | SKIV2L DDX13 SKI2W SKIV2 W | 9,9 | 5,900E-06 |
| SMBP2_HUMAN | P38935 | IGHMBP2 SMBP2 SMUBP2 | 7,1 | 5,800E-04 |

| | | | | |
|---|---|---|---|---|
| SMD1_HUMAN | P62314 | SNRPD1 | 15,2 | 8,060E-09 |
| SMD2_HUMAN | P62316 | SNRPD2 SNRPD1 | 8,8 | 3,244E-03 |
| SMD3_HUMAN | P62318 | SNRPD3 | 15,5 | 8,920E-11 |
| SND1_HUMAN | Q7KZF4 | SND1 TDRD11 | 22,3 | 6,050E-11 |
| SNRPA_HUMAN | P09012 | SNRPA | 13,0 | 1,450E-08 |
| SPAS2_HUMAN | Q86XZ4 | SPATS2 SCR59 SPATA10 Nbla00526 | 10,6 | 1,120E-05 |
| SPB1_HUMAN | Q8IY81 | FTSJ3 SB92 | 10,2 | 1,880E-09 |
| SPS2L_HUMAN | Q9NUQ6 | SPATS2L DNAPTP6 SP1224 | 18,3 | 1,220E-10 |
| SRP09_HUMAN | P49458 | SRP9 | 8,8 | 3,500E-07 |
| SRP14_HUMAN | P37108 | SRP14 | 43,2 | 1,020E-08 |
| SRP54_HUMAN | P61011 | SRP54 | 6,4 | 1,860E-05 |
| SRP68_HUMAN | Q9UHB9 | SRP68 | 12,2 | 3,650E-07 |
| SRP72_HUMAN | O76094 | SRP72 | 5,0 | 4,437E-04 |
| SRPK1_HUMAN | Q96SB4 | SRPK1 | 14,2 | 3,168E-02 |
| SRPK2_HUMAN | P78362 | SRPK2 | 15,6 | 6,750E-08 |
| SRSF1_HUMAN | Q07955 | SRSF1 ASF SF2 SF2P33 SFRS1 OK/SW-cl.3 | 14,3 | 6,000E-09 |
| SRSF3_HUMAN | P84103 | SRSF3 SFRS3 SRP20 | 22,8 | 1,430E-09 |
| SRSF4_HUMAN | Q08170 | SRSF4 SFRS4 SRP75 | 12,6 | 2,340E-05 |
| SRSF5_HUMAN | Q13243 | SRSF5 HRS SFRS5 SRP40 | 19,9 | 3,220E-07 |
| SRSF6_HUMAN | Q13247 | SRSF6 SFRS6 SRP55 | 20,2 | 7,530E-09 |
| SRSF7_HUMAN | Q16629 | SRSF7 SFRS7 | 8,9 | 3,908E-04 |
| SSF1_HUMAN | Q9NQ55 | PPAN BXDC3 SSF1 | 11,6 | 5,050E-07 |
| STAU2_HUMAN | Q9NUL3 | STAU2 | 6,6 | 2,230E-05 |
| SULF1_HUMAN | Q8IWU6 | SULF1 KIAA1077 | 13,7 | 1,390E-08 |
| SYDC_HUMAN | P14868 | DARS PIG40 | 3,9 | 5,940E-06 |
| SYEP_HUMAN | P07814 | EPRS GLNS PARS QARS QPRS PIG32 | 4,9 | 6,430E-09 |
| SYFA_HUMAN | Q9Y285 | FARSA FARS FARSL FARSLA | 12,6 | 4,860E-08 |
| SYFB_HUMAN | Q9NSD9 | FARSB FARSLB FRSB HSPC173 | 13,7 | 2,770E-09 |
| SYIC_HUMAN | P41252 | IARS | 4,5 | 8,170E-09 |
| SYK_HUMAN | Q15046 | KARS KIAA0070 | 6,6 | 9,680E-06 |
| SYLC_HUMAN | Q9P2J5 | LARS KIAA1352 | 3,5 | 5,249E-03 |
| SYMC_HUMAN | P56192 | MARS | 3,6 | 2,000E-05 |
| SYNM_HUMAN | Q96I59 | NARS2 | 12,4 | 1,780E-06 |
| SYQ_HUMAN | P47897 | QARS | 4,2 | 1,410E-07 |
| SYRC_HUMAN | P54136 | RARS | 4,9 | 2,490E-07 |
| SYYC_HUMAN | P54577 | YARS | 8,7 | 1,660E-06 |
| TADBP_HUMAN | Q13148 | TARDBP TDP43 | 15,6 | 2,720E-05 |
| TBL2_HUMAN | Q9Y4P3 | TBL2 WBSCR13 UNQ563/PRO1125 | 15,4 | 1,560E-08 |
| TF3C1_HUMAN | Q12789 | GTF3C1 | 4,0 | 1,666E-02 |
| THOC4_HUMAN | Q86V81 | ALYREF ALY BEF THOC4 | 12,6 | 2,090E-09 |
| TOP1_HUMAN | P11387 | TOP1 | 15,1 | 1,020E-06 |
| TR150_HUMAN | Q9Y2W1 | THRAP3 TRAP150 | 3,2 | 9,509E-03 |
| TRA2B_HUMAN | P62995 | TRA2B SFRS10 | 8,9 | 1,058E-04 |
| TRI56_HUMAN | Q9BRZ2 | TRIM56 RNF109 | 8,8 | 1,080E-06 |
| TRIPC_HUMAN | Q14669 | TRIP12 KIAA0045 ULF | 9,8 | 1,340E-07 |
| TRM1L_HUMAN | Q7Z2T5 | TRMT1L C1orf25 TRM1L MSTP070 | 11,2 | 2,250E-09 |

| | | | | |
|---|---|---|---|---|
| TSP1_HUMAN | P07996 | THBS1 TSP TSP1 | 5,5 | 2,670E-07 |
| TTC37_HUMAN | Q6PGP7 | TTC37 KIAA0372 | 16,3 | 5,920E-10 |
| U2AF2_HUMAN | P26368 | U2AF2 U2AF65 | 7,0 | 1,780E-08 |
| U3IP2_HUMAN | O43818 | RRP9 RNU3IP2 U355K | 14,0 | 6,890E-07 |
| U520_HUMAN | O75643 | SNRNP200 ASCC3L1 HELIC2 KIAA0788 | 6,4 | 1,350E-06 |
| U5S1_HUMAN | Q15029 | EFTUD2 KIAA0031 SNRP116 | 7,5 | 1,450E-07 |
| VIGLN_HUMAN | Q00341 | HDLBP HBP VGL | 6,1 | 1,930E-06 |
| WDR36_HUMAN | Q8NI36 | WDR36 | 10,1 | 3,908E-04 |
| WIBG_HUMAN | Q9BRP8 | WIBG PYM | 14,0 | 1,400E-06 |
| XRCC5_HUMAN | P13010 | XRCC5 G22P2 | 4,0 | 6,920E-05 |
| XRCC6_HUMAN | P12956 | XRCC6 G22P1 | 6,3 | 4,690E-06 |
| XRN2_HUMAN | Q9H0D6 | XRN2 | 4,4 | 2,106E-03 |
| YBOX1_HUMAN | P67809 | YBX1 NSEP1 YB1 | 18,1 | 1,990E-06 |
| YBOX3_HUMAN | P16989 | YBX3 CSDA DBPA | 19,7 | 1,680E-06 |
| YTDC2_HUMAN | Q9H6S0 | YTHDC2 | 8,9 | 5,600E-06 |
| ZCCHV_HUMAN | Q7Z2W4 | ZC3HAV1 ZC3HDC2 PRO1677 | 12,2 | 7,810E-08 |
| ZFP91_HUMAN | Q96JP5 | ZFP91 ZNF757 FKSG11 | 23,8 | 3,970E-08 |
| ZFR_HUMAN | Q96KR1 | ZFR | 14,5 | 4,740E-07 |
| ZN326_HUMAN | Q5BKZ1 | ZNF326 ZIRD | 14,8 | 3,640E-07 |
| ZN622_HUMAN | Q969S3 | ZNF622 ZPR9 | 16,7 | 2,700E-10 |

**Supplementary Table 1.** Proteins enriched in Chemical Proteomics analysis

From pulldown experiments with active and inactive compound, 430 proteins were identified showing ≥1.5-fold enrichment for the Active compound (adjusted p≤0.05). Using the GO_SLIM collection of gene ontology annotations (http://ceur-ws.org/Vol-1546/paper_44.pdf), enrichment for 8 pathway categories was found based on Fisher's exact test (cutoff for p<0.001), illustrated by negative logarithm of the enrichment p value on the x axis of Figure 1E. Reasoning that proteins involved in the translational machinery would be associated through their interactions with RNA, a second list of 339 proteins was created by manually removing ribosomal proteins form the 430 proteins. Repeating the enrichment analysis, smaller enrichment p values were obtained with the exception of the "RNA splicing" category (y axis in Figure 1E), which scored substantially better. We conclude that this category is the strongest hit independent of the (inevitable) presence of ribosomal proteins in our data set.

| ΔPSI | SMN-C3 | NVS-SM1 |
|---|---|---|
| ABCB8 | 0,398 | 0,670 |
| ANKRD36 | 0,035 | 0,578 |
| APLP2 | 0,270 | 0,466 |
| ARHGAP12 | 0,016 | 0,438 |
| ARMCX6 | 0,010 | 0,499 |
| ASAP1 | 0,355 | 0,733 |
| ATG5 | 0,027 | 0,446 |
| AXIN1 | 0,069 | 0,665 |
| BIRC6 | 0,039 | 0,563 |
| C1orf86 | 0,043 | 0,572 |
| CDC42BPA | 0,146 | 0,560 |
| CLTA | 0,239 | 0,473 |
| DYRK1A | 0,007 | 0,414 |
| ERGIC3 | 0,035 | 0,439 |
| FBXL6 | 0,461 | 0,037 |
| FOXM1 | 0,417 | 0,470 |
| GGCT | 0,455 | 0,362 |
| KAT6B | 0,047 | 0,707 |
| KDM6A | 0,082 | 0,404 |
| KIF3A | 0,219 | 0,681 |
| KMT2D | 0,048 | 0,647 |
| LARP7 | 0,404 | 0,864 |
| LYRM1 | 0,139 | 0,686 |
| MADD | 0,073 | 0,797 |
| MAN2C1 | 0,461 | 0,033 |
| MRPL55 | 0,448 | 0,013 |
| MYCBP2 | 0,111 | 0,511 |
| MYO9B | 0,506 | 0,031 |
| PNISR | 0,028 | 0,765 |
| RAP1A | 0,246 | 0,504 |
| RAPGEF1 | 0,552 | 0,402 |
| SENP6 | 0,364 | 0,567 |
| SH3YL1 | 0,131 | 0,566 |
| SLC25A17 | 0,644 | 0,481 |
| SMN2 | 0,429 | 0,537 |
| SREK1 | 0,021 | 0,466 |
| STRN3 | 0,556 | 0,860 |
| TAF2 | 0,217 | 0,710 |
| TMEM134 | 0,444 | 0,009 |
| VPS29 | 0,422 | 0,671 |
| ZFAND1 | 0,024 | 0,408 |
| ZNF431 | 0,111 | 0,468 |

**Supplementary Table 2.** Splicing changes from RNAseq study

Data express changes in SMN2 FL/Δ7 splicing ratio upon compound treatment as delta % spliced in (ΔPSI), shown in Fig. 3B. Data were averaged from five independent samples.

| | | abs(logFC) | |
|---|---|---|---|
| **Name** | **Description** | **SMN-C3** | **NVS-SM1** |
| 100507053 | - | 1,316 | 1,967 |
| 100507424 | - | 0,668 | 1,451 |
| 101928588 | - | 0,595 | 1,746 |
| 101928711 | - | 0,59 | 1,828 |
| 100289187 | - | 0,146 | 1,511 |
| 653513 | - | 0,134 | 1,220 |
| 100288152 | - | 0,11 | 1,179 |
| 25845 | - | 0,049 | 1,142 |
| 4363 | ABCC1 | 0,053 | 0,966 |
| 59 | ACTA2 | 0,292 | 1,141 |
| 161823 | ADAL | 0,238 | 1,302 |
| 8038 | ADAM12 | 0,002 | 1,868 |
| 9510 | ADAMTS1 | 0,04 | 1,137 |
| 11096 | ADAMTS5 | 0,072 | 1,380 |
| 118 | ADD1 | 0,027 | 0,989 |
| 57211 | ADGRG6 | 0,052 | 1,128 |
| 137872 | ADHFE1 | 0,245 | 1,491 |
| 55750 | AGK | 0,182 | 1,159 |
| 8540 | AGPS | 0,33 | 1,842 |
| 651746 | ANKRD33B | 0,017 | 1,145 |
| 311 | ANXA11 | 0,045 | 1,142 |
| 309 | ANXA6 | 0,068 | 1,224 |
| 84159 | ARID5B | 0,082 | 0,906 |
| 132946 | ARL9 | 0,962 | 1,627 |
| 51566 | ARMCX3 | 0,516 | 1,252 |
| 50807 | ASAP1 | 0,156 | 1,729 |
| 41 | ASIC1 | 0,246 | 2,102 |
| 54454 | ATAD2B | 0,558 | 0,697 |
| 594 | BCKDHB | 0,095 | 0,976 |
| 440603 | BCL2L15 | 0,204 | 4,557 |
| 618 | BCYRN1 | 1,002 | 1,991 |
| 7832 | BTG2 | 0,121 | 0,938 |
| 64115 | C10ORF54 | 0,147 | 1,068 |
| 85016 | C11ORF70 | 0,084 | 1,177 |
| 51501 | C11ORF73 | 0,958 | 0,945 |
| 115749 | C12ORF56 | 0,125 | 1,522 |
| 126526 | C19ORF47 | 0,353 | 1,643 |
| 54969 | C4ORF27 | 0,714 | 1,487 |
| 782 | CACNB1 | 0,429 | 2,565 |
| 23705 | CADM1 | 0,235 | 1,053 |
| 23066 | CAND2 | 1,038 | 1,721 |
| 22794 | CASC3 | 0,332 | 0,563 |
| 899 | CCNF | 0,014 | 0,864 |
| 83879 | CDCA7 | 0,561 | 1,734 |

| | | | |
|---|---|---|---|
| 54901 | CDKAL1 | 0,274 | 0,977 |
| 57214 | CEMIP | 0,175 | 2,044 |
| 9859 | CEP170 | 0,139 | 1,833 |
| 3075 | CFH | 0,158 | 1,071 |
| 57396 | CLK4 | 0,398 | 0,650 |
| 8418 | CMAHP | 0,523 | 1,458 |
| 124817 | CNTD1 | 0,096 | 2,575 |
| 1301 | COL11A1 | 0,212 | 1,451 |
| 1303 | COL12A1 | 0,069 | 1,698 |
| 1289 | COL5A1 | 0,046 | 0,953 |
| 50509 | COL5A3 | 0,023 | 0,989 |
| 1295 | COL8A1 | 0,044 | 1,204 |
| 81035 | COLEC12 | 0,138 | 1,449 |
| 1311 | COMP | 0,213 | 1,519 |
| 51200 | CPA4 | 0,355 | 1,342 |
| 10404 | CPQ | 0,401 | 1,166 |
| 83716 | CRISPLD2 | 0,101 | 1,103 |
| 9244 | CRLF1 | 0,054 | 0,932 |
| 51084 | CRYL1 | 0,353 | 1,953 |
| 1503 | CTPS1 | 0,364 | 0,441 |
| 1523 | CUX1 | 0,046 | 0,902 |
| 80777 | CYB5B | 0,004 | 1,485 |
| 51700 | CYB5R2 | 0,227 | 1,829 |
| 114757 | CYGB | 0,207 | 1,236 |
| 1545 | CYP1B1 | 0,296 | 2,496 |
| 1634 | DCN | 0,131 | 0,924 |
| 23142 | DCUN1D4 | 0,367 | 0,356 |
| 115265 | DDIT4L | 0,18 | 1,067 |
| 11325 | DDX42 | 0,117 | 1,131 |
| 79009 | DDX50 | 0,194 | 0,979 |
| 8560 | DEGS1 | 0,022 | 1,017 |
| 57706 | DENND1A | 1,284 | 1,426 |
| 23258 | DENND5A | 0,74 | 2,090 |
| 494513 | DFNB59 | 0,172 | 1,198 |
| 1606 | DGKA | 0,268 | 0,938 |
| 1719 | DHFR | 0,436 | 2,079 |
| 81624 | DIAPH3 | 0,862 | 3,346 |
| 9077 | DIRAS3 | 0,044 | 1,012 |
| 115752 | DIS3L | 0,059 | 0,910 |
| 9231 | DLG5 | 0,051 | 1,276 |
| 51277 | DNAJC27 | 0,701 | 1,396 |
| 1793 | DOCK1 | 0,438 | 0,660 |
| 139818 | DOCK11 | 0,031 | 1,704 |
| 1780 | DYNC1I1 | 0,078 | 1,720 |
| 199221 | DZIP1L | 0,089 | 1,266 |
| 1879 | EBF1 | 0,038 | 1,119 |

| | | | |
|---|---|---|---|
| 2202 | EFEMP1 | 0,073 | 1,433 |
| 8891 | EIF2B3 | 0,602 | 2,126 |
| 2006 | ELN | 0,026 | 1,077 |
| 26610 | ELP4 | 0,076 | 2,330 |
| 196047 | EMX2OS | 0,036 | 1,161 |
| 5167 | ENPP1 | 0,03 | 0,918 |
| 2034 | EPAS1 | 0,051 | 0,984 |
| 1161 | ERCC8 | 0,202 | 0,968 |
| 11082 | ESM1 | 0,438 | 2,206 |
| 132884 | EVC2 | 0,176 | 1,180 |
| 2149 | F2R | 0,217 | 1,562 |
| 51313 | FAM198B | 0,215 | 1,617 |
| 115572 | FAM46B | 0,177 | 1,079 |
| 2191 | FAP | 0,273 | 1,139 |
| 10160 | FARP1 | 0,063 | 1,349 |
| 2199 | FBLN2 | 0,121 | 1,460 |
| 2201 | FBN2 | 0,119 | 1,620 |
| 26267 | FBXO10 | 0,513 | 0,524 |
| 26268 | FBXO9 | 0,27 | 2,108 |
| 2241 | FER | 0,406 | 1,603 |
| 2263 | FGFR2 | 0,11 | 1,396 |
| 8061 | FOSL1 | 0,371 | 0,342 |
| 80144 | FRAS1 | 0,025 | 1,837 |
| 79690 | GAL3ST4 | 0,176 | 0,927 |
| 2581 | GALC | 1,559 | 1,312 |
| 2627 | GATA6 | 0,203 | 1,214 |
| 26301 | GBGT1 | 0,101 | 1,159 |
| 2650 | GCNT1 | 0,023 | 1,165 |
| 2776 | GNAQ | 0,085 | 1,724 |
| 2804 | GOLGB1 | 0,218 | 0,917 |
| 51704 | GPRC5B | 0,269 | 1,288 |
| 283464 | GXYLT1 | 0,093 | 1,372 |
| 3038 | HAS3 | 0,281 | 3,333 |
| 84868 | HAVCR2 | 0,187 | 5,032 |
| 10014 | HDAC5 | 0,01 | 1,476 |
| 9843 | HEPH | 0,117 | 0,932 |
| 6596 | HLTF | 0,898 | 1,082 |
| 3162 | HMOX1 | 0,108 | 2,069 |
| 84376 | HOOK3 | 0,06 | 1,032 |
| 51144 | HSD17B12 | 0,569 | 1,981 |
| 3305 | HSPA1L | 0,163 | 2,486 |
| 10553 | HTATIP2 | 0,073 | 0,944 |
| 3064 | HTT | 0,551 | 1,288 |
| 3482 | IGF2R | 0,098 | 1,517 |
| 3486 | IGFBP3 | 0,082 | 1,012 |
| 9118 | INA | 0,005 | 1,249 |

| | | | |
|---|---|---|---|
| 27152 | INTU | 0,334 | 1,084 |
| 84223 | IQCG | 0,013 | 1,024 |
| 22801 | ITGA11 | 0,144 | 1,427 |
| 3706 | ITPKA | 0,051 | 1,555 |
| 7403 | KDM6A | 0,23 | 1,335 |
| 2531 | KDSR | 0,042 | 1,105 |
| 57604 | KIAA1456 | 0,124 | 1,168 |
| 57608 | KIAA1462 | 0,052 | 1,050 |
| 57650 | KIAA1524 | 0,477 | 2,072 |
| 80856 | KIAA1715 | 0,378 | 1,161 |
| 3815 | KIT | 0,214 | 1,801 |
| 3855 | KRT7 | 0,086 | 1,099 |
| 81851 | KRTAP1-1 | 0,072 | 0,975 |
| 83895 | KRTAP1-5 | 0,062 | 1,222 |
| 83746 | L3MBTL2 | 0,038 | 0,943 |
| 55366 | LGR4 | 0,203 | 0,938 |
| 79940 | LINC00472 | 0,325 | 1,415 |
| 100505566 | LINC00578 | 0,028 | 1,221 |
| 646324 | LINC00607 | 0,14 | 1,198 |
| 100652988 | LINC00702 | 0,007 | 1,342 |
| 25802 | LMOD1 | 0,213 | 1,192 |
| 987 | LRBA | 0,016 | 0,990 |
| 4038 | LRP4 | 0,07 | 1,439 |
| 2615 | LRRC32 | 0,09 | 1,530 |
| 127495 | LRRC39 | 0,908 | 2,805 |
| 4045 | LSAMP | 0,185 | 2,154 |
| 4060 | LUM | 0,208 | 1,287 |
| 116372 | LYPD1 | 0,065 | 0,909 |
| 57149 | LYRM1 | 0,015 | 0,886 |
| 9935 | MAFB | 0,063 | 1,192 |
| 10905 | MAN1A2 | 0,175 | 1,257 |
| 4124 | MAN2A1 | 0,044 | 0,949 |
| 5603 | MAPK13 | 0,018 | 0,856 |
| 5648 | MASP1 | 0,292 | 1,341 |
| 84935 | MEDAG | 1,097 | 2,649 |
| 1953 | MEGF6 | 0,103 | 0,996 |
| 51072 | MEMO1 | 0,381 | 1,173 |
| 693197 | MIR612 | 0,692 | 2,380 |
| 8028 | MLLT10 | 0,058 | 0,878 |
| 4319 | MMP10 | 0,431 | 0,871 |
| 10893 | MMP24 | 0,107 | 1,317 |
| 64210 | MMS19 | 0,175 | 1,064 |
| 4330 | MN1 | 0,216 | 1,052 |
| 26002 | MOXD1 | 0,126 | 0,921 |
| 10335 | MRVI1 | 0,27 | 1,184 |
| 51001 | MTERF3 | 0,626 | 0,644 |

| | | | |
|---|---|---|---|
| 25878 | MXRA5 | 0,126 | 2,191 |
| 4642 | MYO1D | 0,158 | 2,088 |
| 8883 | NAE1 | 0,047 | 0,895 |
| 162417 | NAGS | 0,251 | 1,206 |
| 79625 | NDNF | 0,041 | 1,154 |
| 54492 | NEURL1B | 0,254 | 0,965 |
| 57486 | NLN | 0,127 | 1,591 |
| 4854 | NOTCH3 | 0,033 | 0,960 |
| 10886 | NPFFR2 | 0,752 | 0,717 |
| 22854 | NTNG1 | 0,282 | 1,023 |
| 100506658 | OCLN | 0,033 | 1,366 |
| 64064 | OXCT2 | 0,134 | 1,230 |
| 167153 | PAPD4 | 1,155 | 1,492 |
| 64081 | PBLD | 0,08 | 1,158 |
| 5108 | PCM1 | 0,142 | 1,914 |
| 5137 | PDE1C | 0,164 | 1,145 |
| 8654 | PDE5A | 0,077 | 1,282 |
| 5159 | PDGFRB | 0,087 | 1,008 |
| 23047 | PDS5B | 0,065 | 1,673 |
| 23042 | PDXDC1 | 1,673 | 2,015 |
| 375033 | PEAR1 | 0,165 | 1,144 |
| 5184 | PEPD | 0,207 | 0,994 |
| 26147 | PHF19 | 0,382 | 0,042 |
| 55300 | PI4K2B | 0,165 | 1,133 |
| 5295 | PIK3R1 | 0,204 | 0,941 |
| 11040 | PIM2 | 0,061 | 0,971 |
| 23760 | PITPNB | 0,693 | 2,516 |
| 83394 | PITPNM3 | 0,063 | 1,350 |
| 5328 | PLAU | 0,606 | 0,645 |
| 26499 | PLEK2 | 0,054 | 0,888 |
| 22874 | PLEKHA6 | 0,043 | 1,425 |
| 130271 | PLEKHH2 | 0,135 | 1,548 |
| 10154 | PLXNC1 | 0,214 | 1,714 |
| 5378 | PMS1 | 0,777 | 1,319 |
| 127435 | PODN | 0,014 | 1,197 |
| 353497 | POLN | 1,108 | 0,544 |
| 25885 | POLR1A | 0,004 | 1,651 |
| 10631 | POSTN | 0,225 | 0,884 |
| 5530 | PPP3CA | 0,093 | 1,333 |
| 5578 | PRKCA | 0,021 | 0,992 |
| 5591 | PRKDC | 0,111 | 1,726 |
| 5592 | PRKG1 | 0,147 | 1,131 |
| 5961 | PRPH2 | 0,241 | 1,051 |
| 100507062 | PSMD6-AS2 | 0,201 | 1,005 |
| 5740 | PTGIS | 0,085 | 1,433 |
| 5806 | PTX3 | 0,064 | 1,035 |

| | | | |
|---|---|---|---|
| 27314 | RAB30 | 0,141 | 1,066 |
| 5917 | RARS | 0,009 | 0,908 |
| 5932 | RBBP8 | 0,131 | 1,816 |
| 64080 | RBKS | 0,146 | 1,186 |
| 1104 | RCC1 | 0,329 | 3,358 |
| 5962 | RDX | 0,11 | 2,309 |
| 64326 | RFWD2 | 0,435 | 1,943 |
| 101929302 | RFX3-AS1 | 1,092 | 2,386 |
| 28984 | RGCC | 0,304 | 1,316 |
| 51136 | RNFT1 | 0,112 | 1,127 |
| 4919 | ROR1 | 0,121 | 1,224 |
| 201965 | RWDD4 | 0,18 | 0,946 |
| 619383 | SCARNA9 | 1,339 | 1,209 |
| 6341 | SCO1 | 0,137 | 1,280 |
| 26984 | SEC22A | 0,69 | 0,833 |
| 57619 | SHROOM3 | 0,187 | 1,055 |
| 57181 | SLC39A10 | 0,061 | 0,930 |
| 83959 | SLC4A11 | 0,127 | 1,634 |
| 23657 | SLC7A11 | 0,006 | 0,908 |
| 6550 | SLC9A3 | 0,354 | 1,042 |
| 6586 | SLIT3 | 0,009 | 1,054 |
| 100271836 | SMG1P3 | 0,624 | 0,790 |
| 6525 | SMTN | 0,274 | 1,123 |
| 64754 | SMYD3 | 0,1 | 0,961 |
| 25992 | SNED1 | 0,022 | 1,164 |
| 100507246 | SNHG16 | 0,435 | 1,062 |
| 57537 | SORCS2 | 0,109 | 0,949 |
| 57213 | SPRYD7 | 0,391 | 0,849 |
| 58472 | SQRDL | 0,651 | 0,876 |
| 6772 | STAT1 | 0,553 | 1,162 |
| 261729 | STEAP2 | 0,916 | 2,899 |
| 55351 | STK32B | 0,004 | 1,001 |
| 29888 | STRN4 | 0,014 | 1,445 |
| 412 | STS | 0,114 | 1,228 |
| 29091 | STXBP6 | 0,06 | 0,991 |
| 23213 | SULF1 | 0,07 | 1,131 |
| 79987 | SVEP1 | 0,157 | 1,741 |
| 9144 | SYNGR2 | 0,08 | 1,463 |
| 11346 | SYNPO | 0,069 | 1,105 |
| 171024 | SYNPO2 | 0,211 | 1,849 |
| 4070 | TACSTD2 | 0 | 1,165 |
| 29114 | TAGLN3 | 0,253 | 1,142 |
| 79613 | TANGO6 | 0,01 | 1,059 |
| 6894 | TARBP1 | 0,659 | 1,401 |
| 441687 | TEX21P | 3,517 | 2,427 |
| 7039 | TGFA | 0,153 | 0,832 |

| | | | |
|---|---|---|---|
| 7043 | TGFB3 | 0,148 | 1,044 |
| 7052 | TGM2 | 0,043 | 0,993 |
| 63892 | THADA | 0,531 | 1,759 |
| 7058 | THBS2 | 0,157 | 1,364 |
| 284114 | TMEM102 | 0,151 | 1,019 |
| 338773 | TMEM119 | 0,197 | 1,077 |
| 100529211 | TMEM256-PLSCR3 | 0,668 | 1,867 |
| 757 | TMEM50B | 0,104 | 0,918 |
| 3371 | TNC | 0,092 | 0,959 |
| 388121 | TNFAIP8L3 | 0,092 | 1,097 |
| 8764 | TNFRSF14 | 0,208 | 1,419 |
| 64759 | TNS3 | 0,681 | 2,764 |
| 7148 | TNXB | 0,141 | 1,194 |
| 401505 | TOMM5 | 0,452 | 0,389 |
| 7187 | TRAF3 | 0,045 | 0,988 |
| 7223 | TRPC4 | 0,207 | 1,522 |
| 128553 | TSHZ2 | 0,028 | 1,224 |
| 90139 | TSPAN18 | 0,266 | 1,377 |
| 81629 | TSSK3 | 0,045 | 1,000 |
| 10628 | TXNIP | 0,21 | 1,275 |
| 219699 | UNC5B | 0,183 | 0,926 |
| 7405 | UVRAG | 0,123 | 0,997 |
| 100507347 | VIM-AS1 | 0,228 | 1,463 |
| 27072 | VPS41 | 0,041 | 1,441 |
| 128434 | VSTM2L | 0,111 | 1,863 |
| 23078 | VWA8 | 0,032 | 1,341 |
| 29062 | WDR91 | 0,273 | 0,895 |
| 8840 | WISP1 | 0,228 | 1,007 |
| 22803 | XRN2 | 0,376 | 2,311 |
| 150223 | YDJC | 0,067 | 0,840 |
| 26137 | ZBTB20 | 0,523 | 0,204 |
| 57684 | ZBTB26 | 0,269 | 1,649 |
| 284406 | ZFP82 | 0,224 | 0,963 |
| 7988 | ZNF212 | 0,041 | 0,892 |
| 284695 | ZNF326 | 0,352 | 0,135 |
| 59348 | ZNF350 | 0,178 | 0,936 |
| 7633 | ZNF79 | 0,041 | 1,075 |
| 116412 | ZNF837 | 2,469 | 3,044 |

**Supplementary Table 3.** Transcriptional changes from RNAseq study

Data express changes in transcription upon compound treatment as log2 fold change, shown in Fig. 3C. Data were averaged from five independent samples.

| **Inhibitor** | **Concentration** |
|---|---|
| Actinomycin D | 1µg/ml |
| Cordycepin (3-deoxyadenosine) | 500nM |
| α-Amanitin | 50µg/ml |
| Puromycin dihydrochloride | 10µg/ml |
| Pladienolide-B | 10µM |
| Isoginkgetin | 50µM |

**Supplementary Table 4.** Concentration of inhibitors used in cell experiments (Fig. 1, Supplementary Fig. 2).

| target name | primer/probe | sequence (5’ -> 3’) |
|---|---|---|
| SMN2 (full length) | forward primer (exon 7/8) | GCTCACATTCCTTAAATTAAGGAGAAA |
| | reverse primer (exon 8) | TCCAGATCTGTCTGATCGTTTCTT |
| | 5’FAM/3’BHQ1-probe (exon 8) | CTGGCATAGAGCAGCACTAAATGACACCAC |
| SMN2 (delta exon 7) | forward primer (exon 6/8) | TGGCTATCATACTGGCTATTATATGGAA |
| | reverse primer (exon 8) | TCCAGATCTGTCTGATCGTTTCTT |
| | 5’FAM/3’BHQ1-probe (exon 8) | CTGGCATAGAGCAGCACTAAATGACACCAC |
| STRN3 (full length) | forward primer (exon 8/9) | GGAAGAAAGGGGTGAAGAGG |
| | reverse primer (exon 9) | TGATTCCTGAAGGGATGTGG |
| | 5’FAM/3’BHQ1-UPL-probe (exon 9) | CTGGGAGA |
| STRN3 (delta exon 8/9) | forward primer (exon 7/10) | CAGAATGGGCTGAACCAATAA |
| | reverse primer (exon 10) | ACCGTCAAGTCTGCAAGGTC |
| | 5’FAM/3’BHQ1-probe (exon 10) | TCTGGAGGAGGCAAGTCATT |
| GAPDH | forward primer (exon 3) | CAACGGATTTGGTCGTATTGG |
| | reverse primer (exon 2/3) | TGATGGCAACAATATCCACTTTACC |
| | 5’VIC/3’TAMRA-probe (exon 3) | CGCCTGGTCACCAGGGCTGCT |

**Supplementary Table 5.** Taqman-based RT-qPCR and *SMN2*-specific primers and probes used to quantify mRNA levels of *SMN2*, STRN3 and *GAPDH* (Suplementary Fig. 4C)

| RNA | MW | Sequence |
|---|---|---|
| ESE1 | 5032.9 | 5’-AAA AAG AAG GAA GG-3’ |
| ESE2 | 5032.9 | 5’-AAA AAG AAG GAA GG-3’ |
| ESE3 | 4130.3 | 5’-ACA UUC CUU AAA-3’ |
| ESE3_TSL2 | 8687 | 5’-UCUCAU UCC UUA AAU UAA GGA GUA AG-3’ |
| antisense TSL2 | 8640.9 | 5’-CUU ACU CCU UAA UUU AAG GAA UGU GA-3’ |
| Exon 7 | 21099.6 | 5‘-UAC AGG GUU UUA GAC AAA AUC AAA AAG AAG GAA GGU GCU CAC AUU CCU UAA AUU AAG GAG UAA G-3‘ |
| ISS-N2 | 14491.4 | 5‘-CAC UAG UAG GCA GAC CAG CAG ACU UUU UUU UAU UGU GAU AUG GG-3‘ |
| TSTL4/6 | 14745.6 | 5‘-AUA ACC UAG GCA UAC UGC ACU GUA CAC UCU GAC AUA UGA AGU GCU-3‘ |
| I7_c | 12450.2 | 5‘-CGU CAA GCC UCU GGU UCU AAU UUC UCA UUU GCA GGA AA-3‘ |
| TSL7 | 16129.5 | 5‘-CUA GUC AAG UUU AAC UGG UGU CCA CAG AGG ACA UGG UUU AAC UGG AAU U-3‘ |
| GA | 5064.9 | 5‘-GAG AGA GAG AGA GA-3‘ |
| GU | 4903.7 | 5‘-GUG UGU GUG UGU GU-3‘ |
| STRN3 “ESE” | 9745.9 | 5’-AAG AAG GAA CGA AAG GGG AAG AAA GGG G-3’ |

**Supplementary Table 6.** RNA sequences used for immobilization in surface plasmon resonance experiments on Biacore (Fig. 4b, 4C, 4D)

| Ingredient | µL/1 reaction |
|---|---|
| 5x transcription buffer (Roche Diagnostics, 11465384001) | 2.5 |
| 4 rNTP mix<br>(2.5 mM A, 2.5 mM C, 0.5 mM G, 0.5 mM U) | 2.5 |
| 100mM DTT | 1.25 |
| 10mM m7G(5′)P(5′)G-cap structure analog (New England Biolabs, S1407L) | 0.624 |
| RNase-In (Promega, N251B) | 1 |
| Water | 1.126 |
| $^{32}$P-UTP | 1.5 |

| **Reagent** | **Volume (µl)** |
|---|---|
| $H_2O$ | 8.15 |
| cDNA template | 1.5 |
| 10x PCR buffer (Applied Biosystems, R09952) | 1.25 |
| 10mM dNTP mix | 0.375 |
| 10pmol/µl primer F | 0.375 |
| 10pmol/µl primer R | 0.375 |
| [α-$^{32}$P]dCTP | 0.1 |
| AmpliTaq DNA polymerase (AB, 100020478) | 0.375 |
| **Total:** | **12.5** |

Program:

| Step | Temp (°C) | Time (mm:ss) |
|---|---|---|
| 1 | 94 | 03:00 |
| 2 | 94 | 00:34 |
| 3 | 55 | 00 :50 |
| 4 | 72 | 00 :36 |
| 5 | Go to step 2, 25 times | |
| 6 | 72 | 05 :00 |
| 7 | 4 | For ever |

Primer:

| | |
|---|---|
| T7_fw | Tacttaatacgactcactataggctagcctcg |
| Ex8_pCI-neo_rv | Aagtacttacctgtaacgcttcacattccagatctgtc |
| Ex7_pCI-neo_rv | aagtacttac tccttaatttaaggaatgtgagcaccttcc |

**Supplementary Table 7.** *In vitro* transcription buffer and radioactive PCR protocol for in vitro transcription assay in HEK293 cells (Figure 2G)
